# Diterpenoids and Triterpenoids from the Aerial Parts of *Isodon serra* and Their Biological Activities

**DOI:** 10.1021/acsomega.4c08821

**Published:** 2024-11-22

**Authors:** Wen-Jing Ren, Rong Jiang, Kei-Fong Ng, Meng-Yu Bao, Xiao-Mei Liu, Wei Zhang, Zhi-Hong Jiang, Yu-Hong Liu, Guo-Yuan Zhu

**Affiliations:** † State Key Laboratory of Quality Research in Chinese Medicine, Guangdong-Hong Kong-Macao Joint Laboratory of Respiratory Infectious Disease, Macau Institute for Applied Research in Medicine and Health, Macau University of Science and Technology, Macau 999078, People’s Republic of China; ‡ School of Pharmaceutical Sciences, Shandong University of Traditional Chinese Medicine, Jinan 250355, People’s Republic of China

## Abstract

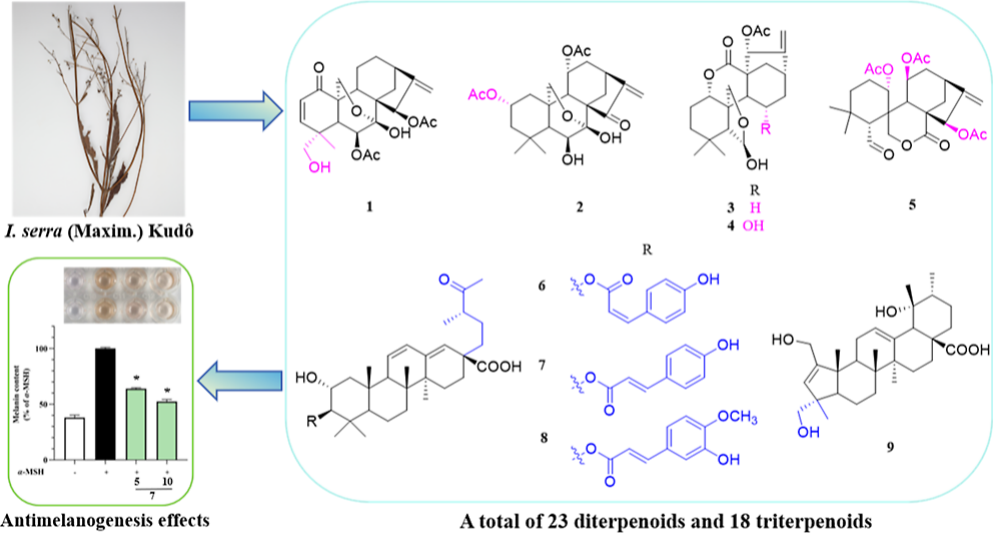

Five undescribed diterpenoids, serranins A–E (**1**–**5**), and four novel triterpenoids, serratic acids A–D (**6**–**9**), along with 32 known terpenoids (**10**–**41**) were isolated from the aerial parts of *Isodon serra*. The planar structures of **1**–**9** and their relative configurations were established on the basis of extensive spectroscopic analysis. Structurally, compounds **6**–**9** are the first examples of 18,19-*seco*-ursane *p*-coumaric or ferulic esters, while compounds **1**–**5** further enriched the plant’s diterpene profile. Bioactivity evaluation revealed that four diterpenoids (**2**, **10**, **12**, and **13**) exhibited potent cytotoxic activity against four cancer cell lines (B16–F10, A375, A549, and MDA-MB-231) with IC_50_ values below 10 μM. Remarkably, **7** and **38** exhibited a comparable antimelanogenesis effect to that of positive control (kojic acid) in B16–F10 cells.

## Introduction

*Isodon serra* (Maxim.) Kudo, a perennial plant named “*Xihuangcao*” in China, is mainly distributed in Hunan, Hubei, Sichuan, Yunnan, Guangdong, Jiangxi, and Fujian provinces, China. As a widely used Chinese folk medicine, *I. serra* has been popularly used to treat acute icteric hepatitis, arthritis, acute cholecystitis, enteritis, damp-heat dysentery, bruises, and health care.^1,2^ Some health-promoting Chinese patent medicine and beverages derived from this plant have also been developed, such as *Xihuangcao* granules, *Xihuangcao* tea bags, Xiaoyan Lidan Tablets, and Fufangdantong Tablets, possessing significant liver and cholecyst protection and anticancer effects.^3^ In addition, *I. serra* is an important ingredient in Cantonese herbal tea which is used for daily health care. Due to its wide use in herbal medicine and health foods, phytochemical investigation on *I. serra* has also attracted continuous attention. To date, approximately 150 compounds including a variety of *ent*-kaurane types diterpenoids, triterpenoids, flavonoids, and phenols have been isolated extensively from this species over the past few decades.^4,5^ Previous pharmacological studies suggested that the above compounds displayed cytotoxic, antiinflammatory, antiviral, antibacterial, and antioxidant activities.^4,5^

**Figure 1:**
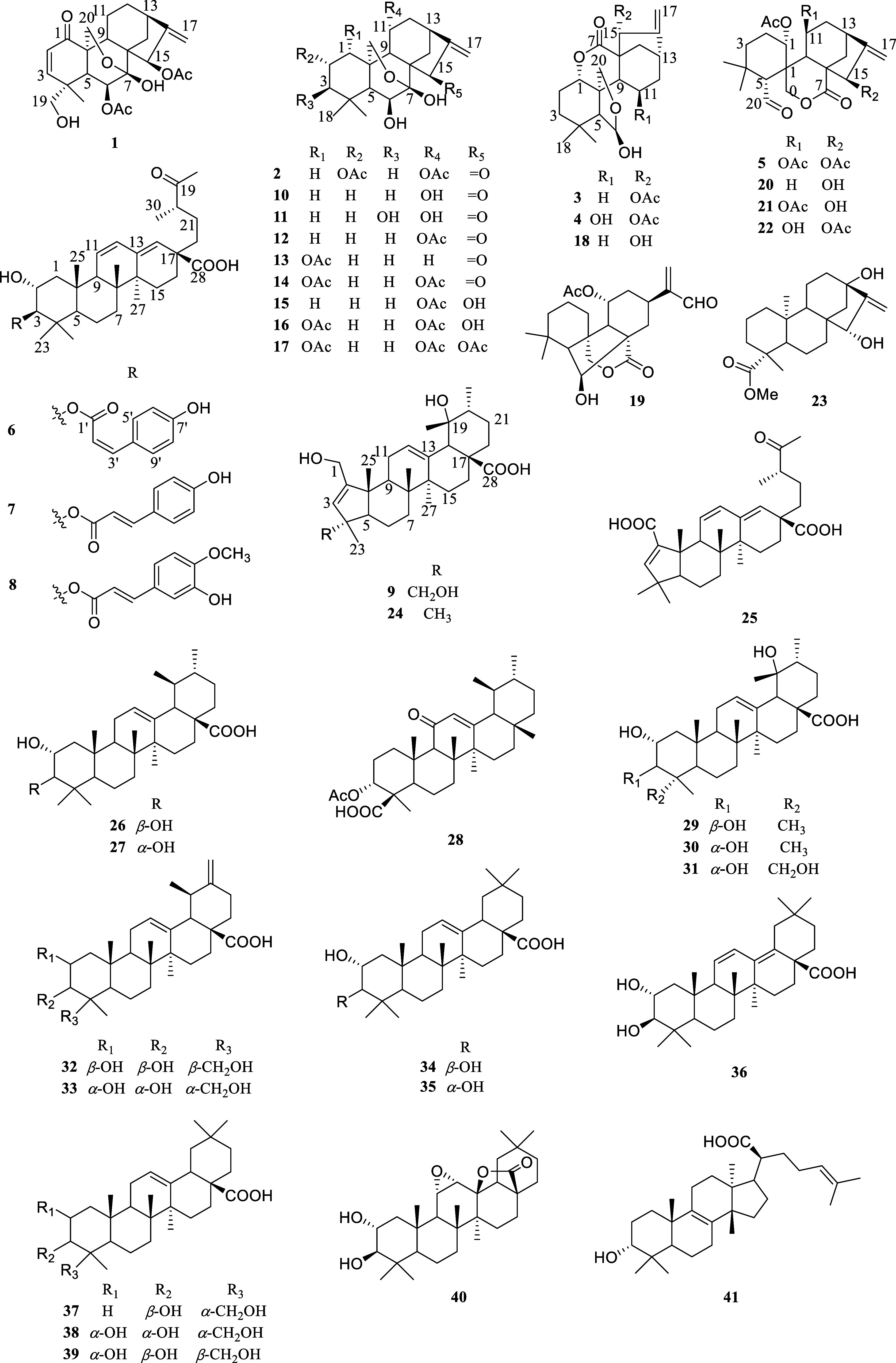
Structures of compounds **1**–**41**.

Our previous study on the rhizomes of *Isodon amethystoides*,^6^ a medicinal plant belonging to the genus *Isodon*, reported an unprecedented tetracyclic triterpenoid and six new diterpenoids together with 31 known terpenoids. Among them, several diterpenoids significantly inhibited nitric oxide (NO) production in LPS-stimulated RAW264.7 cells by the downregulation of LPS-induced iNOS protein expression, and a few diterpenoids displayed cytotoxicity against lung cancer and breast cancer lines. To search for more structurally intriguing and bioactive terpenoids from *Isodon* species, the chemical constituents of the aerial parts of *I. serra* have been investigated in this study, which led to the isolation and characterization of five undescribed diterpenoids (**1**–**5**), four new triterpenoids (**6**–**9**), and 32 known terpenoids (**10**–**41**) (Figure 1). Herein, details of the isolation, structure characterization, and bioactivities of these compounds are presented.

## Results and Discussion

The 80% EtOH extract of the aerial parts of *I. serra* (15 kg) was partitioned with petroleum ether (PE) and EtOAc. These fractions were then subject to silica gel column chromatography, MPLC, and HPLC to obtain nine new (**1**–**9**) and 32 known compounds (**10**–**41**) (Figure 1). The known compounds were identified as 6β,7β,11α-trihydroxy-7α,20-epoxy-*ent*-kaur-16-en-15-one (**10**),^7^ taibaihenryiin B (**11**),^8^ longikaurin E (**12**),^9^ effusanin B (**13**),^10^ shikokianin (**14**),^11^ isodoternifolin B (**15**),^12^ rabdosichuanin D (**16**),^13^ rabdosianin A (**17**),^14^ 6α,15β-dihydroxy-6,20-epoxy-emein-16-en (**18**),^15^ maoecrystal Z (**19**),^16^ rabdosin K (**20**),^17^ isorubesin C (**21**),^18^ isorubesin D (**22**),^18^ 15α-hydroxysteviol methyl ester (**23**),^19^ hyptadienic acid (**24**),^20^ amethystoidesic acid (**25**),^6^ 2α-hydroxyursolic acid (**26**),^21^ 3-epicorosolic acid (**27**),^22^ 3-*O*-α-acetyl-11-keto-β-boswellic acid (**28**),^23^ tormentic acid (**29**),^24^ euscaphic acid (**30**),^25^ myrianthic acid (**31**),^26^ 2β,3β,24-trihydroxyurs-12,20(30)-dien-28-oic acid (**32**),^27^ 2α,3α,23-trihydroxyurs-12,20(30)-dien-28-oic acid (**33**),^28^ maslinic acid (**34**),^29^ 3-*epi*maslinic acid (**35**),^30^ camaldulenic acid (**36**),^31^ hederagenin (**37**),^32^ 2α,3α,23-trihydroxy-olean-12-en-28-oic acid (**38**),^33^ hyptatic acid A (**39**),^34^ 11,12-epoxy-2,3,13-trihydroxy-γ-lactone(2α,3β,11α,12α)-oleanan-28-oic acid (**40**),^35^ α-elemolic acid (**41**),^36^ by comparison of their spectroscopic data with literature values.

**Figure 2:**
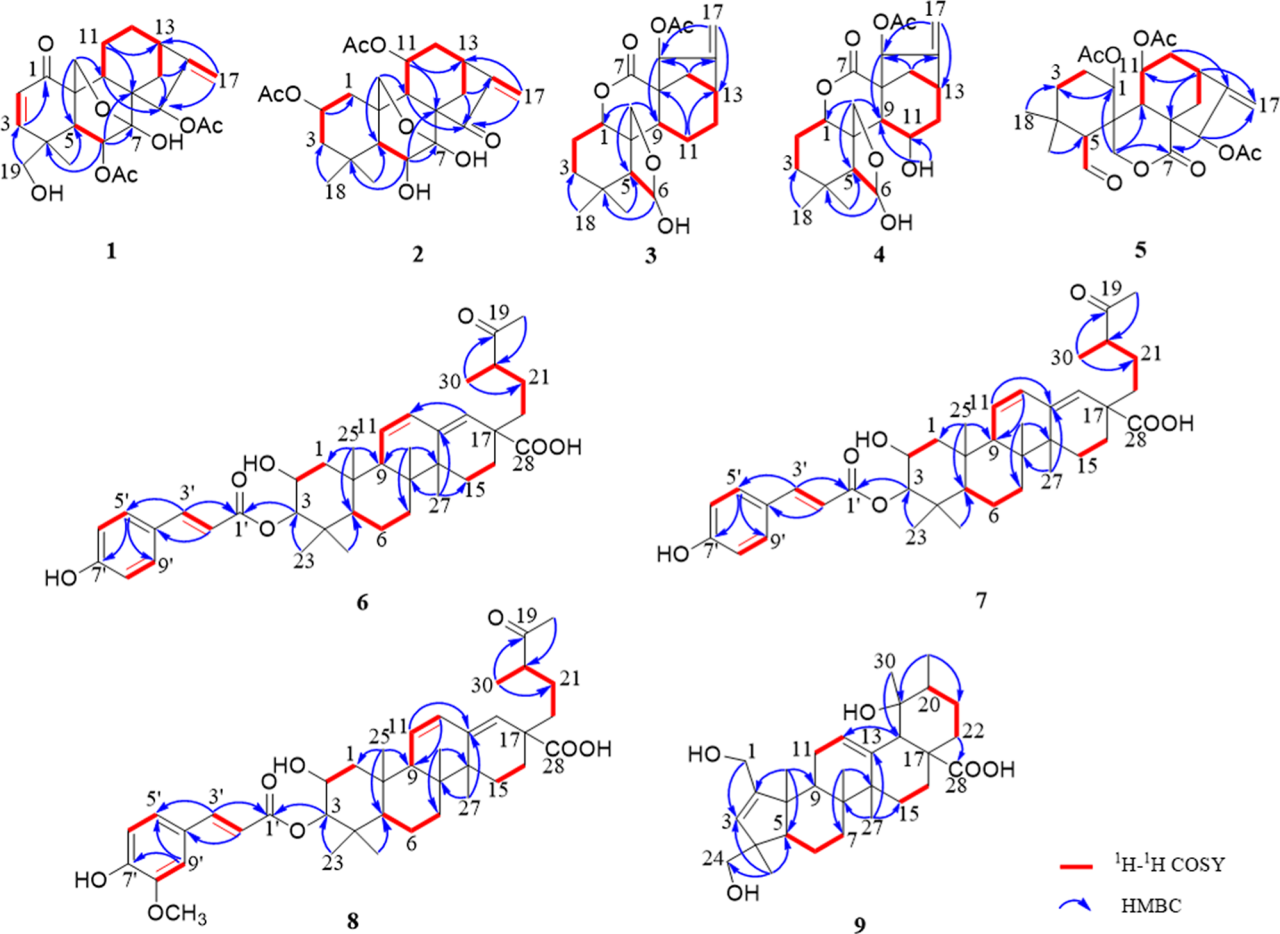
^1^H–^1^H COSY and key HMBC correlations of **1**–**9**.

**Figure 3:**
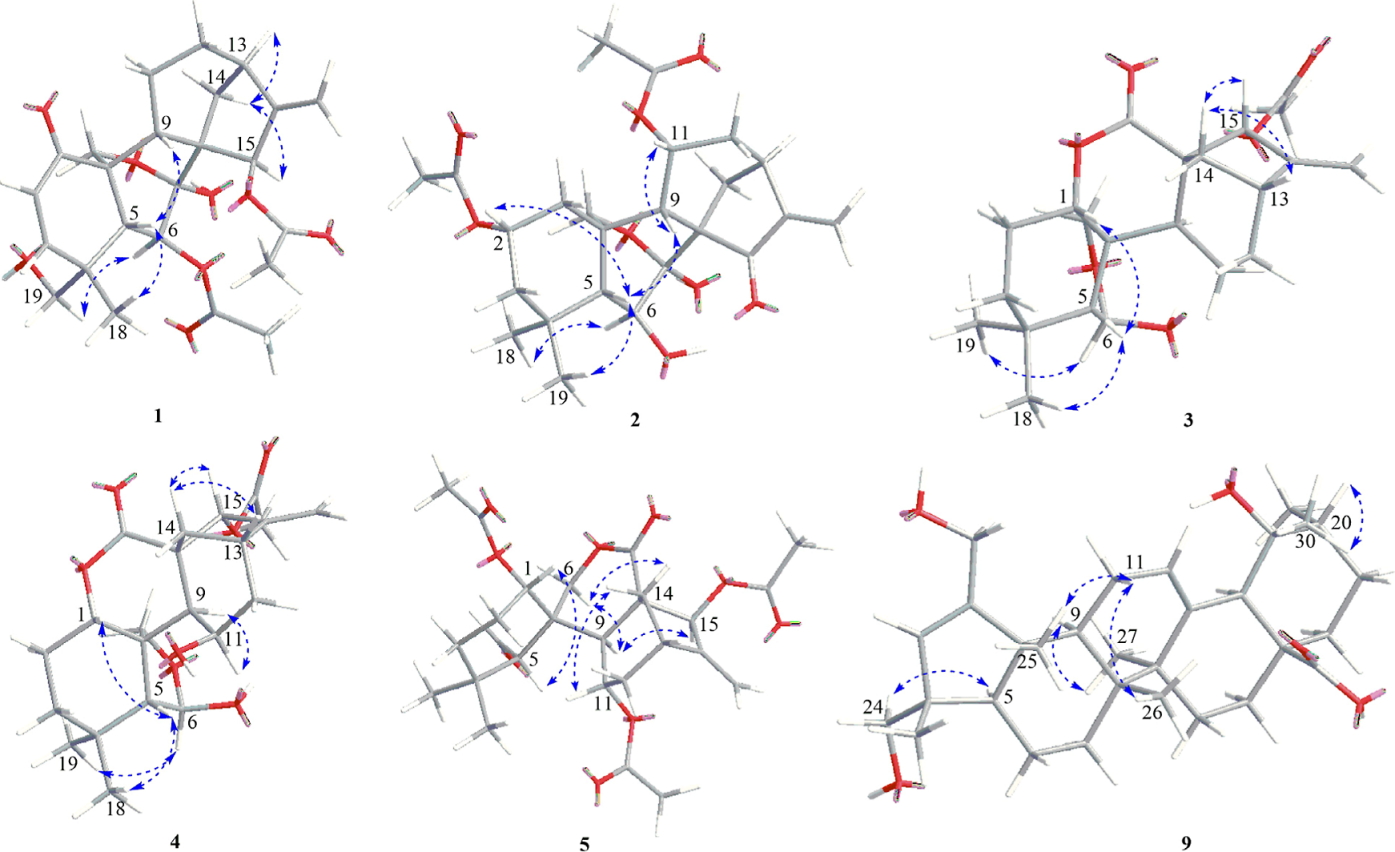
Key NOESY correlations of **1**–**5**, and **9**.

Compound **1** was obtained as a white powder, and possessed a molecular formula of C_24_H_30_O_8_, according to the HRESIMS positive ion peak at *m*/*z*: 469.1838 [M + Na] ^+^ calcd for 469.1833. The IR spectrum of **1** showed absorptions at 3433, 1728, and 1659 cm^–1^, corresponding to the hydroxy, carbonyl, and olefinic functional groups, respectively. The ^1^H NMR spectrum of **1** displayed signals for four olefinic protons at δ_H_ 6.75 (1H, d, *J* = 10.1 Hz, H-3), 6.34 (1H, d, *J* = 10.1 Hz, H-2), 5.16 (1H, m, H-17a), and 5.13 (1H, m, H-17b), six protons connected to oxygenated carbons at δ_H_ 6.29 (1H, d, *J* = 9.4 Hz, H-6), 6.22 (1H, t, *J* = 2.5 Hz, H-15), 5.11 (1H, overlap, H-20a), 4.73 (1H, dd, *J* = 9.2, 1.4 Hz, H-20b), 4.19 (1H, dd, *J* = 11.3, 5.5 Hz, H-19a), and 3.78 (1H, dd, *J* = 11.3, 4.7 Hz, H-19b) and three methyl groups at δ_H_ 2.34 (3H, s, OAc-15), 2.17 (3H, s, OAc-6), and 1.28 (3H, s, H-18) (Table 1). Analysis of its ^13^C NMR and HSQC spectra assigned 24 carbon resonances to three methyl groups (δ_C_ 25.2, 21.6, 21.3), 11 methylenes (including one olefinic at δ_C_ 109.6 and two oxygenated at δ_C_ 66.4, 66.0), six methines (including two olefinic at δ_C_ 156.6, 131.1 and two oxygenated at δ_C_ 75.7, 74.2), and eight quaternary carbons (including one olefinic at δ_C_ 159.2, one α,β-unsaturated ketone carbonyl at δ_C_ 198.1 and two carbonyl at δ_C_ 171.3, 171.1) (Table 2). The ^1^H and ^13^C NMR data of **1** were similar to those of odonicin, a 7,20-diepoxy-*ent*-kaurane diterpenoid previously isolated from *Isodon japonicus*,^37^ except for one more hydroxymethyl group (δ_C_ 66.0; δ_H_ 4.19, 3.78) at C-4 in **1** instead of a methyl group in odonicin. The HMBC correlations from H-19 to C-3 (δ_C_ 156.6) and C-5 (δ_C_ 52.5) further confirmed the presence of hydroxymethyl group at C-4 (Figure 2). The relative configuration of **1** was determined to be the same as other 7,20-diepoxy-*ent*-kaurane diterpenoids through the analysis of its NOESY spectrum (Figure 3). The additional β-orientation for Me-18 was supported by NOE correlations of H18/H-5/H-9 (Figure 3). Therefore, the structure of **1** was characterized as depicted and named serranin A.

**Table 1:** ^1^H NMR (600 MHz) Data of Compounds **1**–**5** in Pyridine-*d*_*5*_ (**δ** in ppm, *J* in Hz)

no	**1**	**2**	**3**	**4**	**5**
1		1.82, m	4.71, m	6.06, dd (10.8, 7.0)	5.01, overlap
		1.39, m			
2	6.34, d (10.1)	5.02, dd (9.7, 6.2)	1.87, m	1.91, m	1.96, m
				1.87, m	1.91, m
3	6.75, d (10.1)	1.37, m	1.38, m	1.60, dd (13.0, 4.5)	1.33, m
			1.25, m	1.37, d (13.0)	
5	3.00, d (9.4)	1.55, d (8.8)	2.22, brs	2.84, brs	3.39, d (2.9)
6	6.29, d (9.4)	4.43, d (8.8)	5.77, brs	5.85, brs	5.20, overlap
					5.11, overlap
9	2.66, dd (13.2, 5.4)	1.89, d (3.6)	3.50, m	3.46, d (4.1)	3.24, d (11.2)
11	2.06, m	5.10, t (4.2)	2.13, m	5.16, overlap	5.23, overlap
	1.59, m		1.52, m		
12	2.13, m	2.48, overlap	2.14, m	2.48, dd (14.5, 8.8)	2.84, m
	1.39, m	1.71, dd (15.7, 5.0)	1.58, m	1.99, dd (14.5, 5.6)	1.29, m
13	2.58, dd (9.0, 4.9)	3.02, dd (9.2, 4.5)	2.65, m	2.89, dd (8.8, 4.8)	2.65, dd (8.5, 5.4)
14	2.24, d (12.5)	3.15, d (12.5)	2.10, m	3.31, d (11.3)	2.41, m
	2.11, m	2.46, overlap	1.68, m	1.86, m	1.85, d (12.9)
15	6.22, t (2.5)		6.68, brs	6.87, brs	6.00, t (2.6)
17	5.16, m	6.10, s	5.07, overlap	5.10, overlap	5.18, overlap
	5.13, m	5.49, s	5.02, overlap	5.06, overlap	5.14, overlap
18	1.28, s	1.12, s	1.01, s	1.04, s	1.02, s
19	4.19, dd (11.3, 5.5)	1.32, s	1.03, s	1.08, s	1.02, s
	3.78, dd (11.3, 4.7)				
20	5.11, overlap	4.69, d (9.2)	4.18, brs	4.28, d (1.4)	10.18, d (2.9)
	4.73, dd (9.2, 1.4)	4.25, d (9.2)		4.23, d (8.7)	
OAc-1					2.10, s
OAc-2		2.13, s			
OAc-6	2.17, s				
OAc-11		2.24, s			2.15, s
OAc-15	2.34, s		2.09, s	2.11, s	2.42, s

**Table 2:** ^13^C NMR (150 MHz) Data of Compounds **1**–**5** in Pyridine-*d*_*5*_

no	**1**	**2**	**3**	**4**	**5**
1	198.1, C	25.4, CH_2_	76.7, CH	78.3, CH_2_	76.2, CH_2_
2	131.1, CH	76.7, CH	24.3, CH_2_	24.2, CH_2_	24.4, CH_2_
3	156.6, CH	38.9, CH	37.7, CH_2_	37.5, CH_2_	40.3, CH_2_
4	41.9, C	33.6, C	31.4, C	31.6, C	35.0, C
5	52.5, CH	58.4, CH	55.0, CH	55.7, CH	62.4, CH
6	74.2, CH	75.0, CH	102.3, CH	102.1, CH	66.9, CH_2_
7	96.5, C	96.0, C	175.0, C	174.6, C	173.2, C
8	52.8, C	58.8, C	52.5, C	51.9, C	51.9, C
9	44.2, CH	52.6, CH	40.3, CH	43.5, CH	41.2, CH
10	47.2, C	41.5, C	50.6, C	49.9, C	44.2, C
11	19.1, CH_2_	70.3, CH	19.2, CH_2_	65.1, CH	68.6, CH
12	32.8, CH_2_	37.8, CH_2_	32.7, CH_2_	44.8, CH_2_	40.2, CH_2_
13	36.1, CH	33.8, CH	38.1, CH	38.3, CH	35.9, CH
14	27.5, CH_2_	27.0, CH_2_	33.6, CH_2_	34.1, CH_2_	32.7, CH_2_
15	75.7, CH	208.6, C	79.8, CH	80.8, CH	82.5, CH
16	159.2, C	152.4, C	154.6, C	154.9, C	153.8, C
17	109.6, CH_2_	119.2, CH_2_	110.1, CH_2_	109.6, CH_2_	111.6, CH_2_
18	25.2, CH_3_	22.8, CH_3_	23.5, CH_3_	23.5, CH_3_	24.3, CH_3_
19	66.0, CH_2_	34.2, CH_3_	33.3, CH_3_	33.3, CH_3_	33.8, CH_3_
20	66.4, CH_2_	64.8, CH_2_	73.7, CH_2_	73.4, CH_2_	204.6, CH
OAc-1					170.2, C
					21.6, CH_2_
OAc-2		171.0, C			
		22.0, CH_3_			
OAc-6	171.3, C				
	21.6, CH_3_				
OAc-11		170.7, C			169.6, C
		22.1, CH_3_			21.6, CH_3_
OAc-15	171.1, C		170.5, C	170.2, C	170.8, C
	21.3, CH_3_		21.1, CH_3_	20.9, CH_3_	21.2, CH_3_

Serranin B (**2**) was obtained as a white powder. HRESIMS analysis yielded a quasimolecular ion at *m*/*z*: 449.2173 [M + H] ^+^, affording a molecular formula of C_24_H_32_O_8_ calcd for 449.2170. The ^1^H NMR data (Table 1) showed characteristic signals for two methyl units of an acetyl group [δ_H_ 2.24 (3H, s, OAc-11) and 2.13 (3H, s, OAc-2)], two methyls [δ_H_ 1.32 (3H, s, H-19), 1.12 (3H, s, H-18)], one oxygenated methylene [δ_H_ 4.69 (1H, d, *J* = 9.2 Hz, H-20a) and 4.25 (1H, d, *J* = 9.2 Hz, H-20b)], three protons of oxygenated methines [δ_H_ 5.10 (1H, t, *J* = 4.2 Hz, H-11), 5.02 (1H, dd, *J* = 9.7, 6.2 Hz, H-2), and 4.43 (1H, d, *J* = 8.8 Hz, H-6)], and an exocyclic double bond [δ_H_ 6.10 (1H, s, H-17a) and 5.49 (1H, s, H-17b)]. The ^13^C NMR, DEPT135, and HSQC spectra disclosed 24 carbon resonances (Table 1), containing four singlet methyl groups, six methylenes, six methines, and eight nonprotonated carbons. The above data of **2** were close to those of longikaurin E (**12**), except for an additional acetyl group signal attached at C-2 [δ_C_ 76.7; δ_H_ 5.02]. The acetyl subunit at C-2 in **2** was confirmed by MS data, the downfield chemical shift of H-2 at δ_H_ 5.02, the ^1^H–^1^H COSY correlations of H-1 (δ_H_ 1.82, 1.39)/H-2 (δ_H_ 5.02)/H-3 (δ_H_ 1.37), and the HMBC correlations from H-2 to C-1 (δ_C_ 25.4) and C-10 (δ_C_ 41.5) (Figure 2). The relative configuration of **2** was then established by analysis of the NOE interactions of H-2/H-5, indicating that they were designated as β-oriented. Detailed 2D NMR data analysis further established the structure of **2** as shown.

Serranin C (**3**) exhibited a molecular formula of C_22_H_30_O_6_ with eight indices of hydrogen deficiency indicated by HRESIMS ion at *m*/*z*: 391.2123 [M + H] ^+^ calcd 391.2115. The ^1^H NMR spectrum of **3** (Table 1) displayed characteristic resonances of three methyl singlets at δ_H_ 2.09 (3H, s, OAc-15), 1.03 (3H, s, H-19), and 1.01 (3H, s, H-18), one oxygenated methylene at δ_H_ 4.18 (2H, br s, H-20), and three oxygenated methines at δ_H_ 6.68 (1H, br s, H-15), 5.77 (1H, br s, H-6), and 4.71 (1H, m, H-1), and two exocyclic double bond protons at δ_H_ 5.07 (1H, overlap, H-17a) and 5.02 (1H, overlap, H-17b). The ^13^C NMR and DEPT spectra of **3** showed 22 carbon signals assignable to a lactonic carbonyl carbon at δ_C_ 175.0 (C-7), an ester carbonyl carbon at δ_C_ 170.5 (OAc-15), one double bond at δ_C_ 154.6 (C-16) and 110.1 (C-17), three oxygenated tertiary carbons at δ_C_ 102.3 (C-6), 79.8 (C-15), 76.7 (C-1), and one oxygenated secondary carbons at δ_C_ 73.7 (C-20), and 14 sp^3^ carbons at δ_C_ 19.2–55.0 including three methyls, five methylenes, three methines, and three nonprotonated carbons. The NMR data of **3** (Tables 1 and 2) closely resembled those of 6α,15β-dihydroxy-6,20-epoxy-emein-16-en (**18**), confirming the same skeleton of enmein-type diterpenoids. However, notable differences between the two compounds were the existence of additional acetyl resonances [δ_C_ 170.5, 21.1; δ_H_ 2.09] in **3** and the downfield shift of the oxygenated methine (H-15) from δ_H_ 5.52 (1H, t, *J* = 2.7 Hz) in **18** to δ_H_ 6.68 (1H, br s) in **3**. NOESY correlations shown in Figure 3 indicated the relative configuration of **3** was the same as those of **18**. Thus, compound **3** was structurally characterized as drawn.

Compound **4** possesses a molecular formula C_22_H_30_O_7_, one more oxygen than that of **3**. Detailed comparison of NMR data for **4** and **3** (Table 1) revealed that they are enmein-type diterpenoids with an acetyl group at C-15, except for the presence of the oxygenated methine signals [δ_C_ 65.1, C-11; δ_H_ 5.16 (1H, overlap, H-11)] in **4**. The ^1^H–^1^H COSY correlations (Figure 2) of H-9/H-11/H-12 and the HMBC correlation (Figure 2) from HO-11 (δ_H_ 6.58) to C-9 and C-11 further supported that the hydroxy group was linked at C-11. The α-orientation of H-11 and the β-orientation of H-15 were evidenced by NOE correlations of H-9/H-11, and H-13/H-14a/H-15, respectively. The structure of **4** was established accordingly and named serranin D.

Serranin E (**5**) has a molecular formula of C_26_H_34_O_9_ with 10 degrees of unsaturation. The ^1^H NMR data (Table 1) for **5** displayed an aldehyde proton at δ_H_ 10.18 (1H, d, *J* = 2.9 Hz, H-20), an exocyclic double bond at δ_H_ 5.18 (1H, overlap, H-17a) and 5.14 (1H, overlap, H-17b), one oxygenated methylene at δ_H_ 5.20 (1H, overlap, H-6a) and 5.11 (1H, overlap, H-6b), four oxygenated methines at δ_H_ 6.00 (1H, t, *J* = 2.6 Hz, H-15), 5.23 (1H, overlap, H-11), 5.01 (1H, overlap, H-1), and 3.39 (1H, d, *J* = 2.9 Hz, H-5), and five methyl singlets at δ_H_ 2.42 (3H, s, OAc-15), 2.15 (3H, s, OAc-11), 2.10 (3H, s, OAc-1), and 1.02 (6H, s, H-18 and H-19). The ^13^C NMR and HSQC spectra of **5** exhibited 26 carbons including five methyls, four methylenes, two methines, one oxygenated secondary carbon, three oxygenated tertiary carbons, three quaternary carbons, two olefinic carbons, a lactone carbonyl carbon, and an aldehyde carbonyl carbon. A comparison of the NMR data of **5** (Tables 1 and 2) with those of isorubesin D (**22**) revealed their overall similarity, which indicated that compound **5** had a 6,7-*seco*-7,20-lactone type-*ent*-kauran skeleton. The hydroxyl group at C-11 in **22** was deduced to be replaced by an acetyl group in **5**, supported by the presence of the acetyl resonance signal [δ_C_ 169.6, 21.6; δ_H_ 2.15], the downfield shift of the oxygenated methylene proton at C-11 from δ_H_ 4.35 (1H, m) in **22** to δ_H_ 5.23 (1H, overlap) in **5**, and the COSY correlations of H-9/H-11/H-12. The relative configuration of **5** could be determined via analysis of its NOESY spectrum (Figure 3). The structure of **5** was accordingly assigned as shown.

Serratic acids A (**6**) and B (**7**) gave the same molecular formula of C_39_H_52_O_7_, as indicated by their HRESIMS ions at 633.3780 [M + H]^+^ calcd for C_39_H_53_O_7_, 633.3786 and 633.3792 [M + H]^+^ calcd for C_39_H_53_O_7_, 633.3786, respectively. The ^1^H and ^13^C NMR data (Tables 3 and 4) of **6** together with its HSQC data suggested the presence of six singlet methyl groups [δ_H_ 2.11, 1.03, 1.02, 0.94, 0.92, 0.85; δ_C_ 28.9, 28.4, 20.5,19.5, 17.9, 17.1], one primary methyl group [δ_H_ 1.05 (d, *J* = 7.0 Hz, H-30)/δ_C_ 16.6, C-30], seven methylenes, 14 methines, and 11 nonprotonated carbons. The above NMR data of **6** showed 39 carbon signals attributable to a triterpenoid skeleton and a *p*-coumaroyl fragment. The triterpenoid unit of **6** is close to swinhoeic acid, a 18,19-*seco*-ursane triterpenoid isolated from *Rubus swinhoei*,^38^ based on the 2D NMR analysis (Figures 2 and 3). The appearance of a (*Z*)-*p*-coumaroyl fragment was confirmed by the chemical shift and coupling constant of double bonds at δ_H_ 6.94 (1H, d, *J* = 12.8 Hz, H-3′) and 6.11 (1H, d, *J* = 12.8 Hz, H-2′), the correlations of H-2’/H-3′, H-5′/H-6′, and H-8’/H-9′ observed in the ^1^H–^1^H COSY spectrum and the HMBC signals from H-2′ to C-4′, from H-3′ to C-1′, C-5′, and from aromatic proton H-5′ to C-7′, C-9’. The (*Z*)-*p*-coumaroyl moiety was connected at C-3 position on the basis of the HMBC correlations between H-3 [δ_H_ 5.20 (1H, overlap)] and the ester carbonyl carbon [δ_C_ 167.5, C-1’] of the *p*-coumaroyl group. The NMR data of **7** resembled those of compound **6**, except for the difference in the chemical shift and coupling constant of double bonds in **7** [8.00 (1H, d, *J* = 15.9 Hz, H-3′), 6.69 (1H, d, *J* = 15.9 Hz, H-2′)], suggesting the presence of (*E*)-*p*-coumaroyl moiety in **7**. Therefore, compound **7** is an isomer of **6** and established as 3-*O*-(*E*)-*p*-coumaroyl swinhoeic acid.

**Table 3:** ^1^H NMR (600 MHz) Data of Compounds **6**–**9** in Pyridine-*d*_*5*_ (**δ** in ppm, *J* in Hz)

no	**6**	**7**	**8**	**9**
1	2.55, m	2.57, m	2.56, m	4.60, d (15.9)
	1.41, m	1.41, m	1.41, m	4.48, d (15.9)
2	4.30, td (10.7, 4.4)	4.35, td (10.7, 4.5)	4.36, overlap	
3	5.20, overlap	5.25, overlap	5.27, overlap	6.07, s
5	1.09, m	1.10, m	1.09, m	2.02, m
6	1.50, m	1.52, m	1.51, m	1.67, m
	1.32, m	1.34, m	1.34, m	1.57, m
7	1.33, m	1.34, m	1.33, m	1.68, m
	1.28, m	1.31, m	1.29, m	1.39, m
9	2.12, m	2.14, m	2.13, m	2.58, dd (10.8, 6.3)
11	6.16, dd (10.1, 3.0)	6.18, dd (10.1, 3,0)	6.18, dd (10.0, 3.0)	2.39, m
				2.06, m
12	5.71, d, (10.1)	5.73, d (10.1)	5.74, d (10.0)	5.58, overlap
15	2.20, m	2.20, m	2.18, m	1.31, m
	1.23, m	1.23, m	1.24, m	1.25, m
16	2.61, m	2.60, m	2.58, m	2.06, m
	1.61, m	1.62, m	1.62, m	2.02, m
18	5.89, brs	5.89, brs	5.87, brs	3.02, brs
20	2.53, m	2.54, m	2.54, m	1.47, m
21	2.02, m	2.02, m	2.01, m	2.03, m
	1.67, m	1.65, m	1.65, m	1.32, m
22	2.02, m	2.03, m	2.01, m	2.12, m
	1.71, m	1.72, m	1.72, m	2.05, m
23	0.92, s	1.00, s	0.94, s	1.20, s
24	1.02, s	1.04, s	1.05, s	3.81, d (10.1)
				3.70, d (10.1)
25	0.94, s	0.99, s	0.99, s	1.23, s
26	0.85, s	0.85, s	0.85, s	1.15, s
27	1.03, s	1.06, s	1.06, s	1.64, s
29	2.11, s	2.11, s	2.11, s	1.09, d (6.6)
30	1.05, d (7.0)	1.06, d (7.0)	1.06, d (7.0)	1.41, s
2′	6.11, d (12.8)	6.69, d (15.9)	6.76, d (15.8)	
3′	6.94, d (12.8)	8.00, d (15.9)	8.03, d (15.8)	
5′	8.13, d (8.7)	7.56, overlap	7.17, dd (8.2, 2.0)	
6′	7.16, d (8.7)	7.19, overlap	7.21, overlap	
8′	7.16, d (8.7)	7.19, overlap		
9′	8.13, d (8.7)	7.56, overlap	7.30, brs	
OCH_3_			3.79, s	

**Table 4:** ^13^C NMR (150 MHz) Data of Compounds **6**–**9** in Pyridine-*d*_*5*_

no	**6**	**7**	**8**	**9**
1	48.3, CH_2_	48.2, CH_2_	48.3, CH_2_	61.3, CH_2_
2	66.5, CH	66.6, CH	66.8, CH	158.4, C
3	85.1, CH	85.2, CH	85.2, CH	130.7, CH
4	40.0, C	40.1, C	40.1, C	48.7, C
5	55.2, CH	55.2, CH	55.2, CH	58.5, CH
6	18.7, CH_2_	18.7, CH_2_	18.7, CH_2_	18.9, CH_2_
7	32.5, CH_2_	32.6, CH_2_	32.6, CH_2_	34.6, CH_2_
8	41.1, C	41.1, C	41.1, C	42.4, C
9	54.7, CH	54.7, CH	54.8, CH	43.8, CH
10	38.3, C	38.3, C	38.3, C	51.4, C
11	131.1, CH	130.9, CH	131.0, CH	27.4, CH_2_
12	127.0, CH	127.3, CH	127.4, CH	128.4, CH
13	142.2, C	142.4, C	142.6, C	140.4, C
14	41.8, C	41.8, C	41.8, C	42.7, C
15	27.0, CH_2_	27.0, CH_2_	26.9, CH_2_	30.0, CH_2_
16	27.8, CH_2_	27.8, CH_2_	27.7, CH_2_	26.6, CH_2_
17	49.9, C	49.6, C	49.9, C	48.6, C
18	130.1, CH	129.7, CH	129.5, CH	55.1, CH
19	212.0, C	212.0, C	211.9, C	72.9, C
20	47.8, CH	47.7, CH	47.7, CH	42.6, CH
21	28.6, CH_2_	28.6, CH_2_	28.5, CH_2_	27.2, CH_2_
22	39.4, CH_2_	39.7, CH_2_	39.3, CH_2_	38.8, CH_2_
23	17.9, CH_3_	18.0, CH_3_	18.0, CH_3_	17.6, CH_3_
24	28.9, CH_3_	28.9, CH_3_	28.9, CH_3_	71.8, CH_2_
25	19.5, CH_3_	19.6, CH_3_	19.6, CH_3_	19.9, CH_3_
26	17.1, CH_3_	17.1, CH_3_	17.0, CH_3_	19.3, CH_3_
27	20.5, CH_3_	20.5, CH_3_	20.5, CH_3_	25.7, CH_3_
28	178.7, C	178.4, C	178.4, C	170.7, C
29	28.4, CH_3_	28.4, CH_3_	28.4, CH_3_	17.0, CH_3_
30	16.6, CH_3_	16.6, CH_3_	16.6, CH_3_	27.4, CH_3_
1′	167.5, C	168.3, C	168.4, C	
2′	117.3, CH	116.3, CH	116.3, CH	
3′	144.0, CH	145.2, CH	145.3, CH	
4′	127.1, C	126.5, C	127.1, C	
5′	134.0, CH	130.9, CH	123.8, CH	
6′	116.2, CH	117.1, CH	117.1, CH	
7′	160.8, C	161.6, C	151.3, C	
8′	116.2, CH	117.1, CH	149.3, C	
9′	134.0, CH	130.9, CH	111.7, CH	
OCH_3_			56.2, CH_3_	

Serratic acid C (**8**) was isolated as a white powder. Its HRESIMS spectrum showed a [M + H] ^+^ ion at *m/z*: 663.3896 calcd for 663.3891, indicative of a molecular formula of C_40_H_54_O_8_. A comparison of the NMR data of **8** with those of **7** suggested that they were closely related to each other. An additional methoxy group [δ_C_ 56.2, δ_H_ 3.79 (3H, s)] was observed in **8**, which indicated a feruloyl group in **8** replacing the (*E*)-*p*-coumaroyl group of **7**. Detailed 2D NMR data analysis (Figures 2 and 3) confirmed the structure of **8** as 3-*O*-(*E*)-feruloyl swinhoeic acid.

Compound **9** (serratic acid D) yielded a molecular formula of C_30_H_46_O_5_, as confirmed by the HRESIMS at *m/z*: 504.3682 [M + NH_4_] ^+^ calcd for 504.3684. The ^13^C NMR (Table 4) data of **9** showed 30 carbon signals attributable to a triterpenoid skeleton. Comparison of the NMR data of **9** with those of **24** indicated similar structures for these molecules. Differences were noted from the presence of a hydroxymethyl [δ_C_ 71.8, C-24, δ_H_ 3.81 (1H, d, *J* = 10.1 Hz, H-24a) and 3.70 (1H, d, *J* = 10.1 Hz, H-24b)] in **9** and the absence of a methyl group resonance from **24**. The HMBC correlations of Me-23 [δ_H_ 1.20 (3H, s)] with the oxygenated methylene carbon signals (C-24) imply that the hydroxymethyl at C-4 in **9**. NOESY correlations were used to assign the relative configuration of the **9**. NOE correlation between H-24b and H-5 (δ_H_ 2.02, m) indicated the α*-*orientation of the hydroxymethyl group.

*Ent*-kaurane diterpenoids from *Isodon* genus are well-known for their cytotoxic activity. Therefore, to evaluate the antiproliferative effects of these terpenoids isolated from the aerial parts of *I. serra*, the B16–F10 mouse melanoma cells, A375 human melanoma cells, A549 lung tumor cells, and MDA-MB-231 breast cancer cells were treated with varying concentrations of isolated compounds, and general chemotherapeutic drug paclitaxel was referenced. The preliminary results showed that antiproliferative effects of diterpenoids were higher than those of triterpenoids at 20 μM (data not shown). The IC_50_ values of selected compounds were then detected. As shown in Table 5, diterpenoids with an unsaturated ketone (**2**, **10**–**13**) displayed moderate cytotoxic activity against four tumor cell lines with IC_50_ values ranging from ranging from 3.29 to 13.39 μM. The malignant melanoma B16–F10 cell line was more sensitive to **6**, **14**, **16,** and **17** than the A375 cell line. Compound **6,** a new triterpenoid, implied moderate activity against B16–F10 melanoma cells and A549 lung cancer cells with IC_50_ values of 10.42 ± 0.03 and 8.80 ± 0.10 μM, respectively. Moreover, **19** and **21 (**spiro-lactone diterpenoids) exhibited selective inhibitory effects on MDA-MB-231 breast cancer cells. The results indicated that α,β-unsaturated cyclopentanone ring D of diterpenoids (**2**, **10**–**14**) is considered as an active site with potential anticancer properties.

**Table 5:** IC_50_ Values (μM) of Terpenoids from the Aerial Parts of *I. serra* Against Four Tumor Cells^a^

compounds	B16–F10	A375	A549	MDA-MB-231
**2**	8.24 ± 0.01	8.97 ± 0.20	6.67 ± 0.07	8.10 ± 0.14
**6**	10.42 ± 0.03	>20	8.80 + 0.10	>20
**10**	5.33 ± 0.10	5.27 ± 0.10	3.92 ± 0.14	4.20 ± 0.09
**11**	12.93 ± 0.19	13.39 ± 0.14	10.38 ± 0.18	12.08 ± 0.18
**12**	5.96 ± 0.12	8.13 ± 0.10	5.23 ± 0.06	7.15 ± 0.10
**13**	4.64 ± 0.04	3.58 ± 0.09	3.29 ± 0.08	3.38 ± 0.09
**14**	8.14 ± 0.41	>20	12.46 ± 0.21	>20
**16**	11.00 ± 0.40	>20	12.49 ± 0.21	>20
**17**	18.36 ± 0.48	>20	13.34 ± 0.09	>20
**19**	>20	>20	>20	10.63 ± 0.25
**21**	>20	>20	>20	15.10 ± 0.23
**26**	>20	15.24 ± 0.22	11.45 ± 0.22	>20
paclitaxel	0.28 ± 0.002	0.17 ± 0.001	0.12 ± 0.003	0.11 ± 0.001

a Data are presented as the mean ± SEM of three independent experiments. Paclitaxel: positive control.

**Figure 4:**
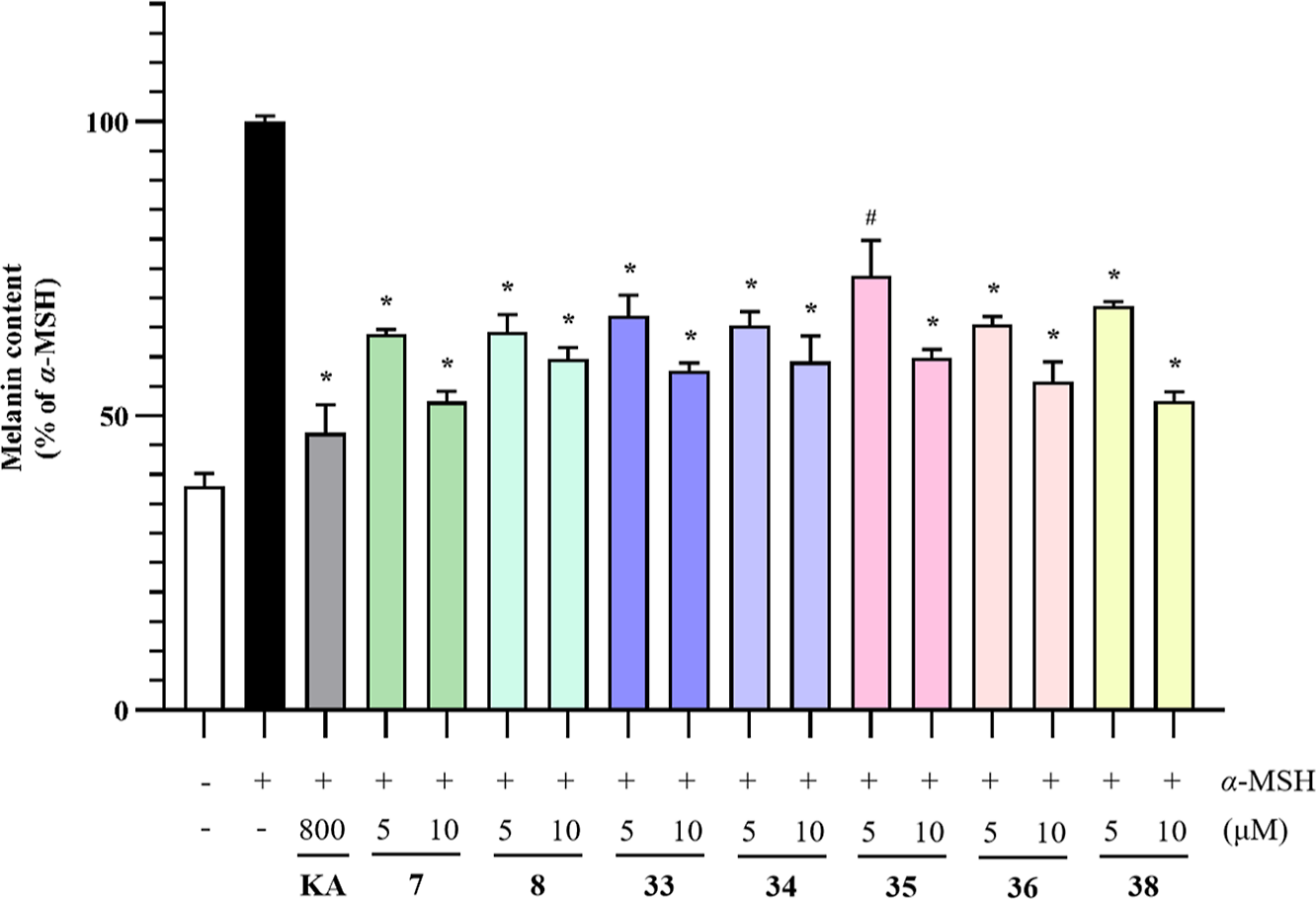
Effects of **7**, **8**, **33**–**36**, and **38** on melanogenesis in B16–F10 cells. Each column represents the mean ± SD of three independent experiments. ^#^*P* < 0.05 and *P < 0.01 indicate significant difference compared to the α-MSH group.

The effects of isolated compounds on the α-MSH-induced melanin production of the B16–F10 cells were also assessed. As shown in Figure 4, treatment with noncytotoxic concentrations of (5 and 10 μM) compounds **7**–**8**, **33**–**36**, and **38** significantly reduced the α-MSH (10 nM) induced melanin contents in the B16–F10 cells. High concentrations of **7** and **38** (10 μM) had melanin production inhibition rates of 47.5% and 47.4%, respectively, which is close to that of positive control, kojic acid (KA, 800 μM). Structurally, compounds **7**–**8** are ursane-type triterpenoid *p*-coumaric or ferulic esters, while **34**–**36**, and **38** are oleanane-type triterpenoids with 2,3-hydroxy and 28-carboxyl acid groups, indicating these groups may be related to the melanin production inhibitory activity of triterpenoids.

## Conclusions

In conclusion, in our continuous chemical investigation on the aerial parts of *I. serra*, five undescribed diterpenoids, serranins A–E (**1**–**5**), and four novel triterpenoids, serratic acids A–D (**6**–**9**), along with 32 known terpenoids (**10**–**41**) were discovered. Serratic acids A–C (**6**–**8**) represent a new type of triterpenoid ester possessing a 18,19-*seco* ring E ursane skeleton and the *p*-coumaric or ferulic triterpene esters moiety. Serranins A–E (**1**–**5**) are new *ent*-kaurane, enmein, and spiro-lactone-type diterpenoids, which further enriched the plant’s diterpene profile. An isosteviol-type diterpenoid (**23**) and two triterpenoids (**28** and **40**) are new natural products and their spectroscopic data are reported for the first time (Supporting Information, Tables S1–S3). Moreover, α-elemolic acid (**41**) is the first report of tirucallane-type triterpenoid identified from the plant genus *Isodon.* Except for compounds **11**, **13**, **16**, **17**, and **20**, the remaining 23 known compounds were first isolated from this species.

Bioassay results showed that diterpenoids with an unsaturated ketone (**2**, **10**–**13**) displayed moderate cytotoxic activity against four tumor cell lines with IC_50_ values ranging from 3.29 to 13.39 μM. Seven triterpenoids (**7**, **8**, **33**–**36**, and **38**) notably diminished the α-MSH-induced melanin production in B16–F10 cells treated with a concentration of 10 μM, slightly lower than the effects of the positive drug (KA, 800 μM). This work greatly enriched the chemical library of *I. serra* and offered a valuable structural template for the development of antitumor-leading compounds and effective natural whitening products.

## Experimental Procedures

### General Experimental Procedures

Optical rotations were acquired on an Autopol I automatic polarimeter at room temperature (Rudolph Research Analytical). The IR spectra were recorded by a IRAffinity-1S spectrometer (Shimadzu, Japan). The UV and CD spectra were measured in a J-1500 circular dichroism spectrophotometer (JASCO, Japan). HRESIMS spectra were performed on an Agilent 6230 TOF mass spectrometer (Agilent, USA). Medium pressure liquid chromatography (MPLC) was carried out on a Buchi C-620 system (Buchi, Switzerland) equipped with a C-635 UV detector and a Siliabond C_18_ column (ODS gel, 40–63 μm, 26 × 460 mm) or an MCI column (Unips 40–300 μm, 36 × 460 mm). Semipreparative HPLC purifications were conducted on A ThermoFisher Ultimate 3000 RSLC system (Thermo, USA) accompanied by a 3000 Diode Array detector (Thermo, USA), using an XSelect CSH fluoro-phenyl OBD column (5 μm, 10 × 250 mm, Waters, USA) and a Vision HT C18 HL column (5 μm, 10 × 250 mm, Grace, Germany) at a flow rate of 3 mL/min and UV detection at 210 and 254 nm. 1D and 2D NMR spectra were obtained using Bruker Avance 600 MHz spectrometer (Bruker, Germany) with TMS as an internal standard. Silica gel (200–300 mesh; Qingdao Haiyang Chemical Co. Ltd., Qingdao, China) was used for column chromatography. Precoated silica gel GF254 plates (Merck, Darmstadt, Germany) were used for TLC. TLC spots were visualized by spraying with H_2_SO_4_–EtOH (1:9, v/v) followed by heating.

### Plant Material

The aerial parts of *I. serra* (Maxim.) Kudô were purchased from Bozhou Herbal Medicine Market, Anhui Province, China, and were authenticated by Dr. G.-Y Zhu of Macau University of Science and Technology. A voucher specimen (IS-2023–01) was deposited in the State Key Laboratory of Quality Research in Chinese Medicine, Macau University of Science and Technology.

### Extraction and Isolation

The dried aerial parts of *I. serra* (15.0 kg) were powdered and extracted with 80% ethanol under reflux three times, and the EtOH extract was concentrated to about a 10 L water suspension and then partitioned successively with PE, EtOAc, and *n*-BuOH to yield PE (214.9 g), EtOAc (157.2 g), and *n*-BuOH (146.7 g) fractions. Subsequently, the PE extract was separated by silica gel CC eluting with PE–EtOAc–MeOH (1:0:0 to 1:1:1, v/v/v) to obtain 22 fractions (Fr.P.A–P.V). Fr.P.R (17.1 g) was chromatographed on a C_18_ MPLC column eluted with a gradient of MeCN–H_2_O (40–100%, v/v) to give compounds **24** (3.0 mg, *t*_R_ = 185.0 min), **28** (25.4 mg, *t*_R_ = 215.0 min), and **41** (20.0 mg, *t*_R_ = 245.0 min). Fr.P.V (44.0 g) was chromatographed by MPLC with a gradient elution of MeCN–H_2_O (from 40:60 to 100:0, v/v) to provide **39** (62.0 mg, *t*_R_ = 65.0 min) and the remaining 38 subfractions. Fr.P.V8 was subjected to semipreparative HPLC eluted with MeOH–H_2_O (63:37) and MeCN–H_2_O (36:64) to yield **1** (1.0 mg, *t*_R_ = 19.7 min). Fr.P.V9 was fractionated over a reversed-phase column (63% MeOH in H_2_O) to give subfraction Fr.P.V9–6 (22.5 mg, *t*_R_ = 11.9 min), **2** (14.8 mg, *t*_R_ = 13.9 min), and **17** (23.8 mg, *t*_R_ = 22.1 min). Compounds **15** (10.5 mg, *t*_R_ = 28.0 min) and **10** (7.2 mg, *t*_R_ = 34.9 min) were further obtained from subfraction P.V9–6 over HPLC (58% MeOH in H_2_O). Fr.P.V10 was subjected to reversed-phase HPLC using 63% MeOH in H_2_O as eluent to obtain Fr.P.V10–2 (28.2 mg, *t*_R_ = 9.9 min), Fr. P.V10–3 (4.5 mg, *t*_R_ = 10.9 min), **14** (8.9 mg, *t*_R_ = 12.1 min), **12** (5.0 mg, *t*_R_ = 13.9 min), and Fr. P.V10–6 (3.0 mg, *t*_R_ = 15.5 min). Compounds **16** (1.6 mg, *t*_R_ = 14.5 min), **18** (12.8 mg, *t*_R_ = 15.1 min), **4** (6.3 mg, *t*_R_ = 18.5 min), **19** (1.1 mg, *t*_R_ = 21.1 min) and **21** (1.4 mg, *t*_R_ = 24.8 min) were isolated from Fr. P.V10–2 by semipreparative HPLC (MeCN–H_2_O, 36:64). Fr. P.V10–3 was subjected to HPLC separation (MeCN–H_2_O, 34:66) to give **23** (0.5 mg, *t*_R_ = 23.3 min) and **22** (1.0 mg, *t*_R_ = 27.8 min). Fraction P.V10–6 was purified by semipreparative C_18_ HPLC eluting with an isocratic mixture of 43% MeCN to obtain **13** (1.1 mg, *t*_R_ = 22.2 min). Separation of Fr.P.V12 by reversed-phase semipreparative HPLC (61% MeOH in H_2_O) afforded Fr.P.V12–6 (50.0 mg, *t*_R_ = 12.5 min). Fr.P.V12–6 was further purified by reversed-phase HPLC (MeCN–H_2_O, 33:67) to provide **3** (2.8 mg, *t*_R_ = 31.1 min) and **20** (19.8 mg, *t*_R_ = 34.2 min). Compound **5** (7.0 mg) was obtained from Fr.P.V13 by repeated recrystallization using MeOH. Fraction P.V19 was repeatedly chromatographed on HPLC (MeCN–H_2_O, 65:35 and 80% MeOH in H_2_O) to obtain **25** (1.1 mg, *t*_R_ = 15.0 min).

The EtOAc extract (157.2 g) was chromatographed on a silica gel column with a gradient PE–EtOAc–MeOH (1:0:1 to 1:1:1, v/v) to yield the 24 fractions (Fr.E.A-E.X). Fr.E.O (23.0 g) was first isolated by MPLC to yield Fr.E.O24 (746.9 mg) and Fr.E.O26. Fr.E.O24 was further purified by HPLC eluted with MeCN–H_2_O (60:40) and MeOH–H_2_O (67:33) to provide **40** (1.1 mg, *t*_R_ = 34.2 min), **37** (3.4 mg, *t*_R_ = 47.5 min), **36** (8.3 mg, *t*_R_ = 51.9 min) and **34** (15.8 mg, *t*_R_ = 54.2 min). Fr.E.O26 was fractionated by using a semipreparative C_18_ column to afford Fr.E.O26–1–11 (CH_3_CN–H_2_O, 67:33). Compounds **6** (1.1 mg, *t*_R_ = 33.2 min), **7** (1.0 mg, *t*_R_ = 44.3 min) and **8** (0.6 mg, *t*_R_ = 51.1 min) were purified from Fr.E.O26–5 using semipreparative HPLC (MeOH–H_2_O, 65:35). Fr.E.P (17.1 g) was separated on a MPLC column (MeCN–H_2_O, 20:80 to 100:1) to give 24 subfractions, and the 20th fraction was purified by HPLC (60% MeCN in H_2_O) to yield Fr.E.P20–4 (21.1 mg, *t*_R_ = 10.8 min), **35** (13.2 mg, *t*_R_ = 22.5 min), **27** (22.5 mg, *t*_R_ = 24.2 min), and **26** (4.5 mg, *t*_R_ = 28.1 min). Fr.E.P20–4 was further purified by semipreparative HPLC eluted with MeOH–H_2_O (58:42) to yield **30** (19.3 mg, *t*_R_ = 39.0 min) and **38** (13.8 mg, *t*_R_ = 57.3 min). Fr.E.Q (24.5 g) was separated by an MPLC column (MeCN–H_2_O, 20:80 to 100:0) to afford 44 fractions (Fr.E.Q1–E.Q44). Compound **33** (3.6 mg, *t*_R_ = 52.1 min) was afforded from the subfraction Fr.E.Q26–3 (136.8 mg) by HPLC purification using 58% MeOH as an eluent. Compound **11** (2.9 mg, *t*_R_ = 45.0 min) was purified from Fr.E.R (10.2 g) with an MPLC column (CH_3_CN–H_2_O, 20:80 to 100:0, v/v). Fraction E.S (22.3 g) was subjected to MPLC column chromatography using a solvent mixture of MeCN–H_2_O (from 20:80 to 100:0, v/v) and repeatedly purified by HPLC (46% MeCN in H_2_O) to yield subfractions E.S15–1–7. Fr.E.S15–6 (60.7 mg) was purified by semipreparative HPLC (MeOH–H_2_O, 70:30) to afford **31** (2.2 mg, *t*_R_ = 10.9 min) and **32** (1.2 mg, *t*_R_ = 15.8 min). Fr.E.S16 was fractionated and purified by reversed-phase HPLC (MeOH–H_2_O, 58:42) to give **9** (0.5 mg, *t*_R_ = 28.7 min). Compound **29** (12.9 mg, *t*_R_ = 36.0 min) was isolated from Fr.E.S19 by HPLC (MeOH–H_2_O, 65:35).

### Serranin A (**1**)

White powder; [α]^25^_D_ – 128.4 (*c* 0.5, MeOH); UV (MeOH) λ_max_ (log ε) 226.8 (0.41), 195 (0.97) nm; IR (KBr) ν_max_ 3433, 1728, 1659, 1373, 1242, 1057 cm^–1^; ^1^H (pyridine-*d*_5_, 600 MHz) and ^13^C (pyridine-*d*_5_, 150 MHz) NMR data, see Tables 1 and 2; HRESIMS *m*/*z:* 469.1838 [M + Na] ^+^ calcd for C_24_H_30_O_8_Na, 469.1833.

### Serranin B (**2**)

White powder; [α]^25^_D_ – 21.7 (*c* 0.5, MeOH); UV (MeOH) λ_max_ (log ε) 232.7 (0.25), 195 (0.55) nm; IR (KBr) ν_max_ 3402, 1713, 1366, 1250, 1049 cm^–1^; ^1^H (pyridine-*d*_5_, 600 MHz) and ^13^C (pyridine-*d*_5_, 150 MHz) NMR data, see Tables 1 and 2; HRESIMS *m*/*z*: 449.2173 [M + H] ^+^ calcd for C_24_H_33_O_8_, 449.2170.

### Serranin C (**3**)

White powder; [α]^25^_D_ – 136.5 (*c* 0.5, MeOH); UV (MeOH) λ_max_ (log ε) 195 (0.70) nm; IR (KBr) ν_max_ 3410, 1736, 1366, 1234, 1049, 1003, 903 cm^–1^; ^1^H (pyridine-*d*_5_, 600 MHz) and ^13^C (pyridine-*d*_5_, 150 MHz) NMR data, see Tables 1 and 2; HRESIMS *m*/*z*: 391.2123 [M + H] ^+^ calcd for C_22_H_31_O_6_, 391.2115.

### Serranin D (**4**)

White powder; [α]^25^_D_ – 115.4(*c* 0.5, MeOH); UV (MeOH) λ_max_ (log ε) 195 (0.81) nm; IR (KBr) ν_max_ 3433, 1728, 1373, 1234, 1049 cm^–1^; ^1^H (pyridine-*d*_5_, 600 MHz) and ^13^C (pyridine-*d*_5_, 150 MHz) NMR data, see Tables 1 and 2; HRESIMS *m*/*z*: 407.2071 [M + H] ^+^ calcd for C_22_H_31_O_7_, 407.2064.

### Serranin E (**5**)

White powder; [α]^25^_D_ + 44.5 (*c* 0.5, MeOH); UV (MeOH) λ_max_ (log ε) 195 (0.64) nm; IR (KBr) ν_max_ 3456, 1744, 1373, 1227, 1042 cm^–1^; ^1^H (pyridine-*d*_5_, 600 MHz) and ^13^C (pyridine-*d*_5_, 150 MHz) NMR data, see Tables 1 and 2; HRESIMS *m*/*z*: 491.2287 [M + H] ^+^ calcd for C_26_H_35_O_9_, 491.2276.

### Serratic Acid A (**6**)

White powder; [α]^25^_D_ – 56.3 (*c* 0.5, MeOH); UV (MeOH) λ_max_ (log ε) 310 (0.51), 235.8 (0.87) nm; IR (KBr) ν_max_ 3410, 2870, 1705, 1605, 1450, 1366, 1281, 1165 cm^–1^; ^1^H (pyridine-*d*_5_, 600 MHz) and ^13^C (pyridine-*d*_5_, 150 MHz) NMR data, see Tables 3 and 4; HRESIMS *m*/*z*: 633.3780 [M + H] ^+^ calcd for C_39_H_53_O_7_, 633.3786.

### Serratic Acid B (**7**)

White powder; [α]^25^_D_ – 35.1 (*c* 0.5, MeOH); UV (MeOH) λ_max_ (log ε) 311.8 (0.38), 232.5 (0.51) nm; IR (KBr) ν_max_ 3380, 2870, 1713, 1605, 1450, 1366, 1273, 1165, 1049 cm^–1^; ^1^H (pyridine-*d*_5_, 600 MHz) and ^13^C (pyridine-*d*_5_, 150 MHz) NMR data, see Tables 3 and 4; HRESIMS *m*/*z*: 633.3792 [M + H] ^+^ calcd for C_39_H_53_O_7_, 633.3786.

### Serratic Acid C (**8**)

White powder; [α]^25^_D_ – 69.1 (*c* 0.5, MeOH); UV (MeOH) λ_max_ (log ε) 322.5 (0.73), 241.7 (1.29) nm; IR (KBr) ν_max_ 3410, 2862, 1705, 1597, 1512, 1458, 1366, 1273, 1042 cm^–1^; ^1^H (pyridine-*d*_5_, 600 MHz) and ^13^C (pyridine-*d*_5_, 150 MHz) NMR data, see Tables 3 and 4; HRESIMS *m*/*z*: 663.3896 [M + H] ^+^ calcd for C_30_H_47_O_5_, 663.3891.

### Serratic Acid D (**9**)

White powder; [α]^25^_D_ – 71.6 (*c* 0.1, MeOH); UV (MeOH) λ_max_ (log ε) 195 (0.65) nm; IR (KBr) ν_max_ 3400, 2870, 1713, 1597, 1458, 1373, 1250, 1049 cm^–1^; ^1^H (pyridine-*d*_5_, 600 MHz) and ^13^C (pyridine-*d*_5_, 150 MHz) NMR data, see Tables 3 and 4; HRESIMS *m*/*z*: 504.3682 [M + NH_4_] ^+^ calcd for C_30_H_46_O_5_Na, 504.3684.

### Cell Lines and Cell Culture

The melanoma cells B16–F10 and A375, the lung tumor cell line A549, and the breast cancer cell line MDA-MB-231 were obtained from ATCC, USA. All cells were cultured in DMEM medium supplemented with 10% FBS, penicillin G (100 U/mL), and streptomycin (100 μg/mL) in a humidified atmosphere with 5% CO_2_ at 37 °C.

### In Vitro Cytotoxicity Activity

Cytotoxicity assays involving B16–F10, A375, A549, and MDA-MB-231 cells were performed in 96-well microplates by MTT method. Four tumor cells were treated with or without 1.25, 2.5, 5, 10, and 20 μM of **2**, **6**, **10**–**14**, **16**, **17, 19**, **21**, and **26** for 48 h. Then, 20 μL of MTT solution (5 mg/mL) was added to each well and further incubated for 4 h. The OD was then measured at 570 nm on a microplate reader in each case. Paclitaxel was used as the positive control for all the experiments. IC_50_ values were calculated using SPSS 20.0 statistical software.

### Determination of Melanin Content

B16–F10 cells were seeded at a density of 1  ×  10^6^ cells/well in 12-well plates and incubated for 24 h. Cells were treated with test compounds **7, 8**, **33**–**36**, and **38** (5 and 10 μM) and 10 nM α-MSH for an additional 72 h. KA (800 μM) was used as a positive control. After incubation, harvested cell pellets were resuspended in 10% DMSO in 1 N NaOH and heated to 80 °C for 1 h, and the absorbance at 405 nm was measured.

## Supporting Information Available

The HRESIMS, UV, IR, CD, ^1^H, ^13^C, and 2D NMR spectra for all new compounds **1**–**9** and the ^1^H and ^13^C NMR spectra for all known compounds **10**–**41** (PDF)
