# Supplementary material for: Diterpenoids and Triterpenoids from the Aerial Parts of Isodon serra and Their Biological Activities
Source: ACS Omega. 2024 Nov 22;9(49):48791–801. doi: 10.1021/acsomega.4c08821 (PMC11635516; doi:10.1021/acsomega.4c08821)
Supplement: Supplementary file 1 — ao4c08821_si_001.pdf [file ao4c08821_si_001.pdf]

Supporting Information

Diterpenoids and Triterpenoids from the Aerial Parts of *Isodon serra* and Their Biological Activities

Wen-Jing Ren,[†] Rong Jiang,[†] Kei-Fong Ng,[†] Meng-Yu Bao,[†] Xiao-Mei Liu,[†] Wei Zhang,[†] Zhi-Hong Jiang,[†] Yu-Hong Liu,*[,†,‡] and Guo-Yuan Zhu*[,†]

[†]*State Key Laboratory of Quality Research in Chinese Medicine, Guangdong-Hong Kong-Macao Joint Laboratory of Respiratory Infectious Disease, Macau Institute for Applied Research in Medicine and Health, Macau University of Science and Technology, Macau 999078, People's Republic of China*

[‡]*School of Pharmaceutical Sciences, Shandong University of Traditional Chinese Medicine, Jinan 250355, People's Republic of China*

* To whom correspondence should be addressed. E-mail: yhliu@sdutcm.edu.cn; gyzhu@must.edu.mo

Table of Contents

Figure S1. The HRESIMS spectrum of compound **1** 8

Figure S2. The ^{1}H NMR spectrum of compound **1** 8

Figure S3. The ^{13}C and DEPT 135 NMR spectra of compound **1** 9

Figure S4. The HSQC spectrum of compound **1** 9

Figure S5. The HMBC spectrum of compound **1** 10

Figure S6. The ^{1}H-^{1}H COSY spectrum of compound **1** 10

Figure S7. The NOESY spectrum of compound **1** 11

Figure S8. The UV spectrum of compound **1** 11

Figure S9. The IR spectrum of compound **1** 12

Figure S10. The CD spectrum of compound **1** 12

Figure S11. The HRESIMS spectrum of compound **2** 13

Figure S12. The ^{1}H NMR spectrum of compound **2** 13

Figure S13. The ^{13}C and DEPT 135 NMR spectra of compound **2** 14

Figure S14. The HSQC spectrum of compound **2** 14

Figure S15. The HMBC spectrum of compound **2** 15

Figure S16. The ^{1}H-^{1}H COSY spectrum of compound **2** 15

Figure S17. The NOESY spectrum of compound **2** 16

Figure S18. The UV spectrum of compound **2** 16

Figure S19. The IR spectrum of compound **2** 17

Figure S20. The CD spectrum of compound **2** 17

Figure S21. The HRESIMS spectrum of compound **3** 18

Figure S22. The ^{1}H NMR spectrum of compound **3** 18

Figure S23. The ^{13}C and DEPT 135 NMR spectra of compound **3** 19

Figure S24. The HSQC spectrum of compound **3** 19

Figure S25. The HMBC spectrum of compound **3** 20

Figure S26. The ^{1}H-^{1}H COSY spectrum of compound **3** 20

Figure S27. The NOESY spectrum of compound **3** 21

Figure S28. The UV spectrum of compound **3** 21
Figure S29. The IR spectrum of compound **3** 22
Figure S30. The CD spectrum of compound **3**..22
Figure S31. The HRESIMS spectrum of compound **4**...23
Figure S32. The ^{1}H NMR spectrum of compound **4**...23
Figure S33. The ^{13}C and DEPT 135 NMR spectra of compound **4** 24
Figure S34. The HSQC spectrum of compound **4** 24
Figure S35. The HMBC spectrum of compound **4** 25
Figure S36. The ^{1}H-^{1}H COSY spectrum of compound **4**..25
Figure S37. The NOESY spectrum of compound **4**...26
Figure S38. The UV spectrum of compound **4** 26
Figure S39. The IR spectrum of compound **4** 27
Figure S40. The CD spectrum of compound **4**..27
Figure S41. The HRESIMS spectrum of compound **5**...28
Figure S42. The ^{1}H NMR spectrum of compound **5**...28
Figure S43. The ^{13}C and DEPT 135 NMR spectra of compound **5** 29
Figure S44. The HSQC spectrum of compound **5** 29
Figure S45. The HMBC spectrum of compound **5** 30
Figure S46. The ^{1}H-^{1}H COSY spectrum of compound **5**..30
Figure S47. The NOESY spectrum of compound **5**...31
Figure S48. The UV spectrum of compound **5** 31
Figure S49. The IR spectrum of compound **5** 32
Figure S50. The CD spectrum of compound **5**..32
Figure S51. The HRESIMS spectrum of compound **6**...33
Figure S52. The ^{1}H NMR spectrum of compound **6**...33
Figure S53. The ^{13}C and DEPT 135 NMR spectra of compound **6** 34
Figure S54. The HSQC spectrum of compound **6** 34
Figure S55. The HMBC spectrum of compound **6** 35
Figure S56. The ^{1}H-^{1}H COSY spectrum of compound **6**..35
Figure S57. The UV spectrum of compound **6** 36

Figure S58. The IR spectrum of compound **6** 36
Figure S59. The CD spectrum of compound **6**..37
Figure S60. The HRESIMS spectrum of compound **7**..37
Figure S61. The ^{1}H NMR spectrum of compound 7..38
Figure S62. The ^{13}C and DEPT 135 NMR spectra of compound **7**...........................38
Figure S63. The HSQC spectrum of compound **7** 39
Figure S64. The HMBC spectrum of compound **7** 39
Figure S65. The ^{1}H-^{1}H COSY spectrum of compound **7**..40
Figure S66. The UV spectrum of compound **7** 40
Figure S67. The IR spectrum of compound **7** 41
Figure S68. The CD spectrum of compound **7**..41
Figure S69. The HRESIMS spectrum of compound **8**..42
Figure S70. The ^{1}H NMR spectrum of compound **8**..42
Figure S71. The ^{13}C and DEPT 135 NMR spectra of compound **8**...........................43
Figure S72. The HSQC spectrum of compound **8** 43
Figure S73. The HMBC spectrum of compound **8** 44
Figure S74. The ^{1}H-^{1}H COSY spectrum of compound **8**..44
Figure S75. The UV spectrum of compound **8** 45
Figure S76. The IR spectrum of compound **8** 45
Figure S77. The CD spectrum of compound **8**..46
Figure S78. The HRESIMS spectrum of compound **9**..46
Figure S79. The ^{1}H NMR spectrum of compound **9**..47
Figure S80. The ^{13}C and DEPT 135 NMR spectra of compound **9**...........................47
Figure S81. The HSQC spectrum of compound **9** 48
Figure S82. The HMBC spectrum of compound **9** 48
Figure S83. The ^{1}H-^{1}H COSY spectrum of compound **9**..49
Figure S84. The UV spectrum of compound **9** 49
Figure S85. The IR spectrum of compound **9** 50
Figure S86. The CD spectrum of compound **9**..50
Figure S87. The ^{1}H NMR spectrum of compound **10**..51

Figure S88. The ^{13}C and DEPT 135 NMR spectra of compound **10** 51
Figure S89. The ^{1}H NMR spectrum of compound **11** 52
Figure S90. The ^{13}C and DEPT 135 NMR spectra of compound **11** 52
Figure S91. The ^{1}H NMR spectrum of compound **12** 53
Figure S92. The ^{13}C and DEPT 135 NMR spectra of compound **12** 53
Figure S93. The ^{1}H NMR spectrum of compound **13** 54
Figure S94. The ^{13}C and DEPT 135 NMR spectra of compound **13** 54
Figure S95. The ^{1}H NMR spectrum of compound **14** 55
Figure S96. The ^{13}C and DEPT 135 NMR spectra of compound **14** 55
Figure S97. The ^{1}H NMR spectrum of compound **15** 56
Figure S98. The ^{13}C and DEPT 135 NMR spectra of compound 15 56
Figure S99. The ^{1}H NMR spectrum of compound **16** 57
Figure S100. The ^{13}C and DEPT 135 NMR spectra of compound **16** 57
Figure S101. The ^{1}H NMR spectrum of compound **17** 58
Figure S102. The ^{13}C and DEPT 135 NMR spectra of compound **17** 58
Figure S103. The ^{1}H NMR spectrum of compound **18** 59
Figure S104. The ^{13}C and DEPT 135 NMR spectra of compound **18** 59
Figure S105. The ^{1}H NMR spectrum of compound **19** 60
Figure S106. The ^{13}C and DEPT 135 NMR spectra of compound **19** 60
Figure S107. The ^{1}H NMR spectrum of compound **20** 61
Figure S108. The ^{13}C and DEPT 135 NMR spectra of compound **20** 61
Figure S109. The ^{1}H NMR spectrum of compound **21** 62
Figure S110. The ^{13}C and DEPT 135 NMR spectra of compound **21** 62
Figure S111. The ^{1}H NMR spectrum of compound **22** 63
Figure S112. The ^{13}C and DEPT 135 NMR spectra of compound **22** 63
Figure S113. The ^{1}H NMR spectrum of compound **23** 64
Figure S114. The ^{13}C and DEPT 135 NMR spectra of compound **23** 64
Figure S115. The ^{1}H NMR spectrum of compound **24** 65
Figure S116. The ^{13}C and DEPT 135 NMR spectra of compound **24** 65
Figure S117. The ^{1}H NMR spectrum of compound **25** 66

Figure S118. The ^{13}C and DEPT 135 NMR spectra of compound **25** 66
Figure S119. The ^{1}H NMR spectrum of compound **26**...67
Figure S120. The ^{13}C and DEPT 135 NMR spectra of compound **26** 67
Figure S121. The ^{1}H NMR spectrum of compound **27**...68
Figure S122. The ^{13}C and DEPT 135 NMR spectra of compound **27** 68
Figure S123. The ^{1}H NMR spectrum of compound **28**...69
Figure S124. The ^{13}C and DEPT 135 NMR spectra of compound **28** 69
Figure S125. The ^{1}H NMR spectrum of compound **29**...70
Figure S126. The ^{13}C and DEPT 135 NMR spectra of compound **29** 70
Figure S127. The ^{1}H NMR spectrum of compound **30**...71
Figure S128. The ^{13}C and DEPT 135 NMR spectra of compound **30** 71
Figure S129. The ^{1}H NMR spectrum of compound **31**...72
Figure S130. The ^{13}C and DEPT 135 NMR spectra of compound **31** 72
Figure S131. The ^{1}H NMR spectrum of compound **32**...73
Figure S132. The ^{13}C and DEPT 135 NMR spectra of compound **32** 73
Figure S133. The ^{1}H NMR spectrum of compound **33**...74
Figure S134. The ^{13}C and DEPT 135 NMR spectra of compound **33** 74
Figure S135. The ^{1}H NMR spectrum of compound **34**...75
Figure S136. The ^{13}C and DEPT 135 NMR spectra of compound **34** 75
Figure S137. The ^{1}H NMR spectrum of compound **35**...76
Figure S138. The ^{13}C and DEPT 135 NMR spectra of compound **35** 76
Figure S139. The ^{1}H NMR spectrum of compound **36**...77
Figure S140. The ^{13}C and DEPT 135 NMR spectra of compound **36** 77
Figure S141. The ^{1}H NMR spectrum of compound **37**...78
Figure S142. The ^{13}C and DEPT 135 NMR spectra of compound **37** 78
Figure S143. The ^{1}H NMR spectrum of compound **38**...79
Figure S144. The ^{13}C and DEPT 135 NMR spectra of compound **38** 79
Figure S145. The ^{1}H NMR spectrum of compound **39**...80
Figure S146. The ^{13}C and DEPT 135 NMR spectra of compound **39** 80
Figure S147. The ^{1}H NMR spectrum of compound **40**...81

Figure S148. The ^{13}C and DEPT 135 NMR spectra of compound **40** 81
Figure S149. The ^{1}H NMR spectrum of compound **41** 82
Figure S150. The ^{13}C and DEPT 135 NMR spectra of compound **41** 82
Table S1. ^{1}H (600 MHz) and ^{13}C (150 MHz) NMR data of compound **23** in pyridine-*d*$_5$. 83
Table S2. ^{1}H (600 MHz) and ^{13}C (150 MHz) NMR data of compound **28** in MeOD. 84
Table S3. ^{1}H (600 MHz) and ^{13}C (150 MHz) NMR data of compound **40** in $CDCl_3$. 85

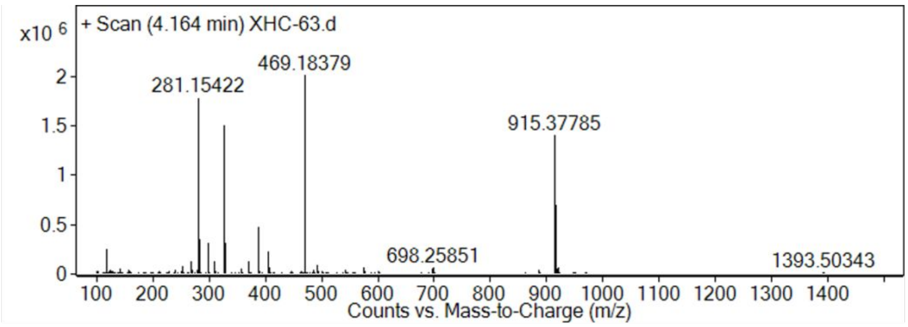

Formula	Score	Mass	Mass (MFG)	*m/z* (Calc)	Diff (ppm)	Ion Formula	*m/z*
$C_{24}H_{30}O_8$	99.39	446.1946	446.1941	469.1833	-1.15	$C_{24}H_{30}O_8Na$	469.1838

Figure S1. The HRESIMS spectrum of compound 1

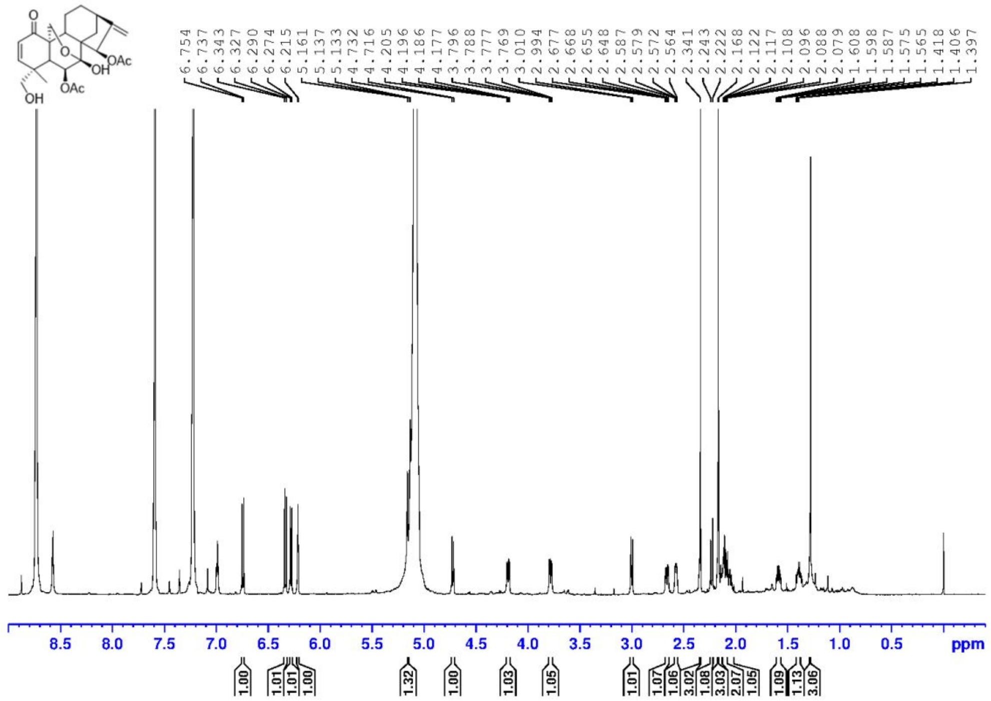

Figure S2. The ^{1}H NMR spectrum of compound 1

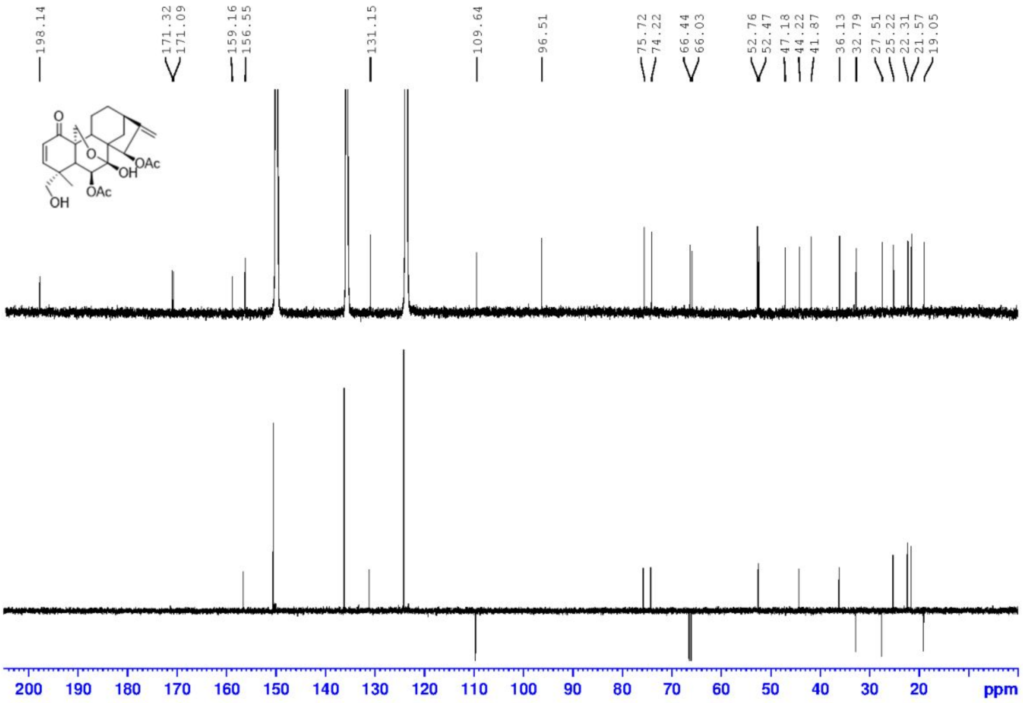

Figure S3. The ^{13}C and DEPT 135 NMR spectra of compound 1

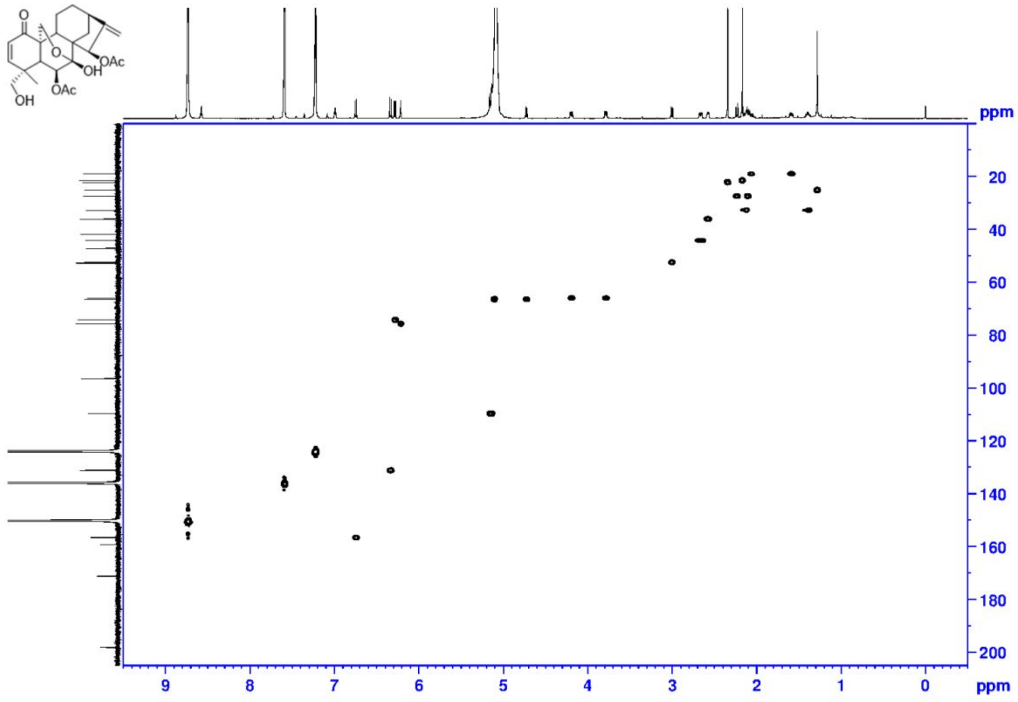

Figure S4. The HSQC spectrum of compound 1

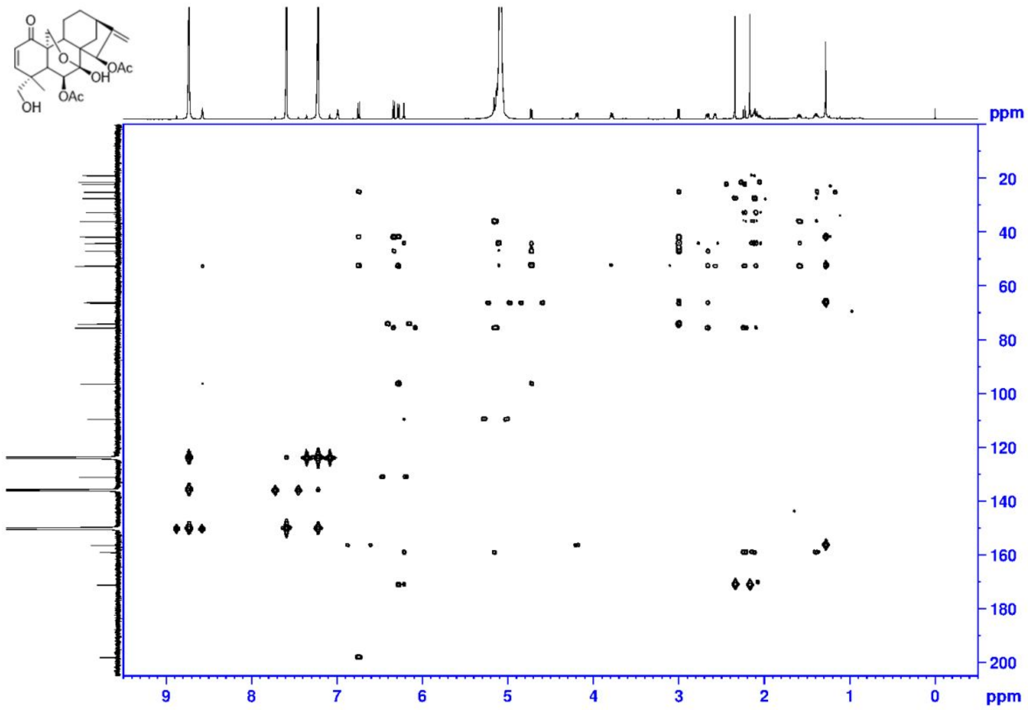

Figure S5. The HMBC spectrum of compound 1

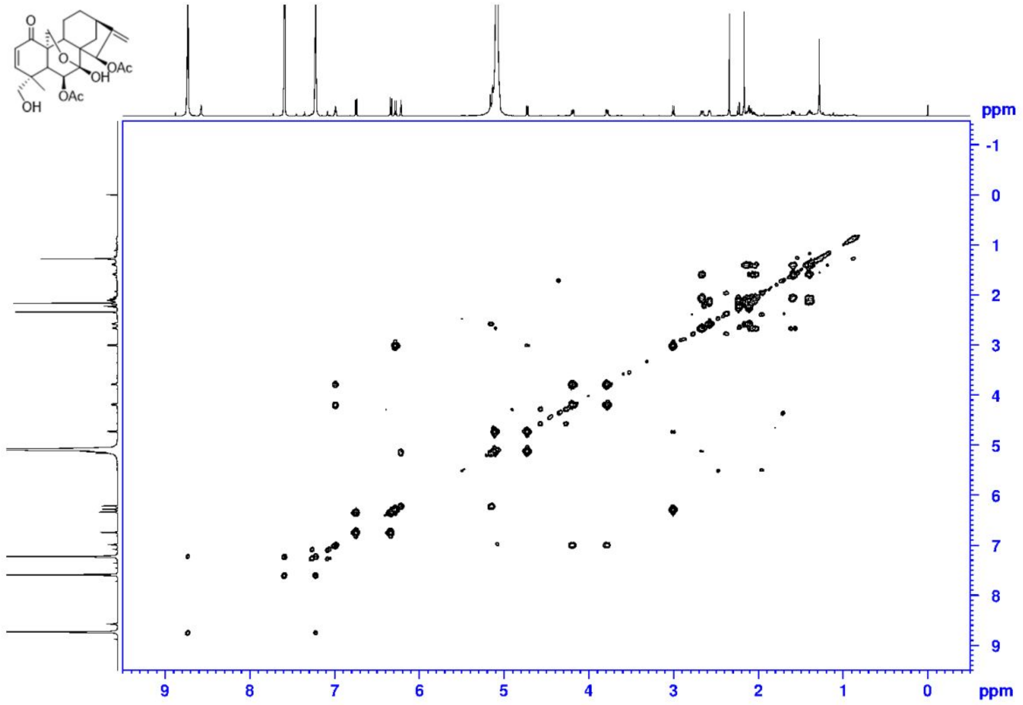

Figure S6. The ^{1}H-^{1}H COSY spectrum of compound 1

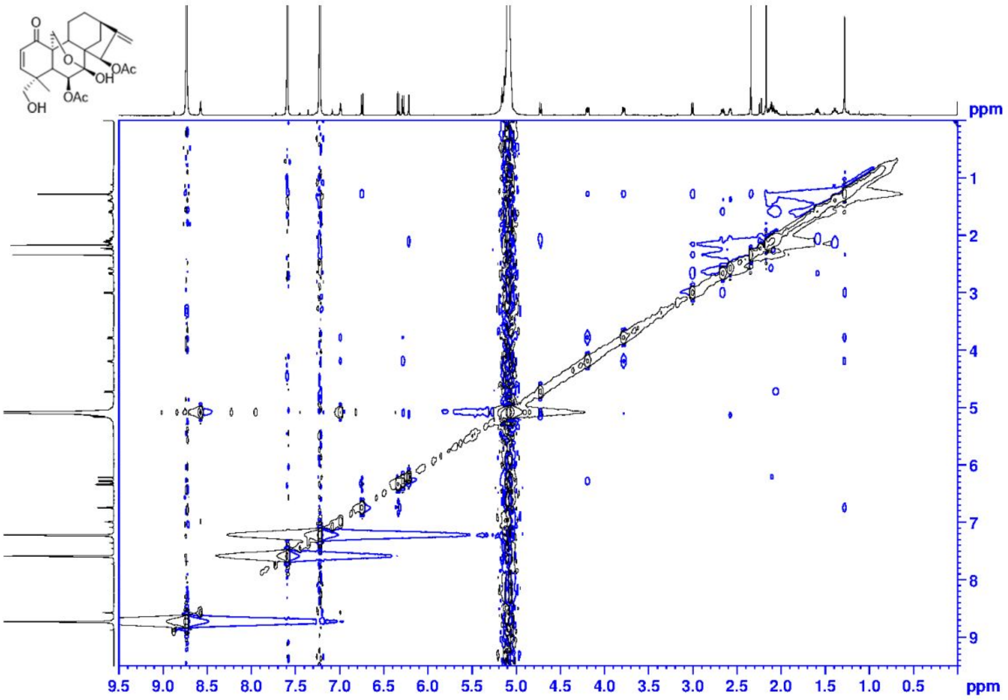

Figure S7. The NOESY spectrum of compound 1

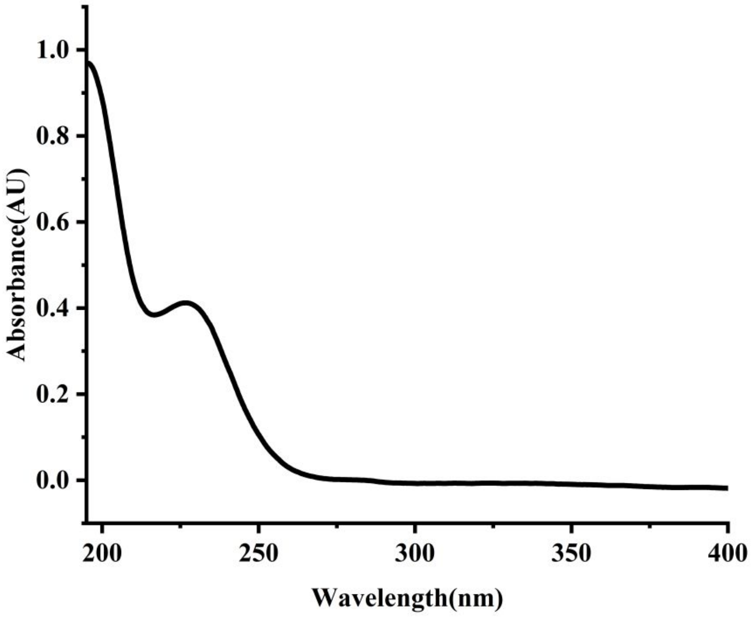

Figure S8. The UV spectrum of compound 1

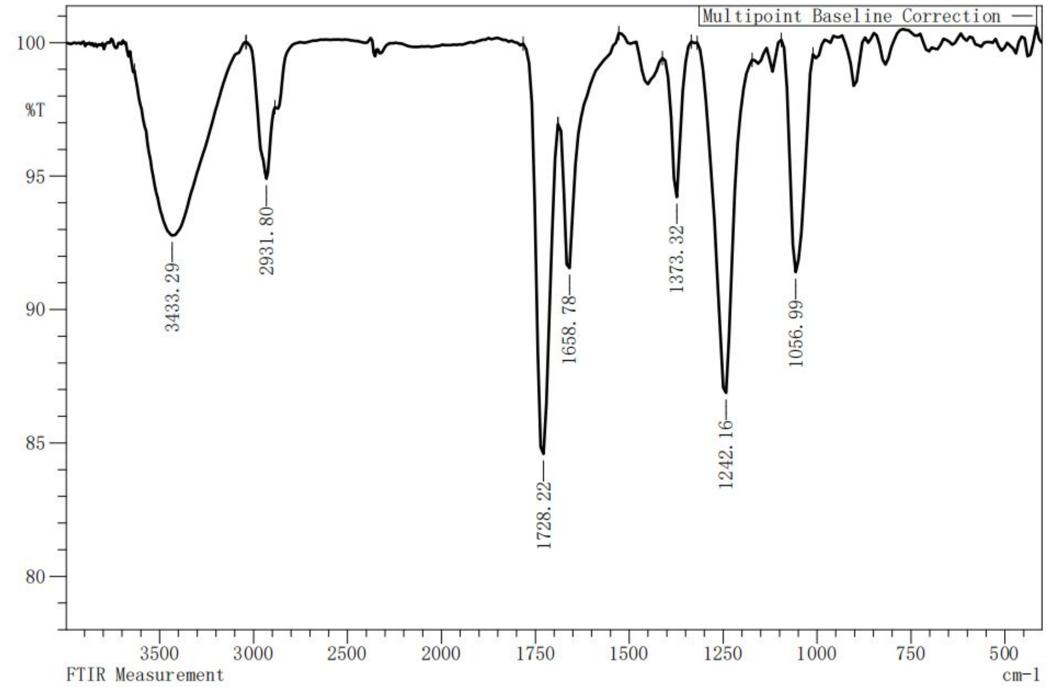

Figure S9. The IR spectrum of compound 1

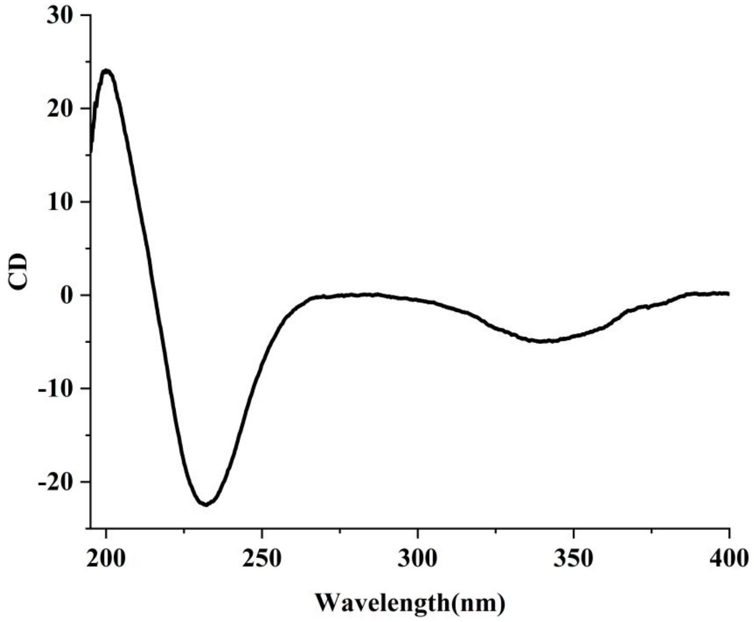

Figure S10. The CD spectrum of compound 1

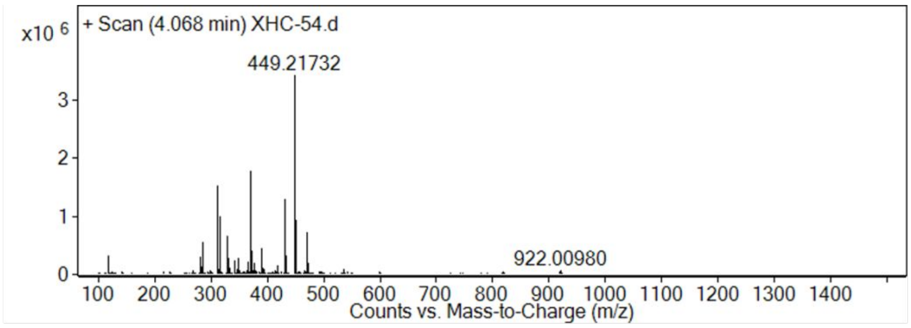

Formula	Score	Mass	Mass (MFG)	*m/z* (Calc)	Diff (ppm)	Ion Formula	*m/z*
$C_{24}H_{32}O_8$	99.78	448.2100	448.2097	449.2170	-0.68	$C_{24}H_{33}O_8$	449.2173

Figure S11. The HRESIMS spectrum of compound 2

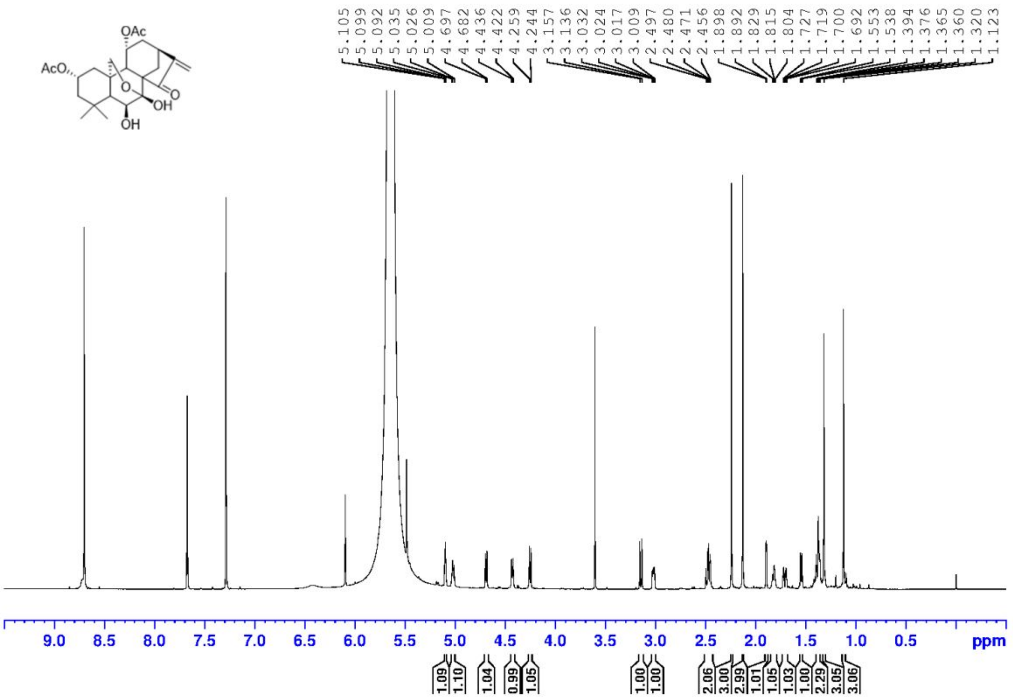

Figure S12. The ^{1}H NMR spectrum of compound 2

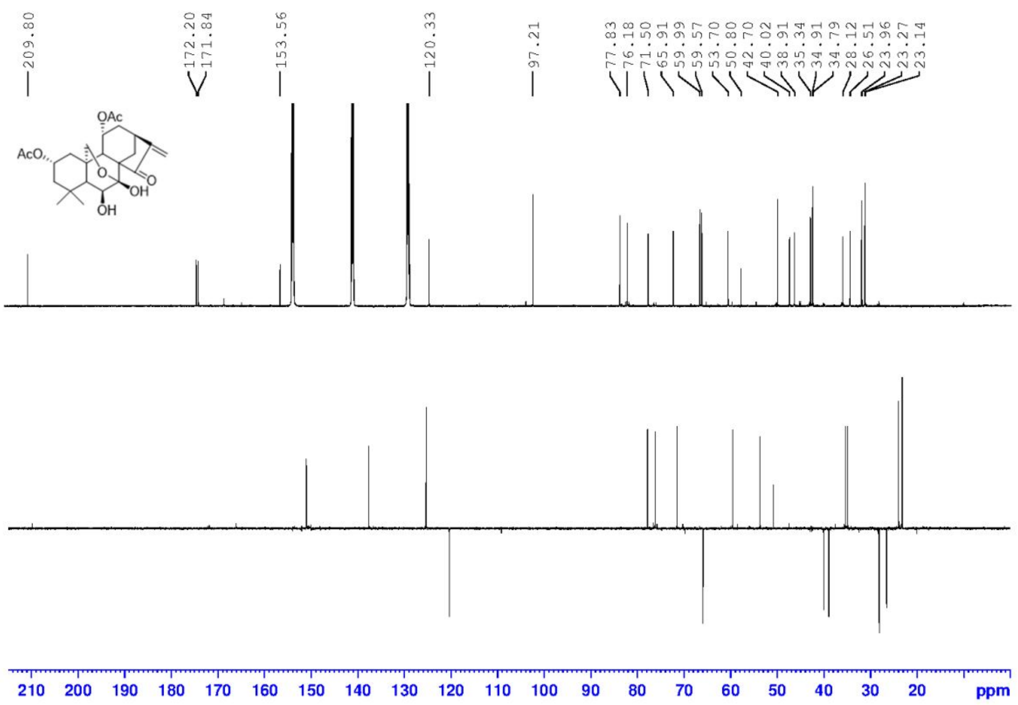

Figure S13. The ^{13}C and DEPT 135 NMR spectra of compound 2

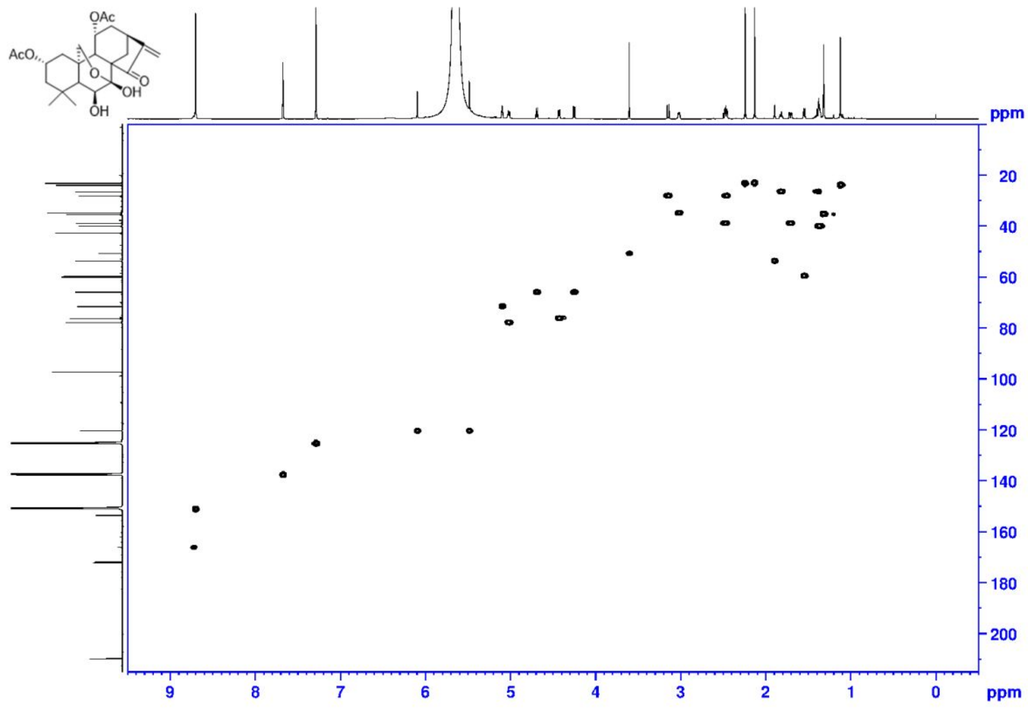

Figure S14. The HSQC spectrum of compound 2

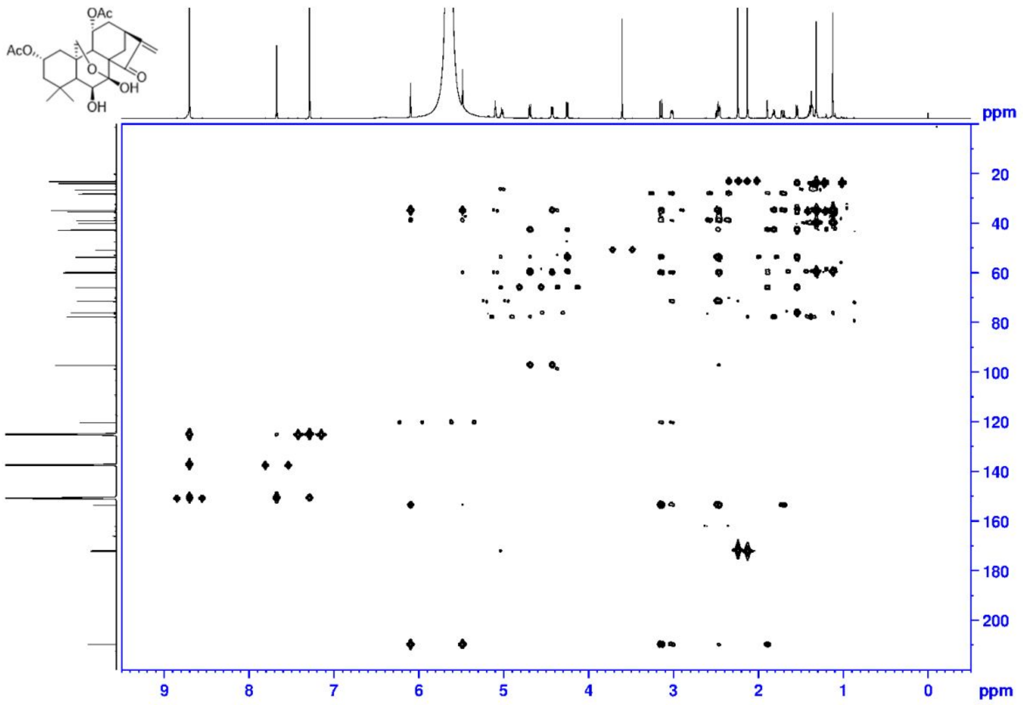

Figure S15. The HMBC spectrum of compound 2

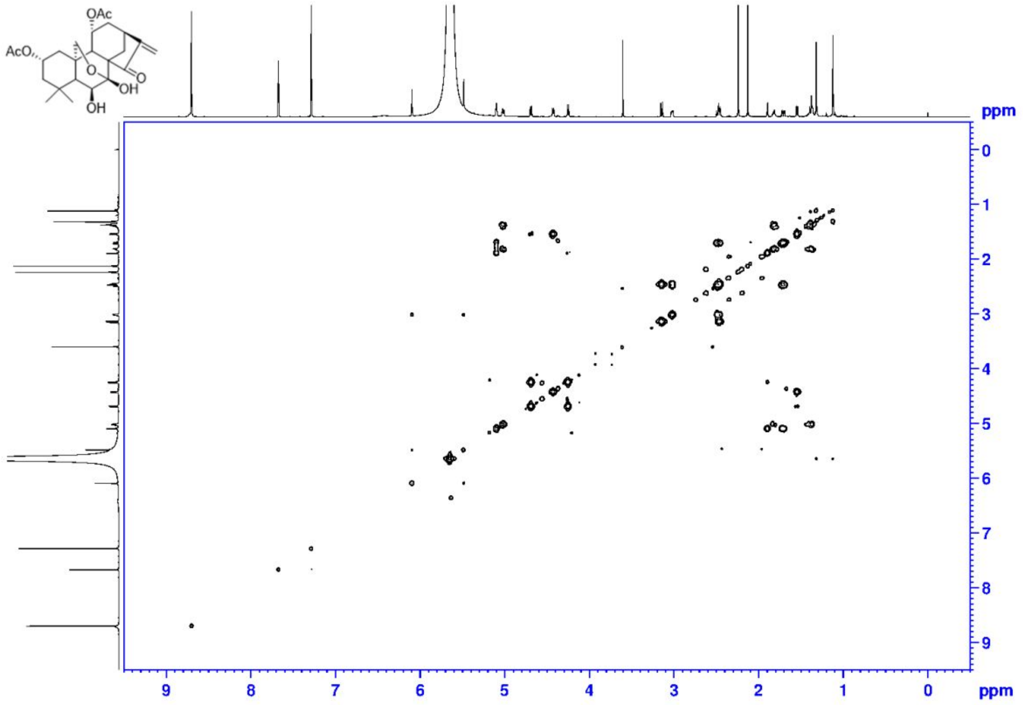

Figure S16. The ^{1}H-^{1}H COSY spectrum of compound 2

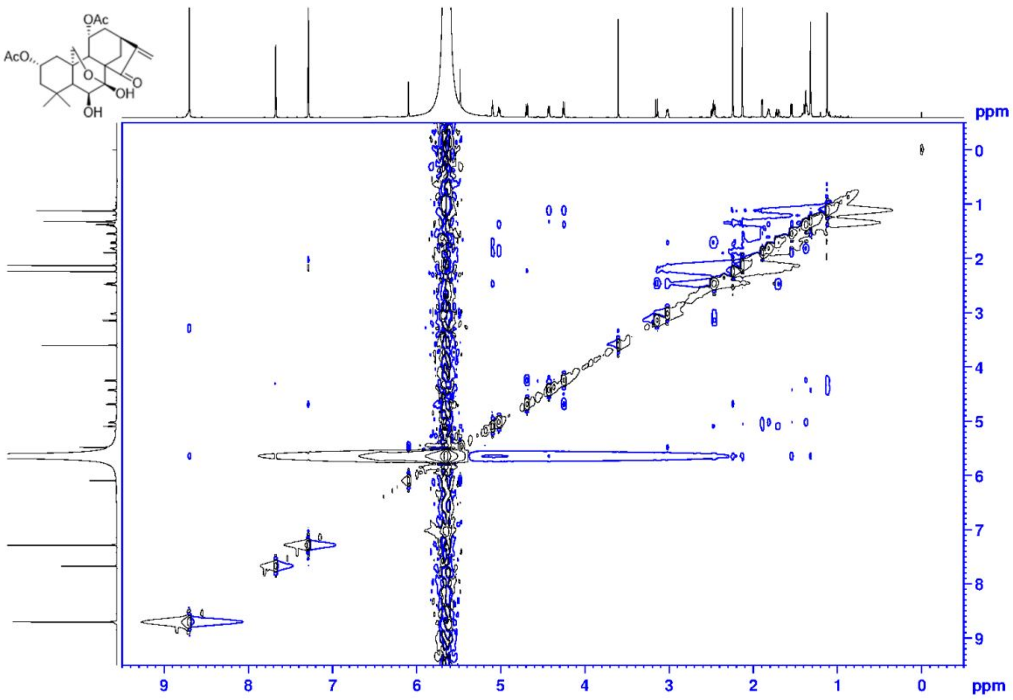

Figure S17. The NOESY spectrum of compound 2

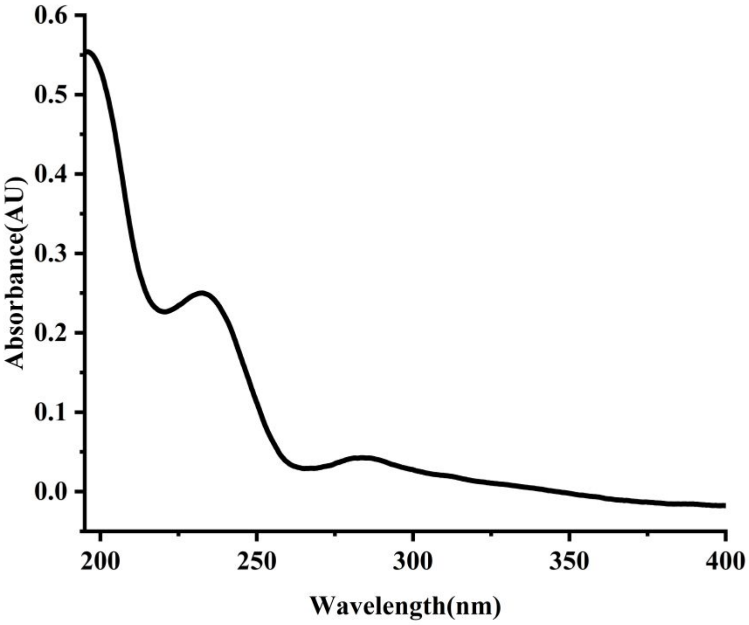

Figure S18. The UV spectrum of compound 2

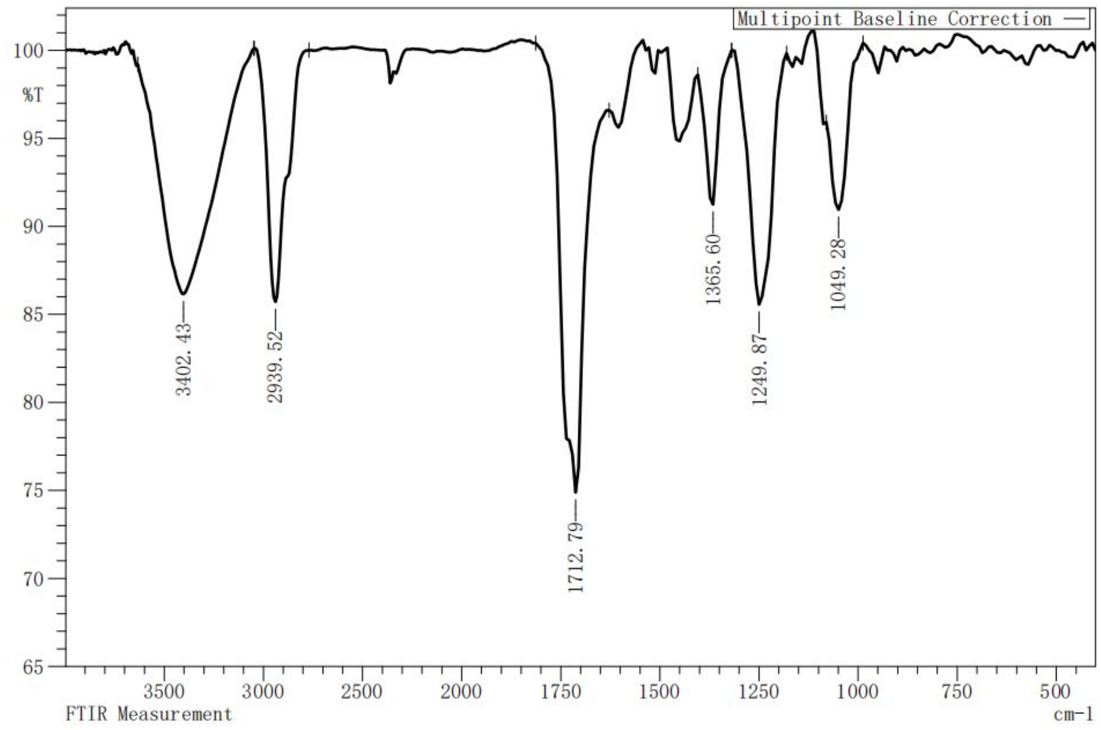

Figure S19. The IR spectrum of compound 2

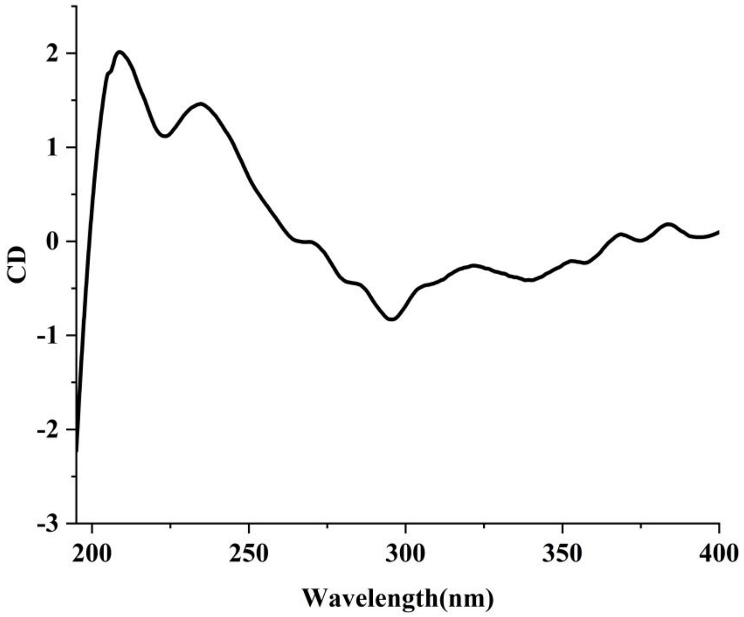

Figure S20. The CD spectrum of compound 2

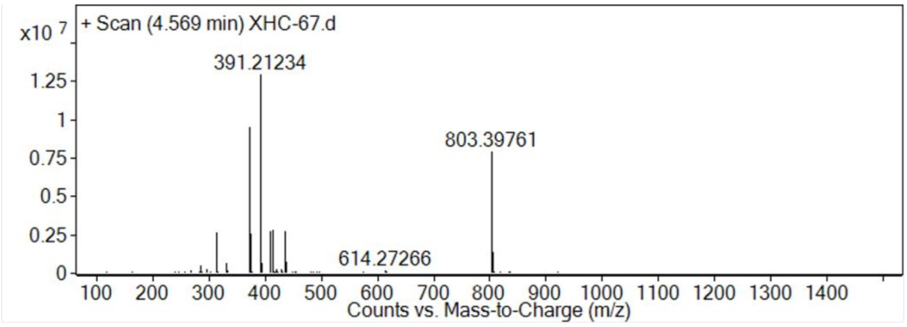

Formula	Score	Mass	Mass (MFG)	*m/z* (Calc)	Diff (ppm)	Ion Formula	*m/z*
$C_{22}H_{30}O_6$	98.35	390.205	390.2042	391.2115	-2.01	$C_{22}H_{31}O_6$	391.2123

Figure S21. The HRESIMS spectrum of compound 3

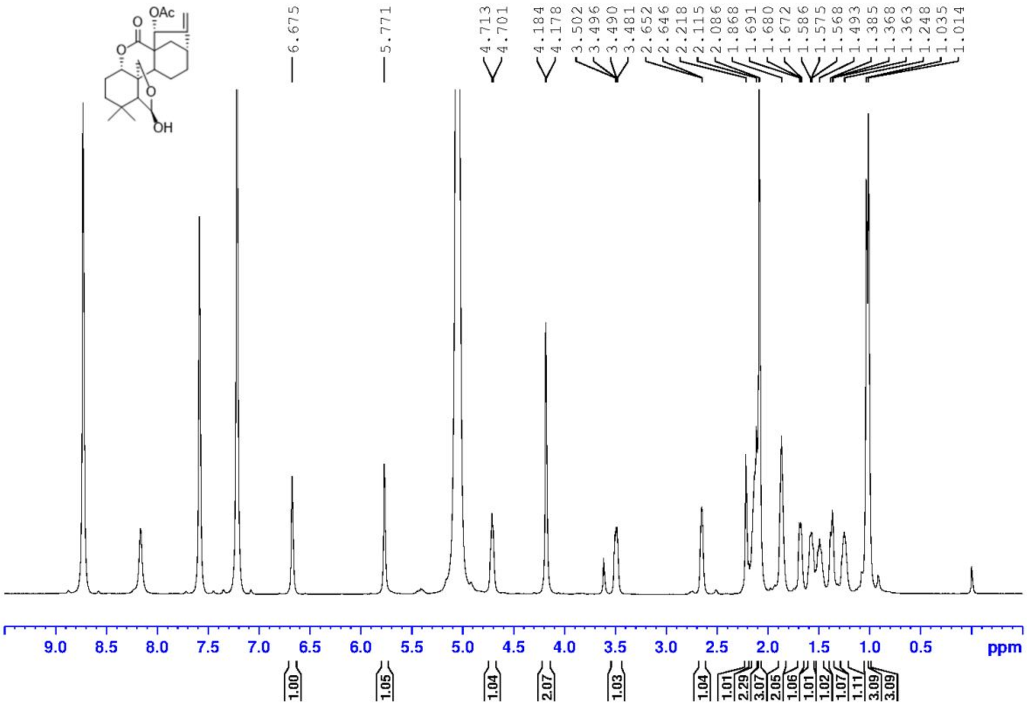

Figure S22. The ^{1}H NMR spectrum of compound 3

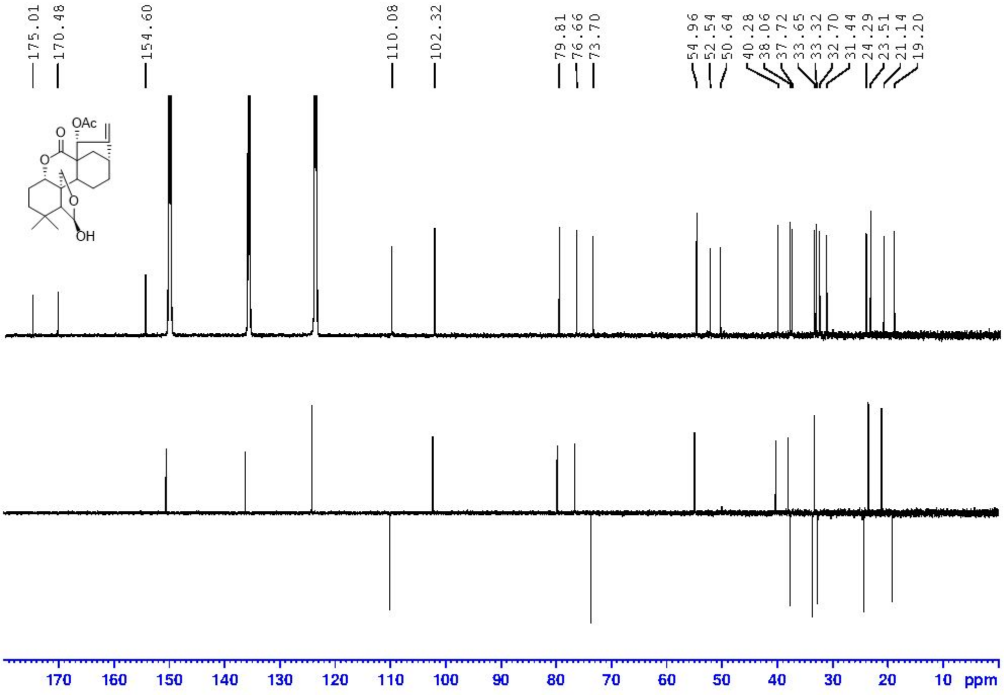

Figure S23. The ^{13}C and DEPT 135 NMR spectra of compound 3

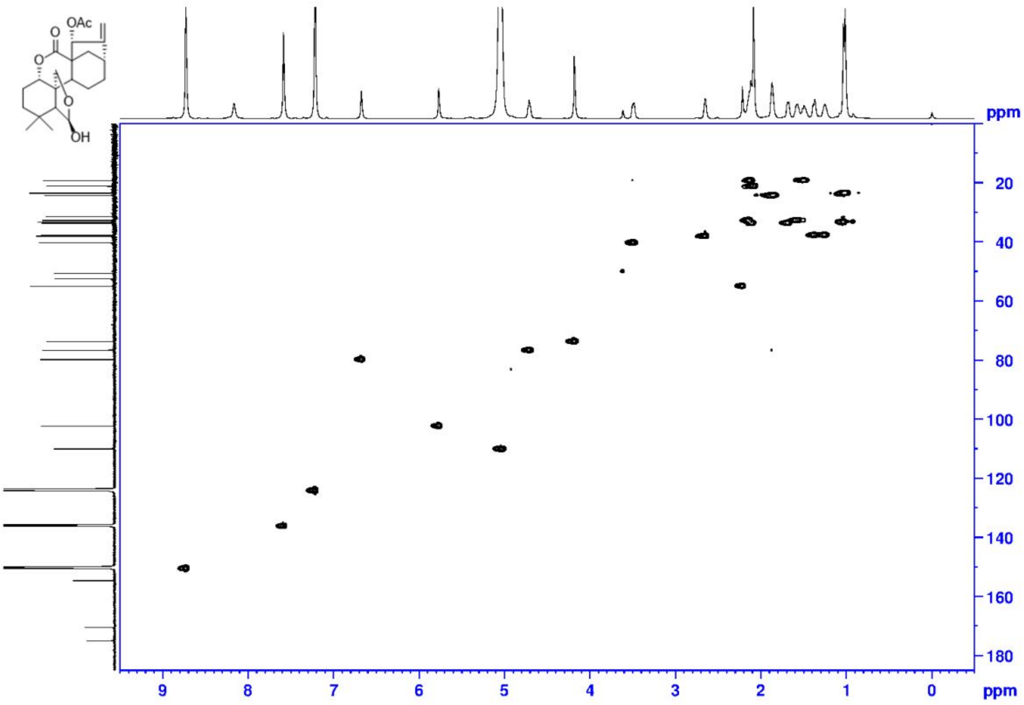

Figure S24. The HSQC spectrum of compound 3

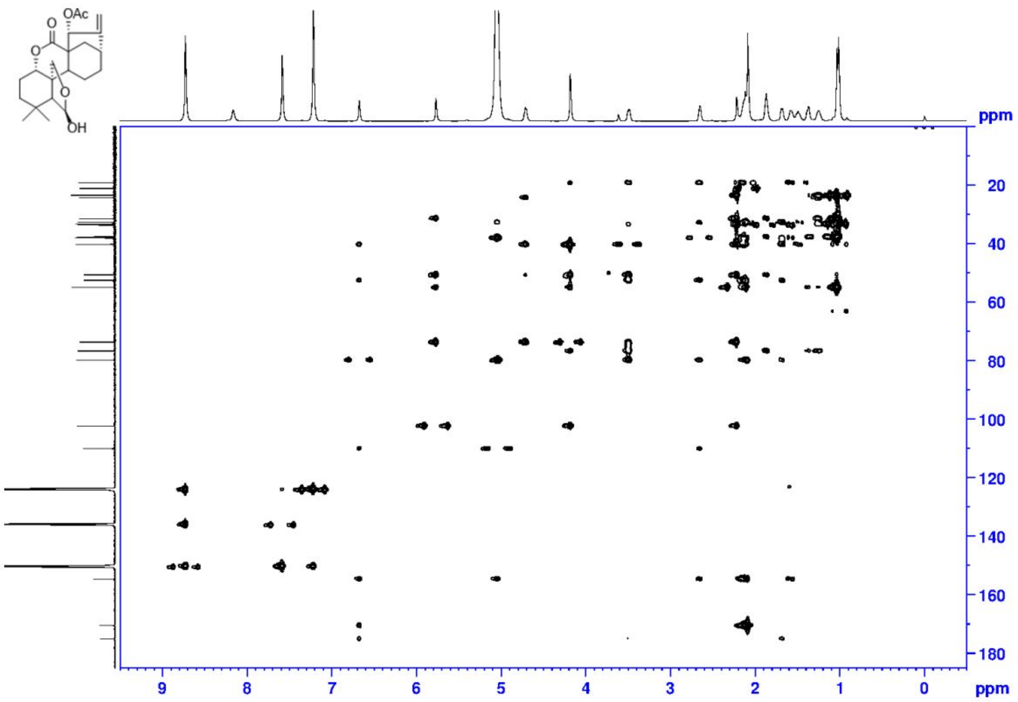

Figure S25. The HMBC spectrum of compound 3

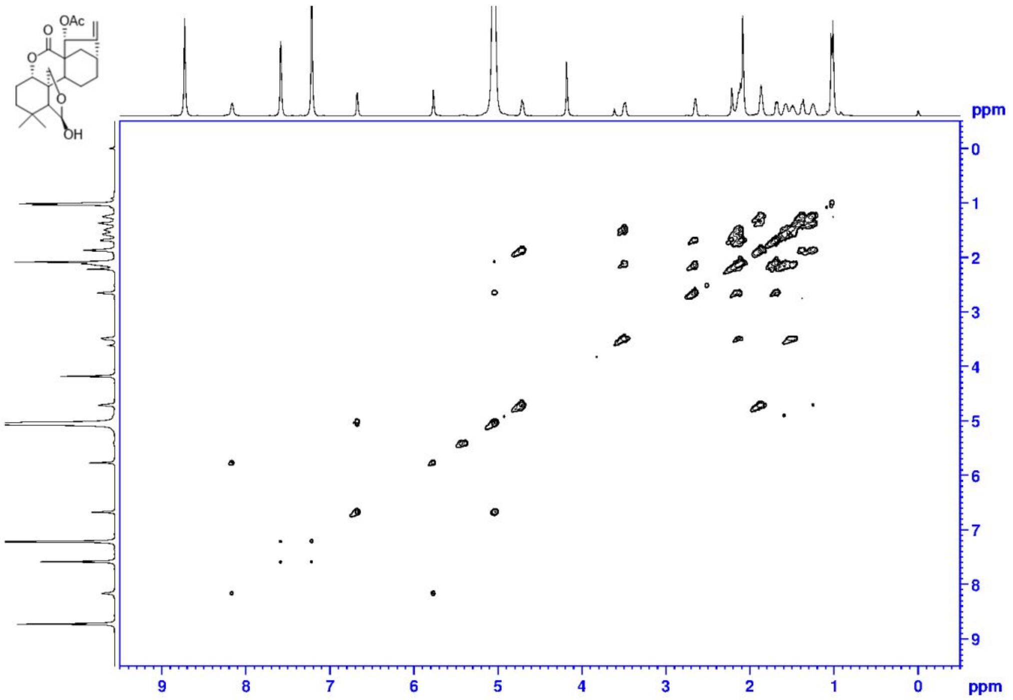

Figure S26. The ^{1}H-^{1}H COSY spectrum of compound 3

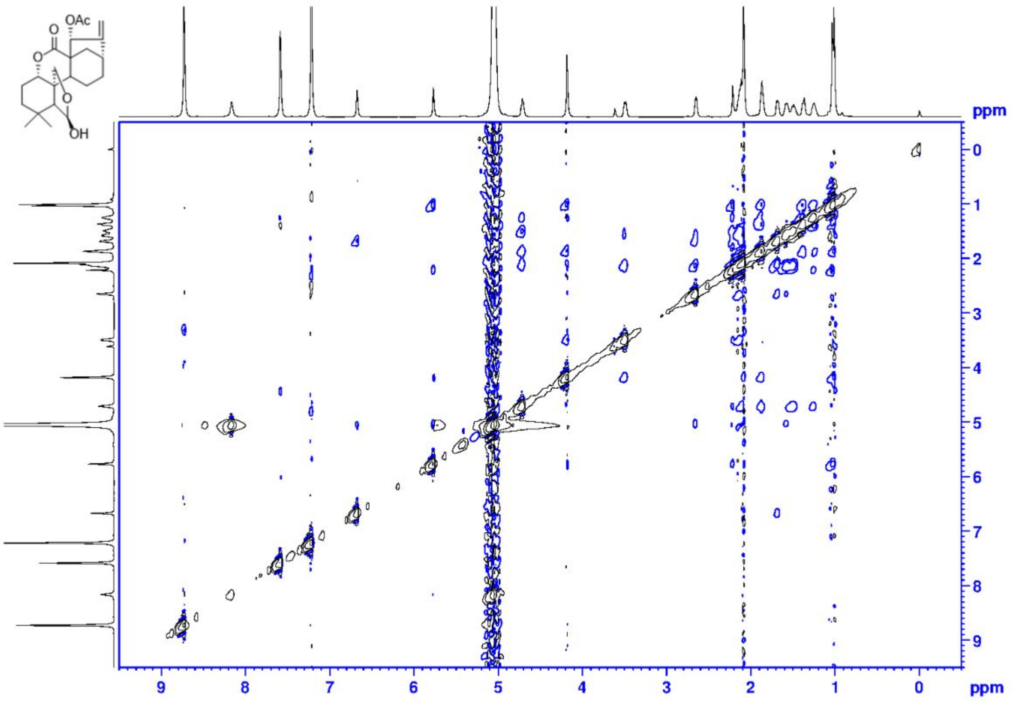

Figure S27. The NOESY spectrum of compound 3

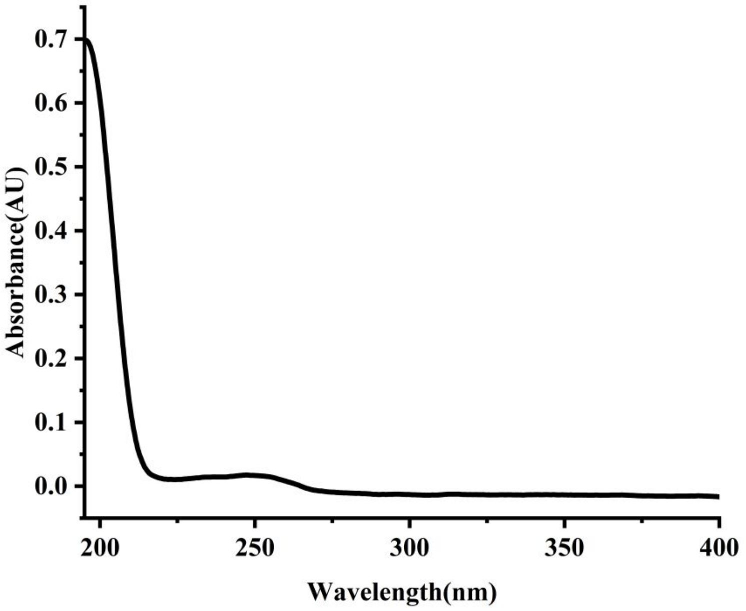

Figure S28. The UV spectrum of compound 3

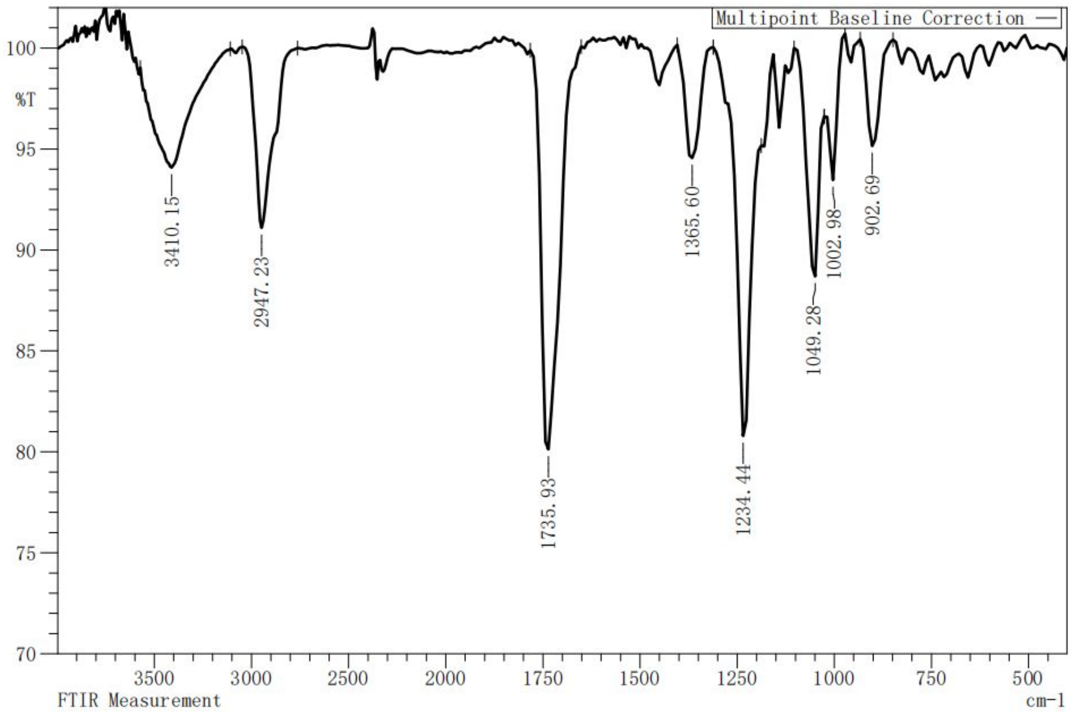

Figure S29. The IR spectrum of compound 3

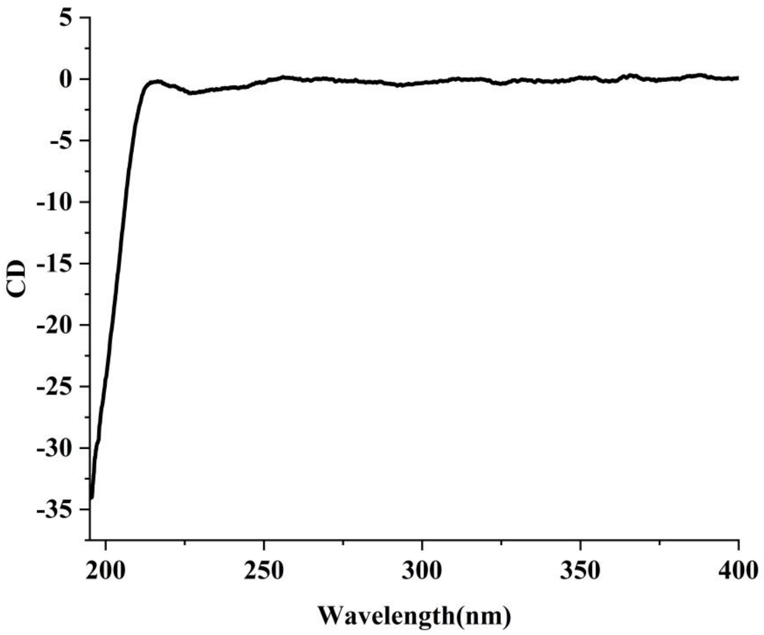

Figure S30. The CD spectrum of compound 3

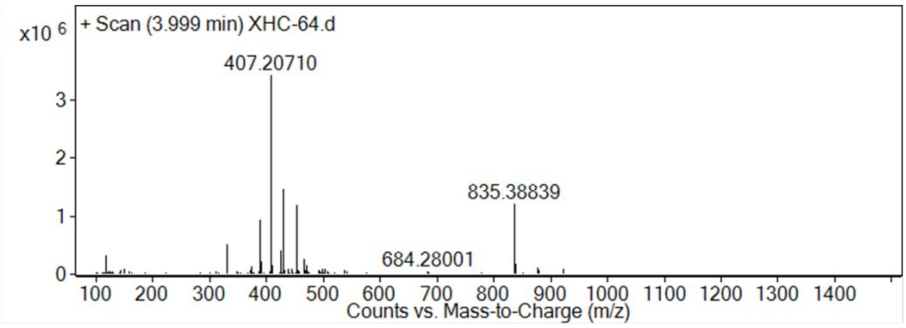

Formula	Score	Mass	Mass (MFG)	*m/z* (Calc)	Diff (ppm)	Ion Formula	*m/z*
$C_{22}H_{30}O_7$	98.84	406.1998	406.1992	407.2064	-1.65	$C_{22}H_{31}O_7$	407.2071

Figure S31. The HRESIMS spectrum of compound 4

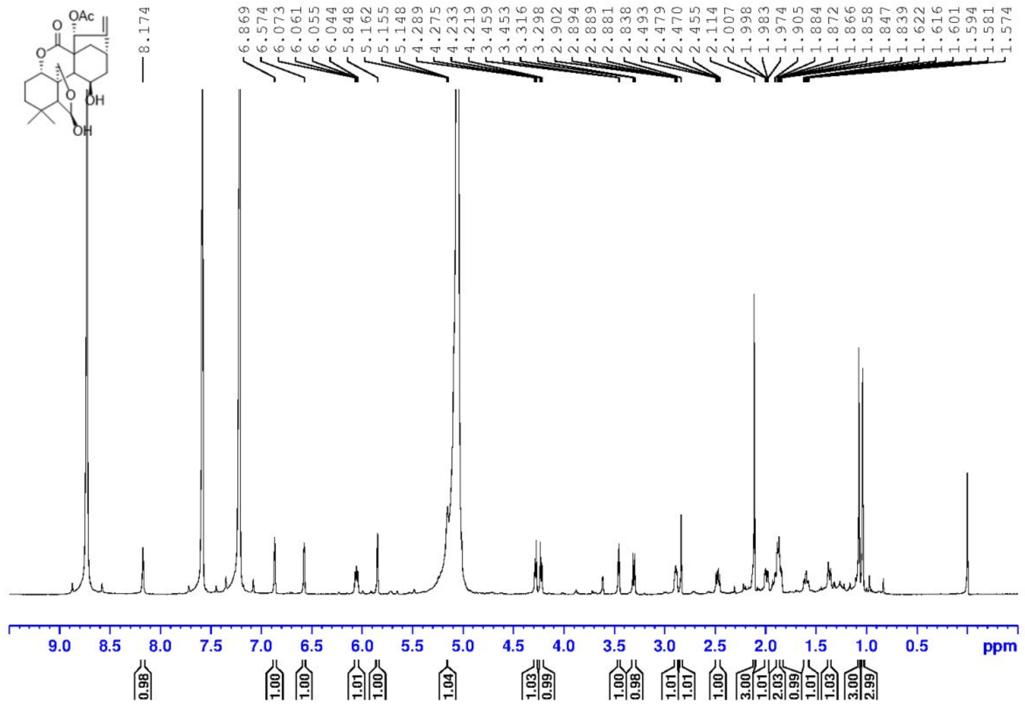

Figure S32. The ^{1}H NMR spectrum of compound 4

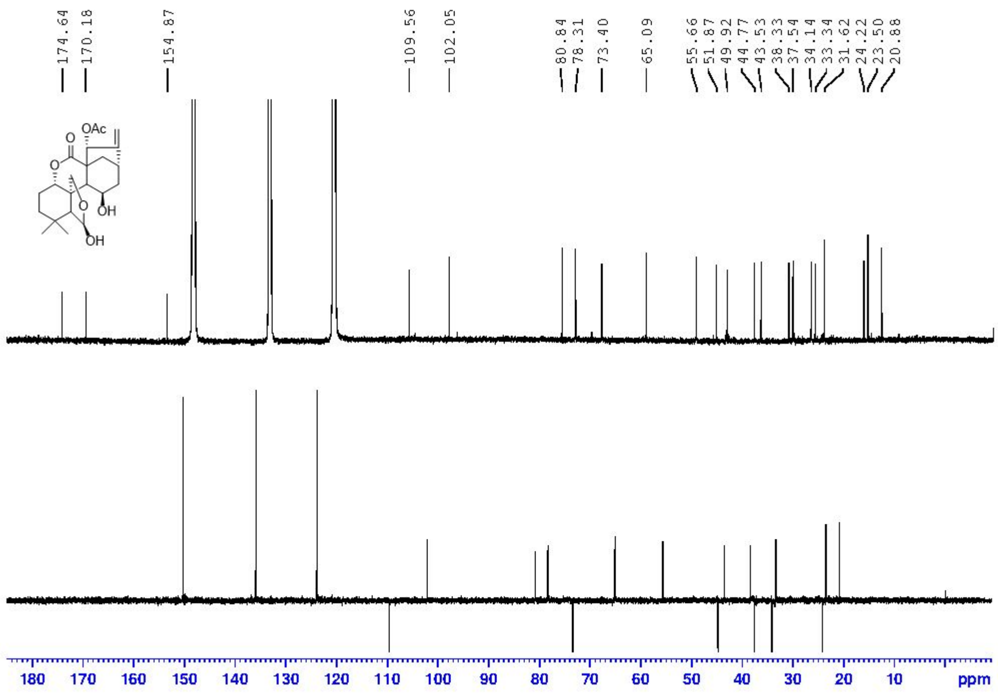

Figure S33. The ^{13}C and DEPT 135 NMR spectra of compound 4

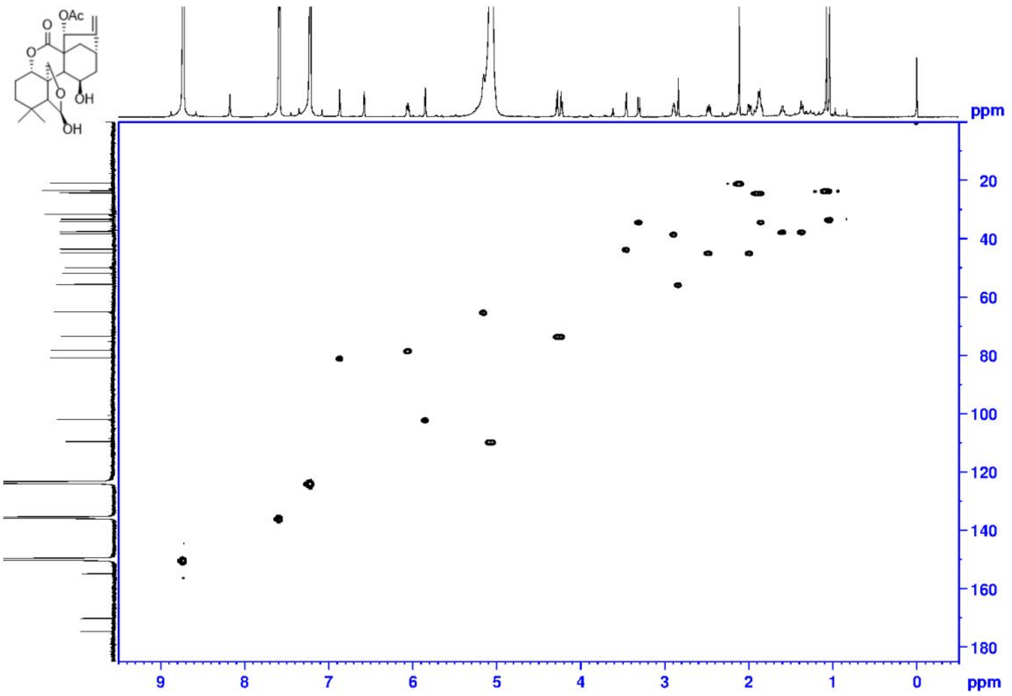

Figure S34. The HSQC spectrum of compound 4

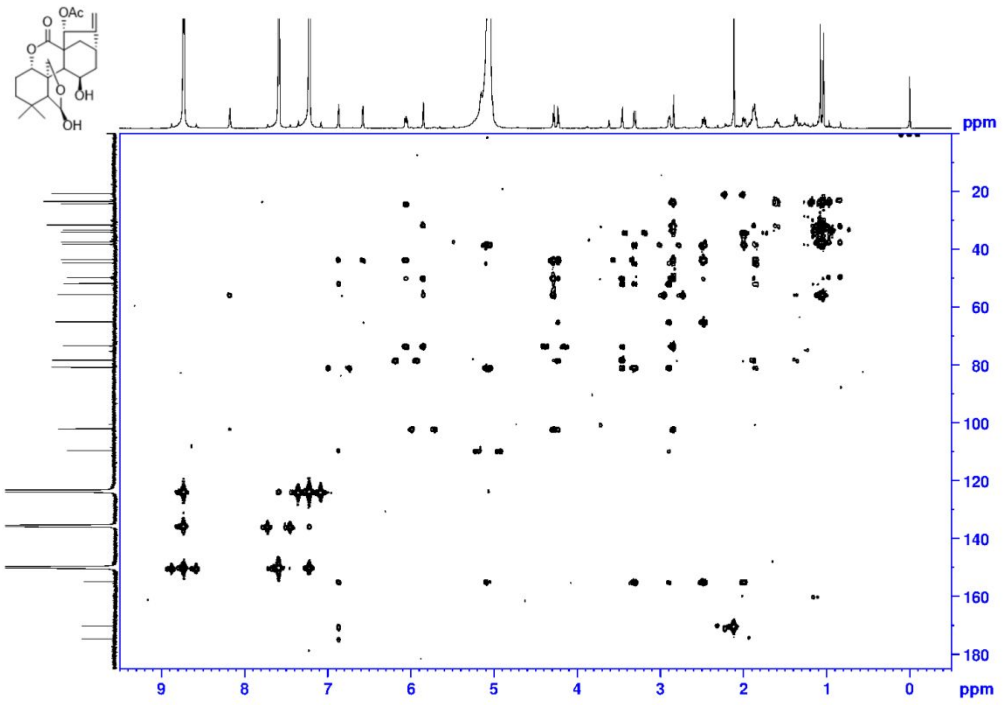

Figure S35. The HMBC spectrum of compound 4

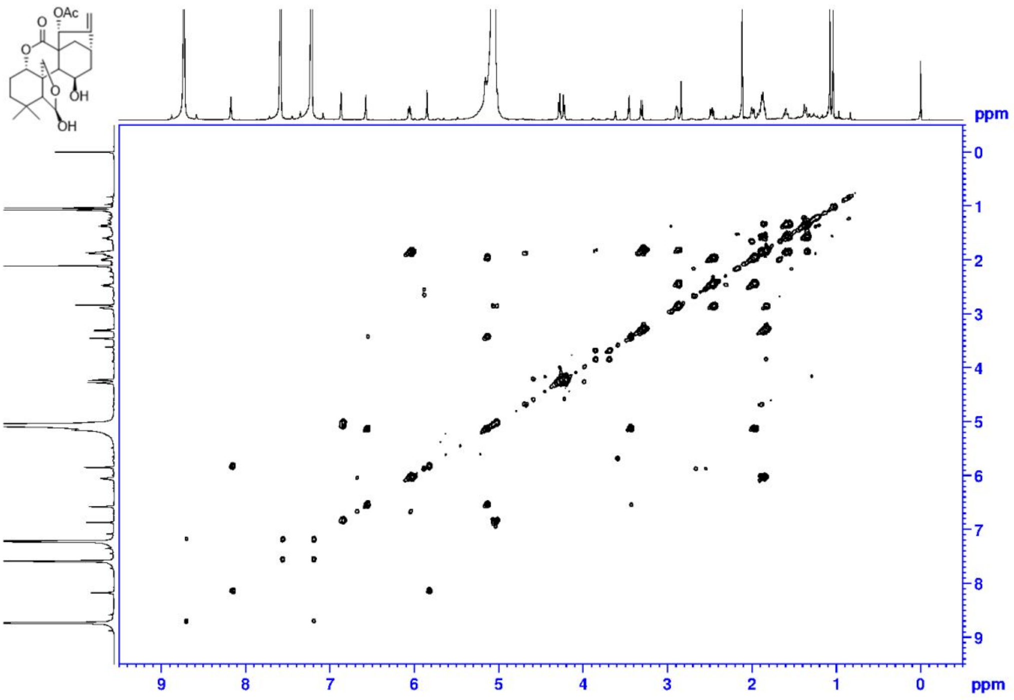

Figure S36. The ^{1}H-^{1}H COSY spectrum of compound 4

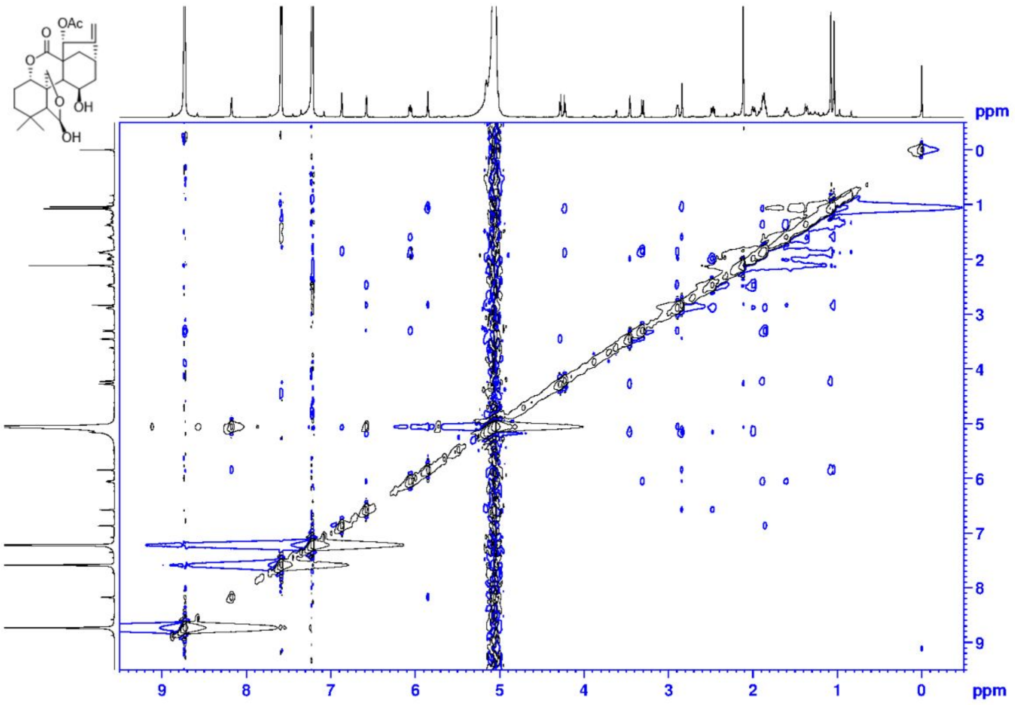

Figure S37. The NOESY spectrum of compound 4

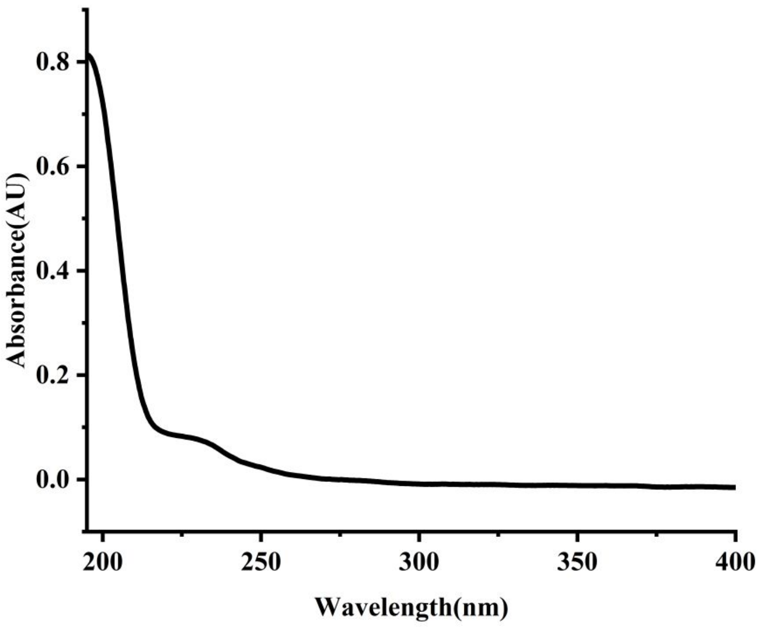

Figure S38. The UV spectrum of compound 4

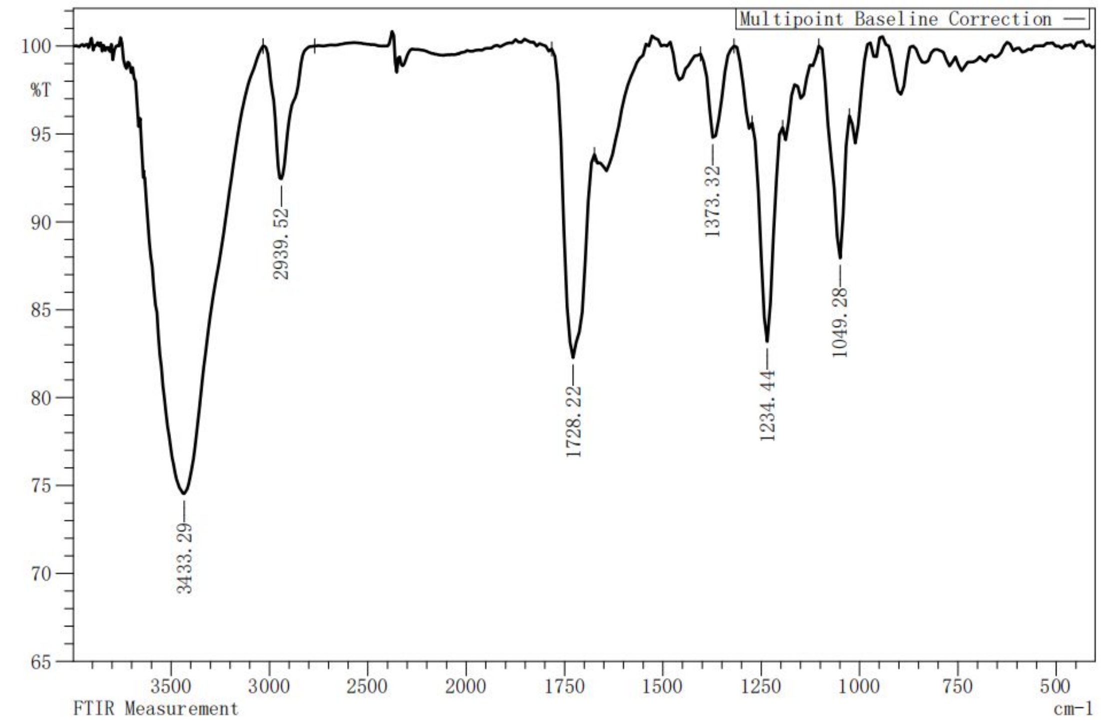

Figure S39. The IR spectrum of compound 4

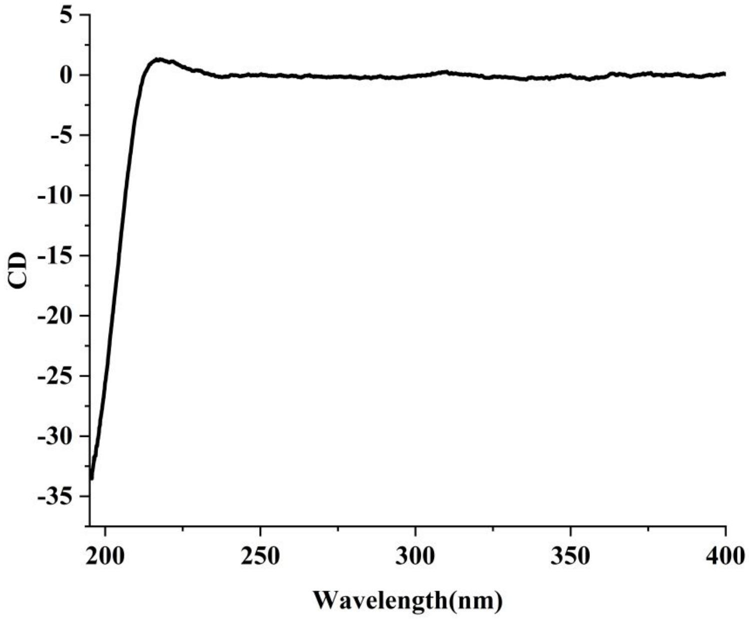

Figure S40. The CD spectrum of compound 4

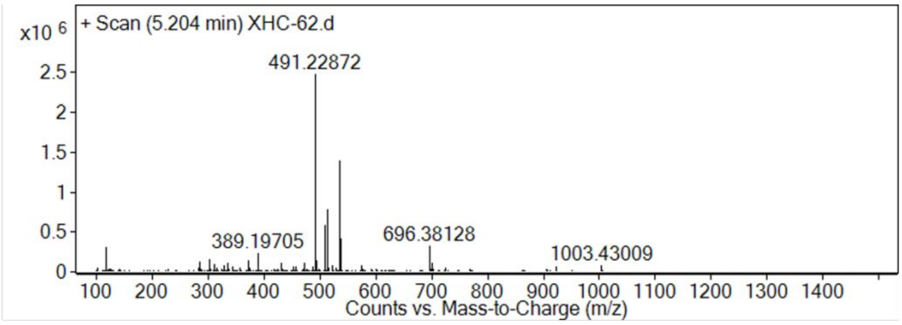

Formula	Score	Mass	Mass (MFG)	*m/z* (Calc)	Diff (ppm)	Ion Formula	*m/z*
$C_{26}H_{34}O_9$	97.33	490.2214	490.2203	491.2276	-2.33	$C_{26}H_{35}O_9$	491.2287

Figure S41. The HRESIMS spectrum of compound 5

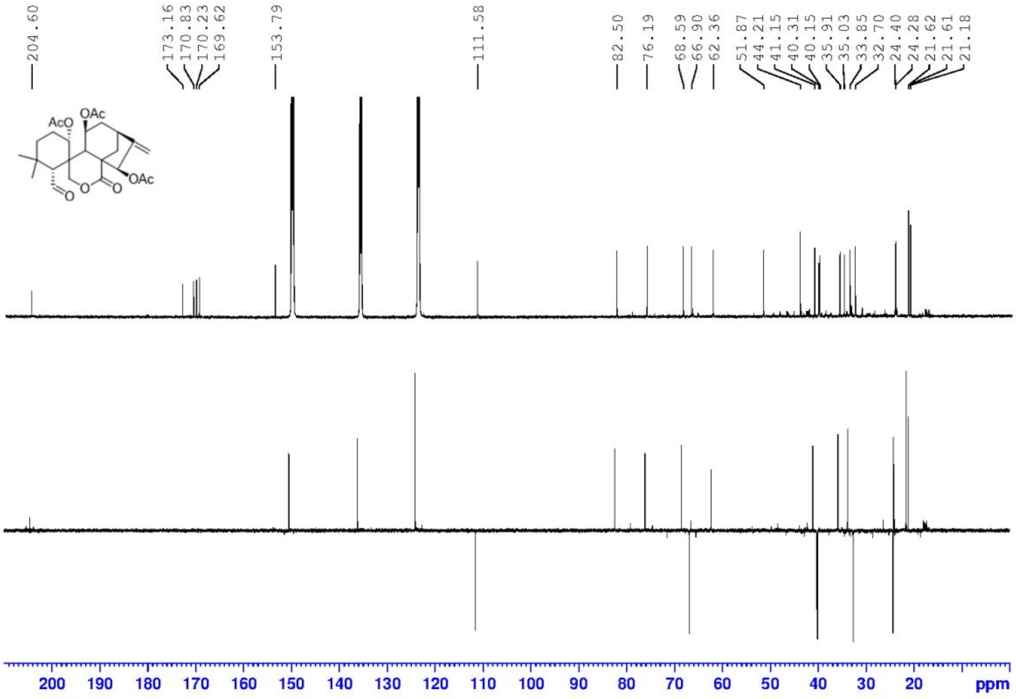

Figure S42. The ¹H NMR spectrum of compound 5

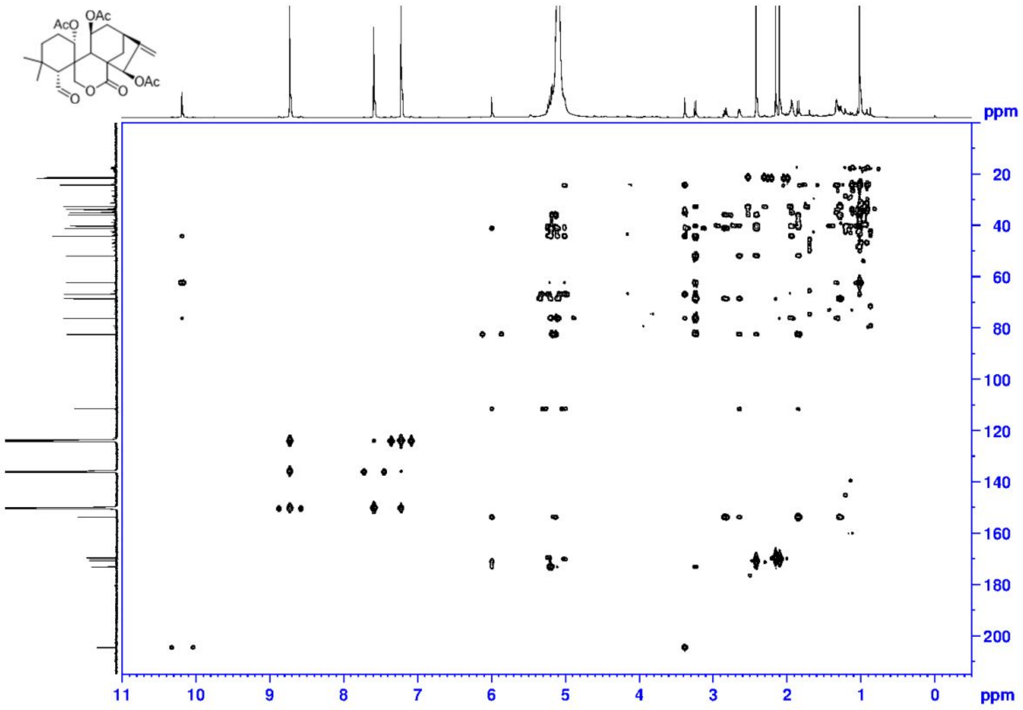

Figure S43. The ^{13}C and DEPT 135 NMR spectra of compound 5

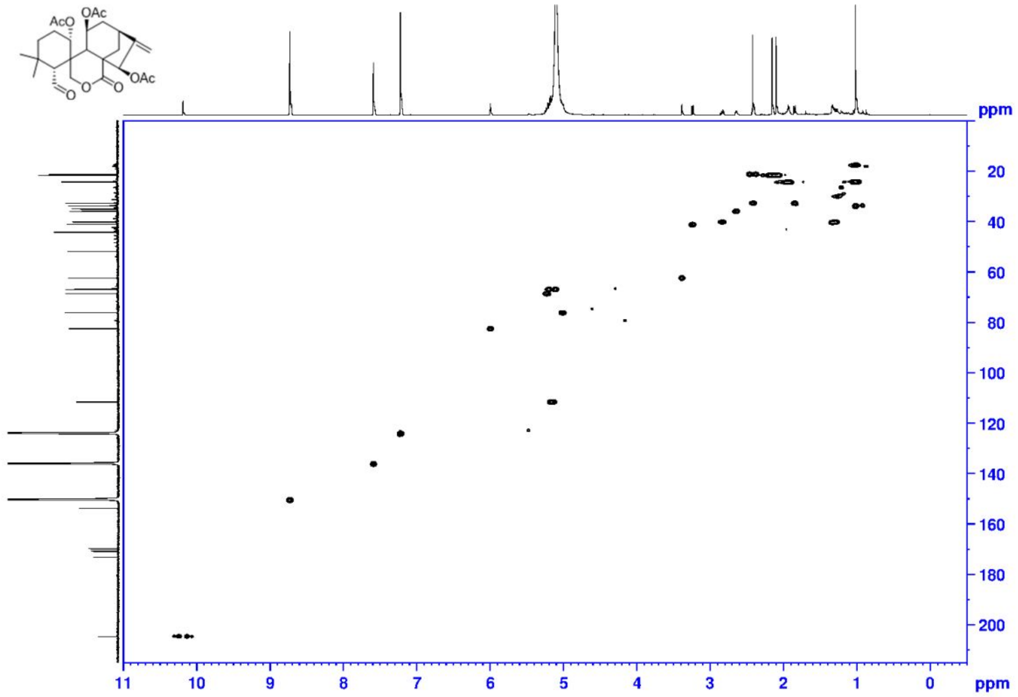

Figure S44. The HSQC spectrum of compound 5

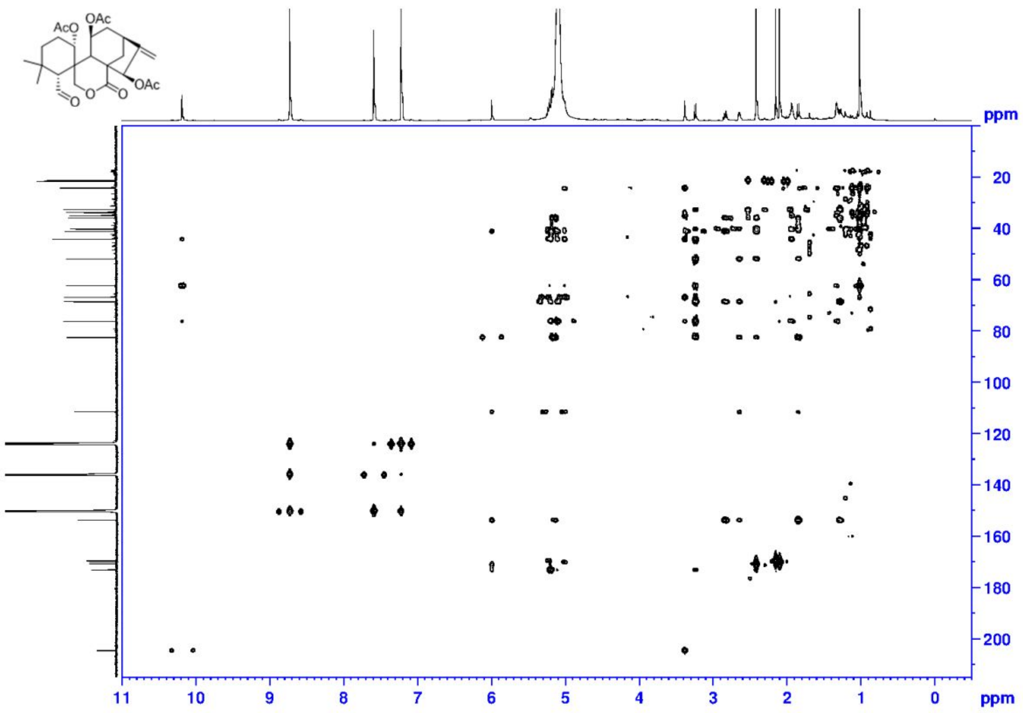

Figure S45. The HMBC spectrum of compound 5

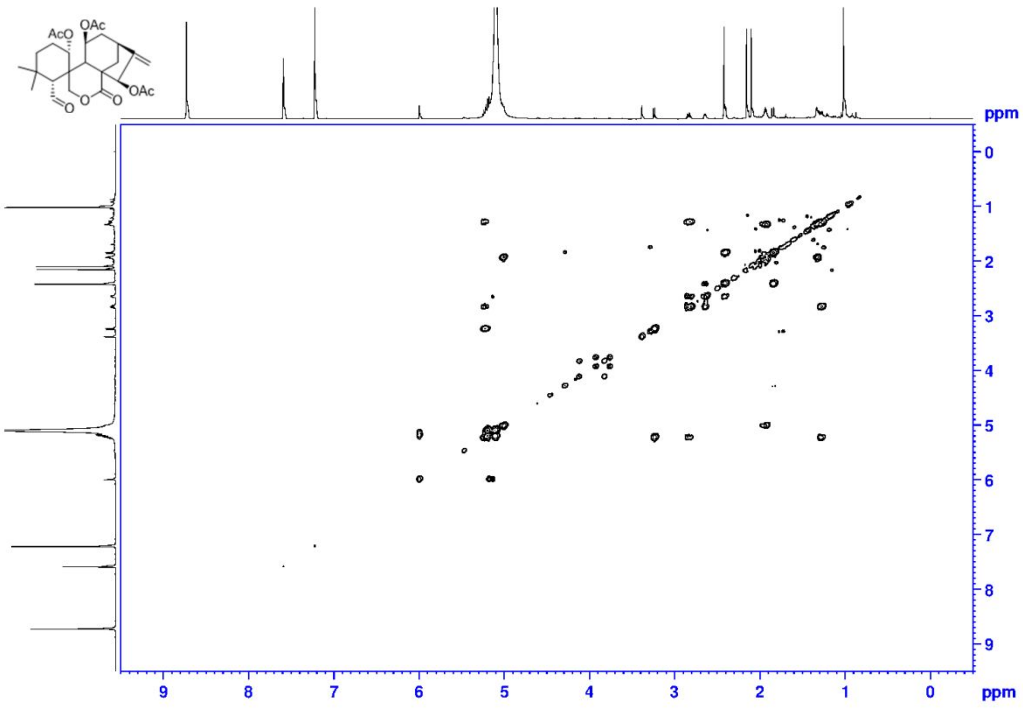

Figure S46. The ^{1}H-^{1}H COSY spectrum of compound 5

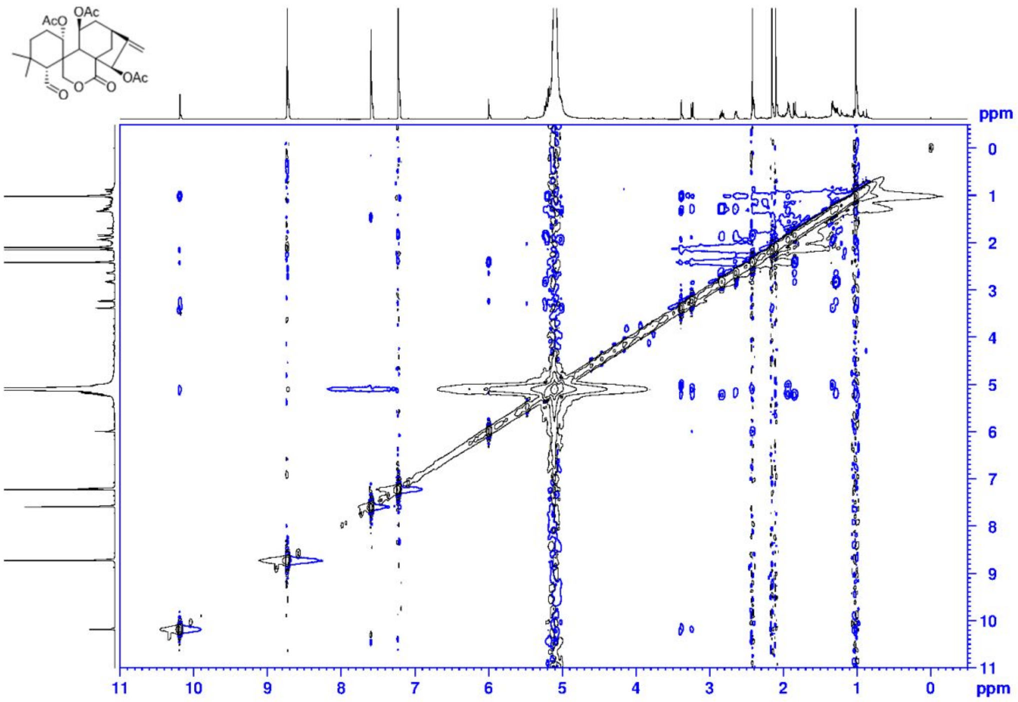

Figure S47. The NOESY spectrum of compound 5

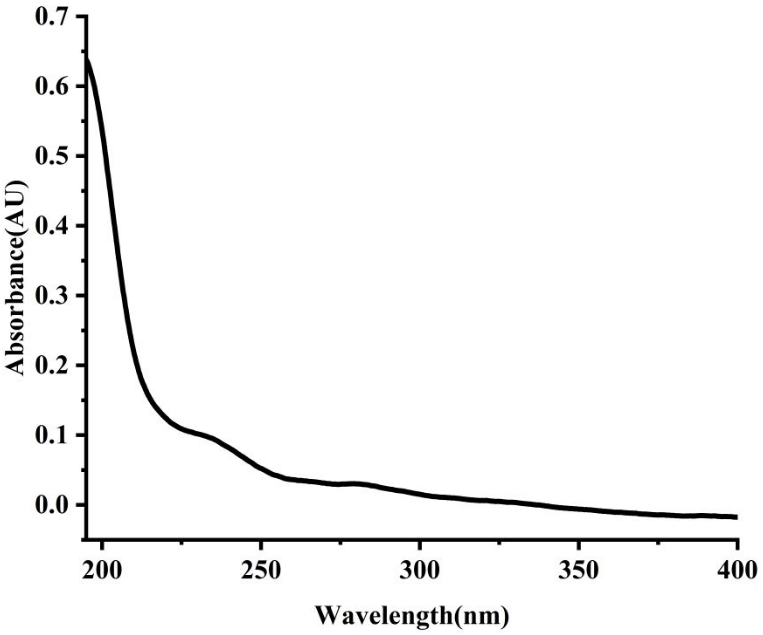

Figure S48. The UV spectrum of compound 5

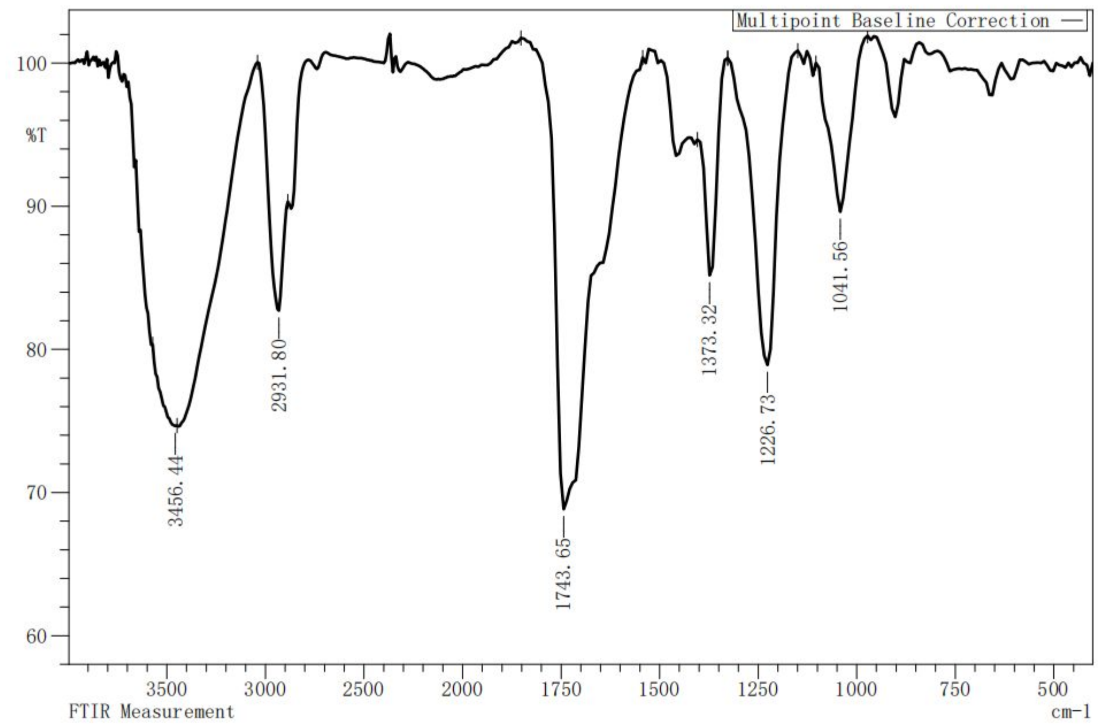

Figure S49. The IR spectrum of compound 5

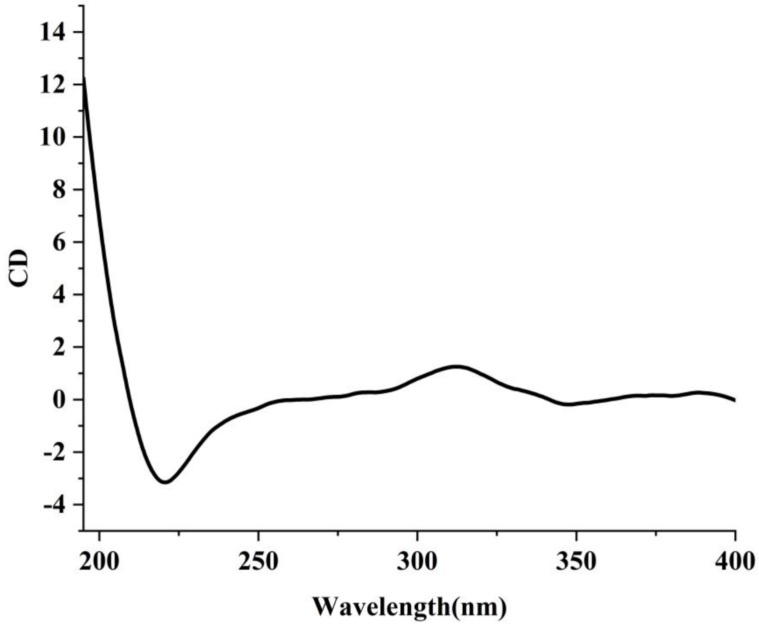

Figure S50. The CD spectrum of compound 5

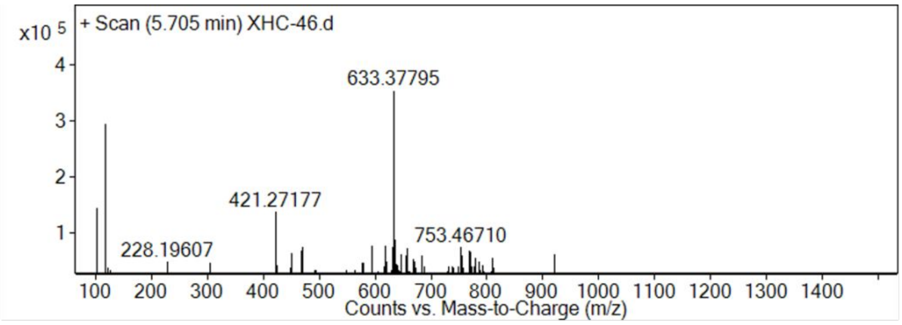

Formula	Score	Mass	Mass (MFG)	*m/z* (Calc)	Diff (ppm)	Ion Formula	*m/z*
$C_{39}H_{52}O_7$	99.48	632.3706	632.3713	633.3786	0.92	$C_{39}H_{53}O_7$	633.3780

Figure S51. The HRESIMS spectrum of compound 6

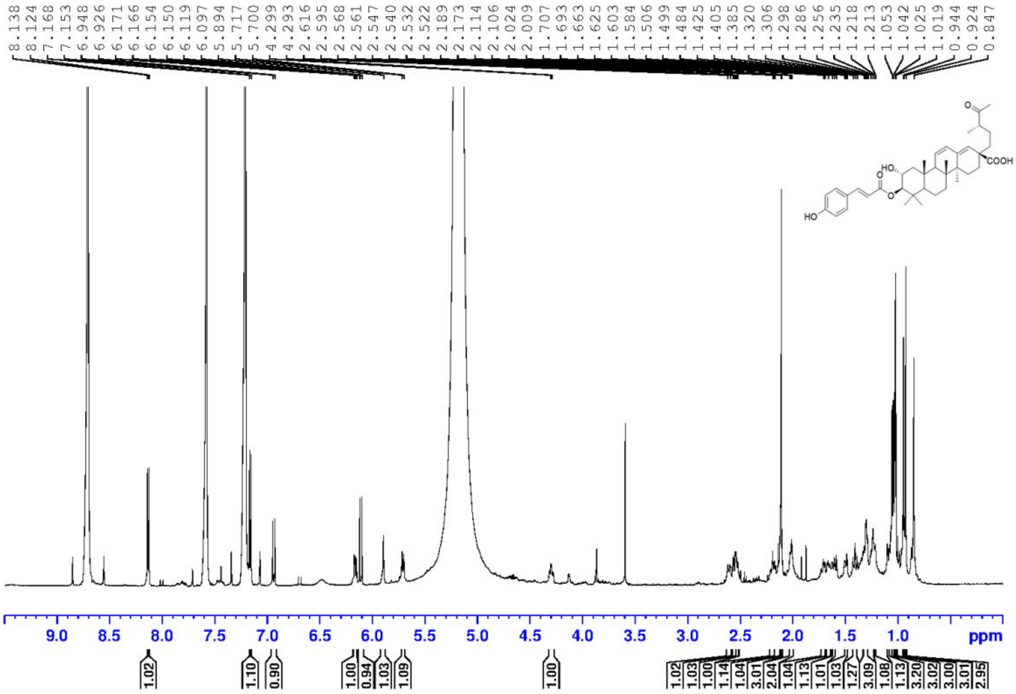

Figure S52. The ^{1}H NMR spectrum of compound 6

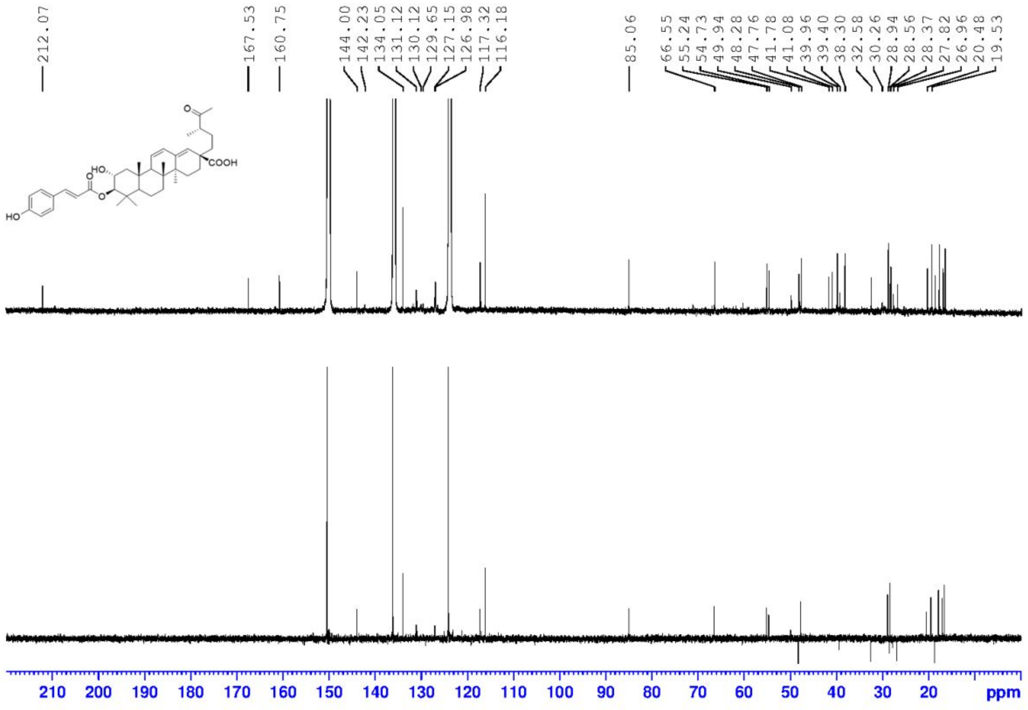

Figure S53. The ^{13}C and DEPT 135 NMR spectra of compound 6

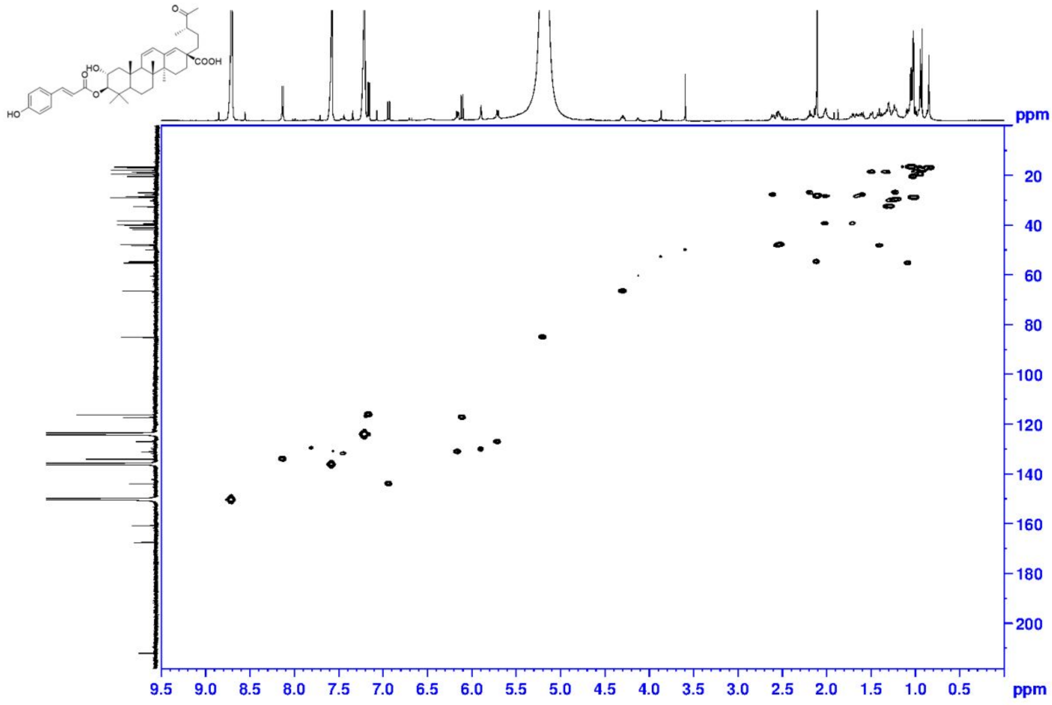

Figure S54. The HSQC spectrum of compound 6

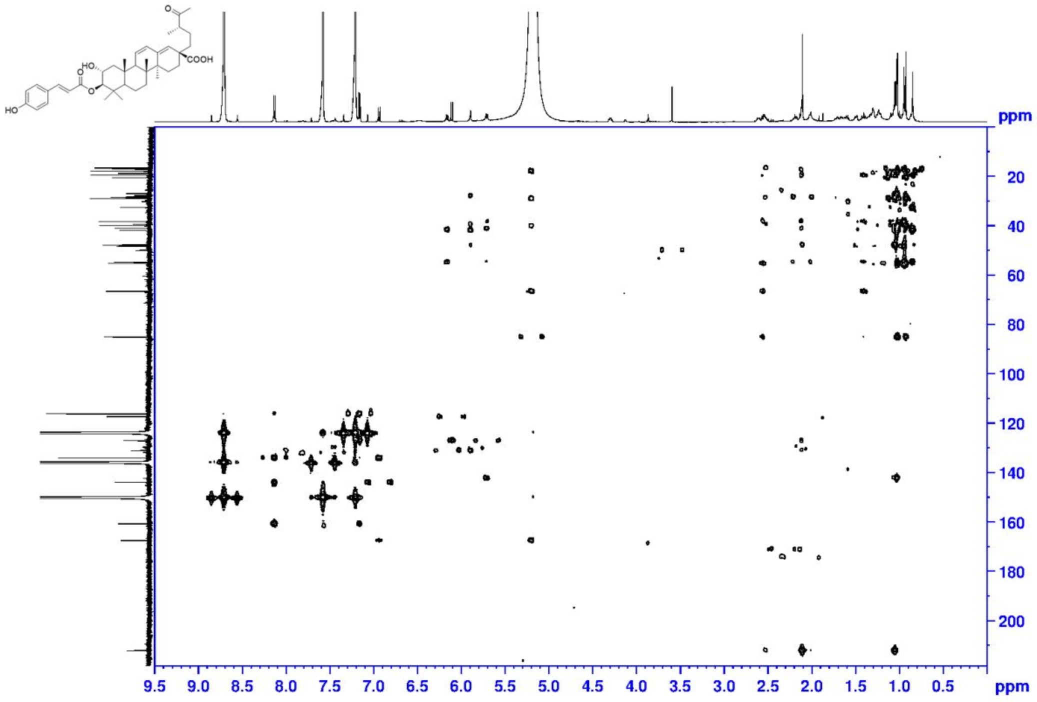

Figure S55. The HMBC spectrum of compound 6

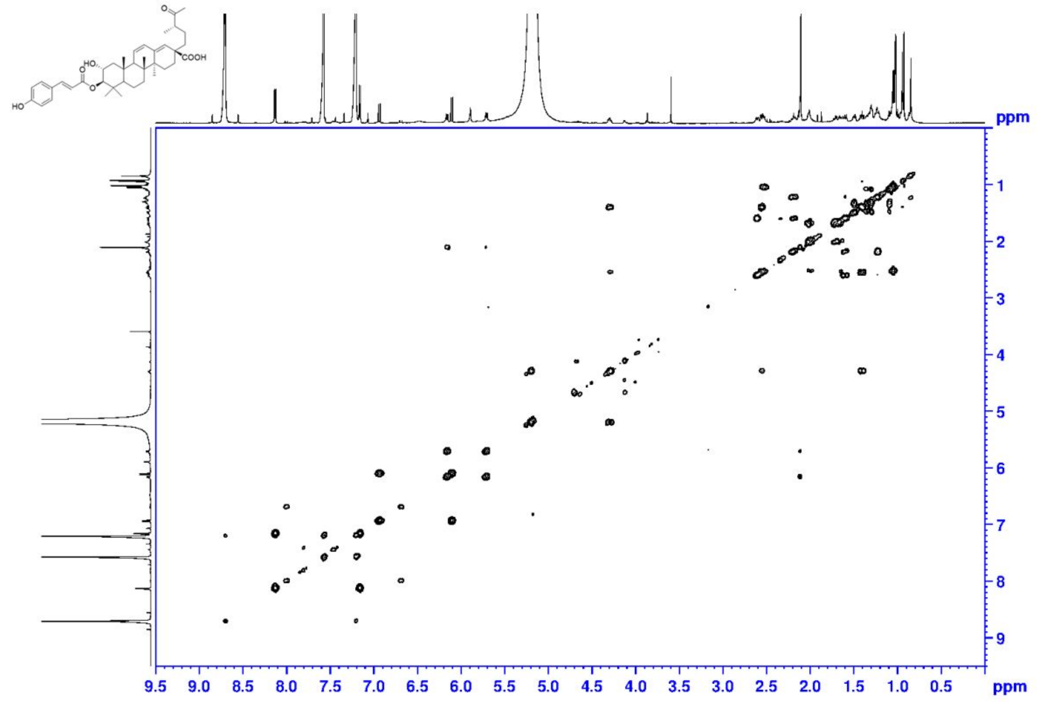

Figure S56. The ^{1}H-^{1}H COSY spectrum of compound 6

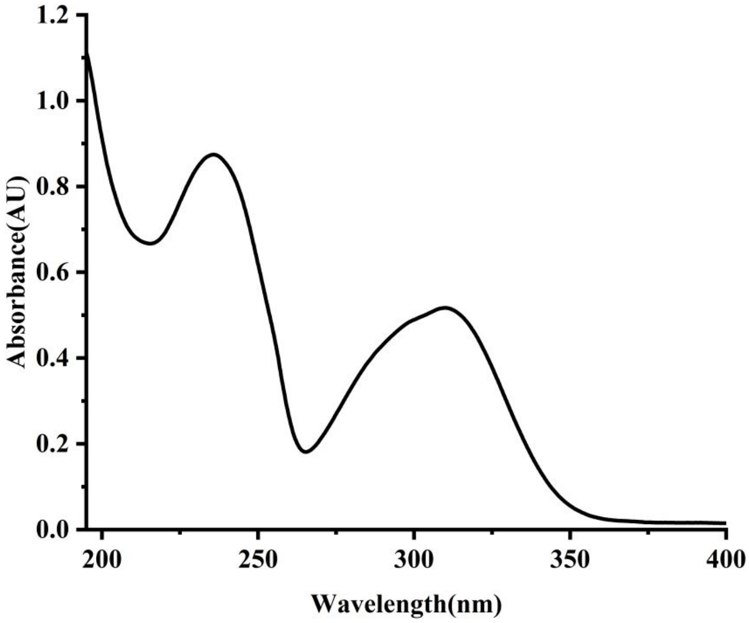

Figure S57. The UV spectrum of compound 6

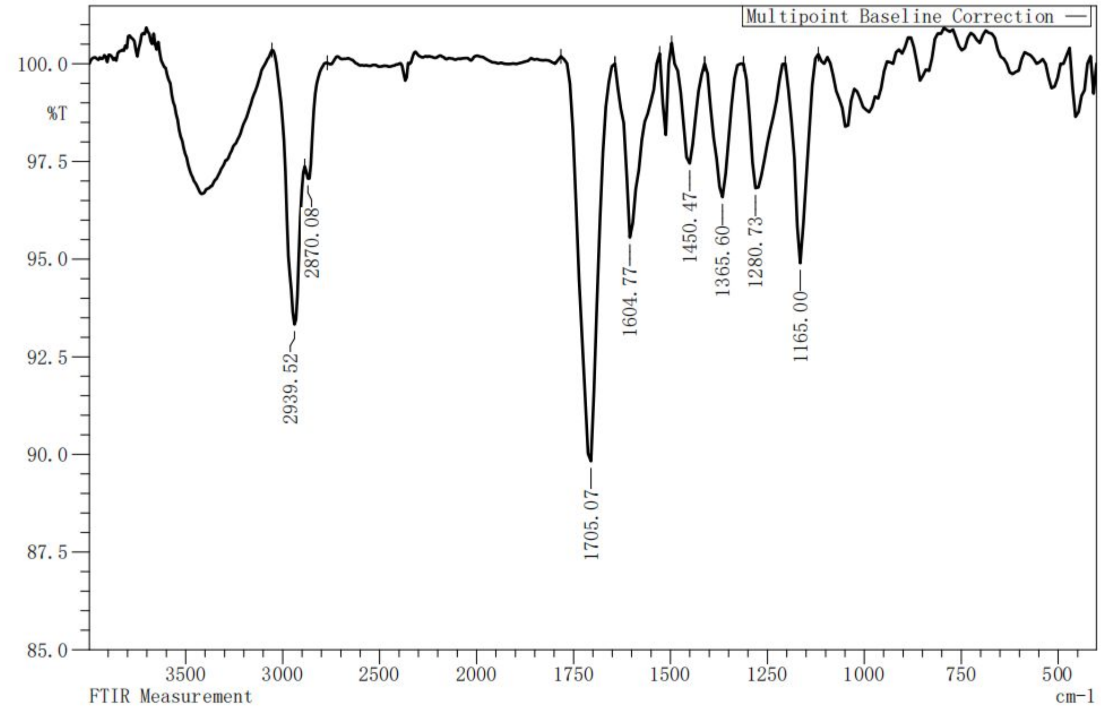

Figure S58. The IR spectrum of compound 6

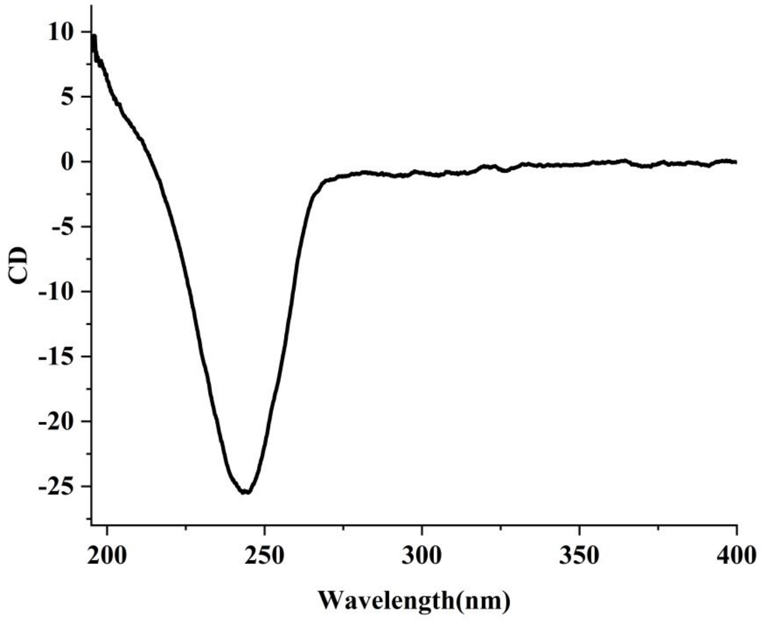

Figure S59. The CD spectrum of compound 6

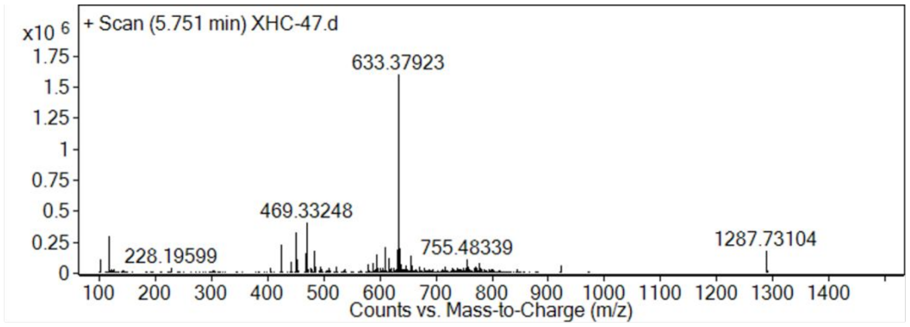

Formula	Score	Mass	Mass (MFG)	*m/z* (Calc)	Diff (ppm)	Ion Formula	*m/z*
$C_{39}H_{52}O_7$	99.41	632.3719	632.3713	633.3786	-0.98	$C_{39}H_{53}O_7$	633.3792

Figure S60. The HRESIMS spectrum of compound 7

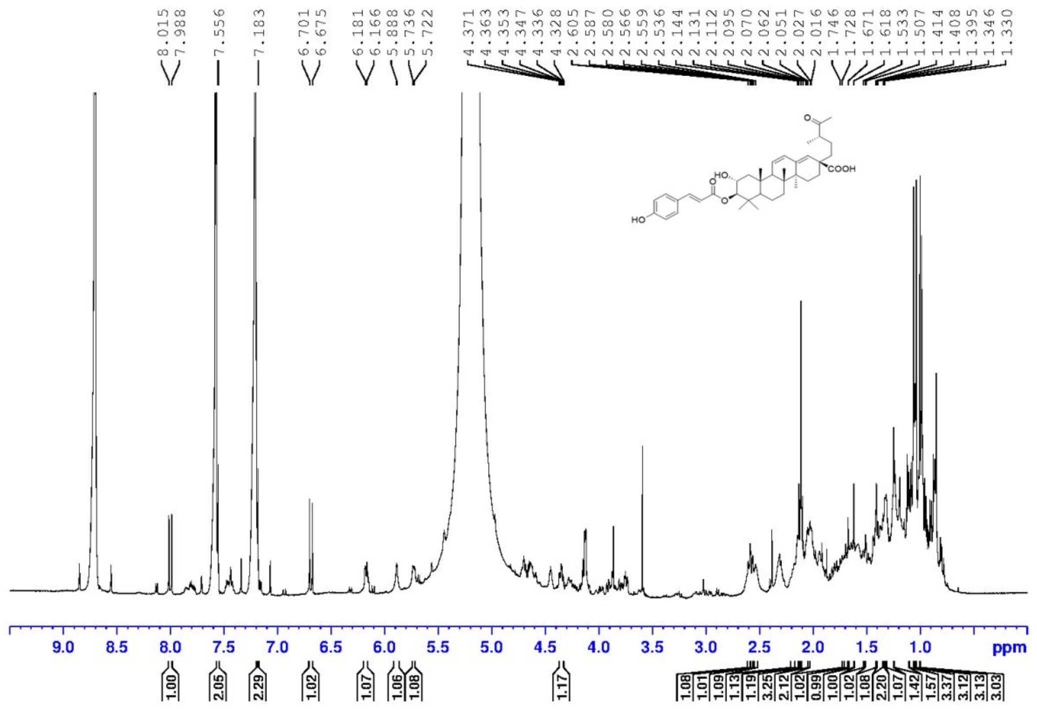

Figure S61. The ^{1}H NMR spectrum of compound 7

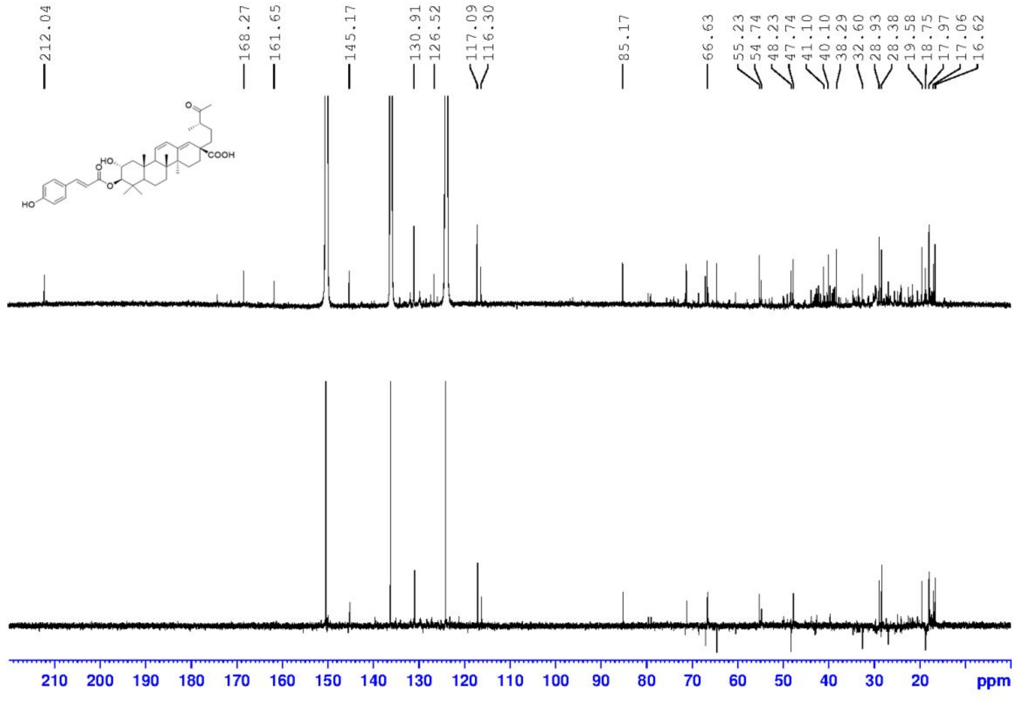

Figure S62. The ^{13}C and DEPT 135 NMR spectra of compound 7

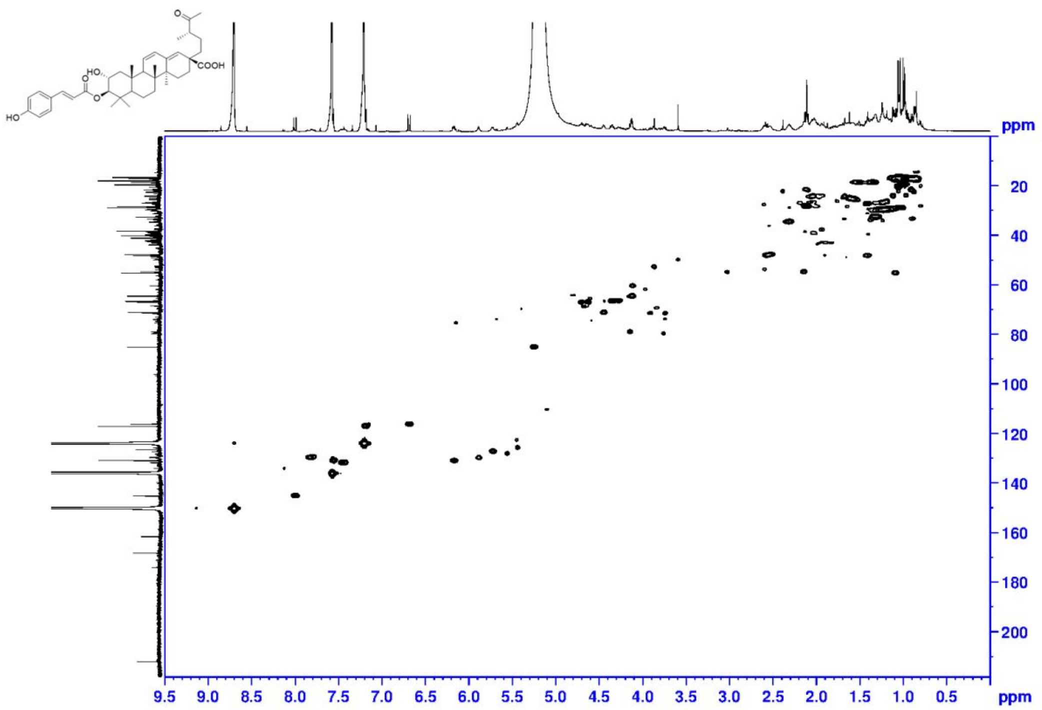

Figure S63. The HSQC spectrum of compound 7

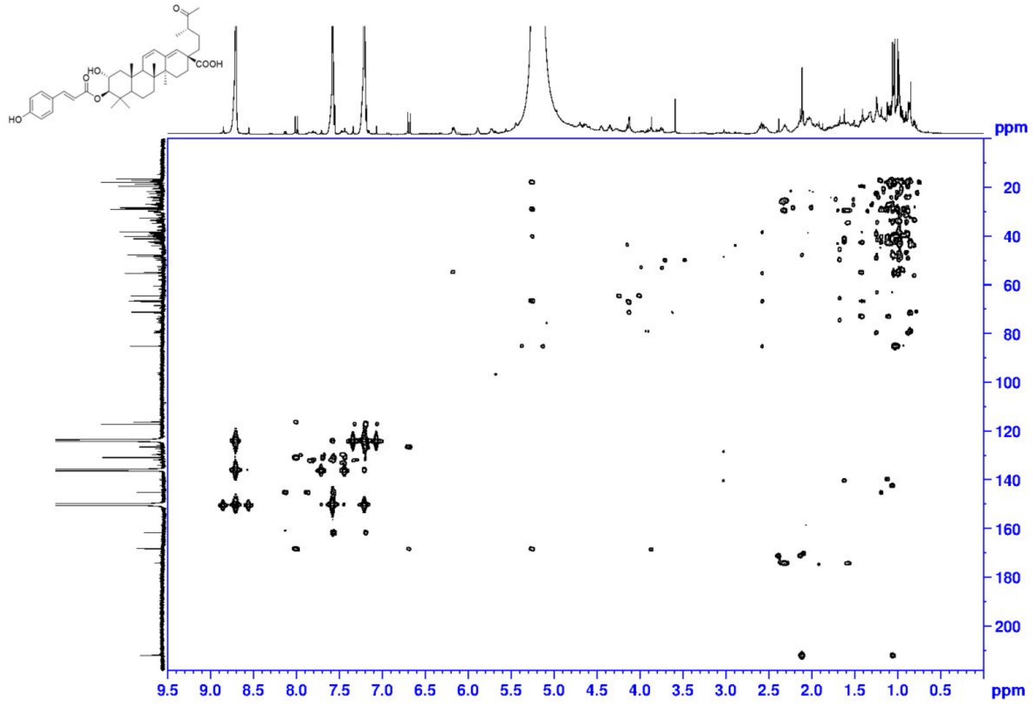

Figure S64. The HMBC spectrum of compound 7

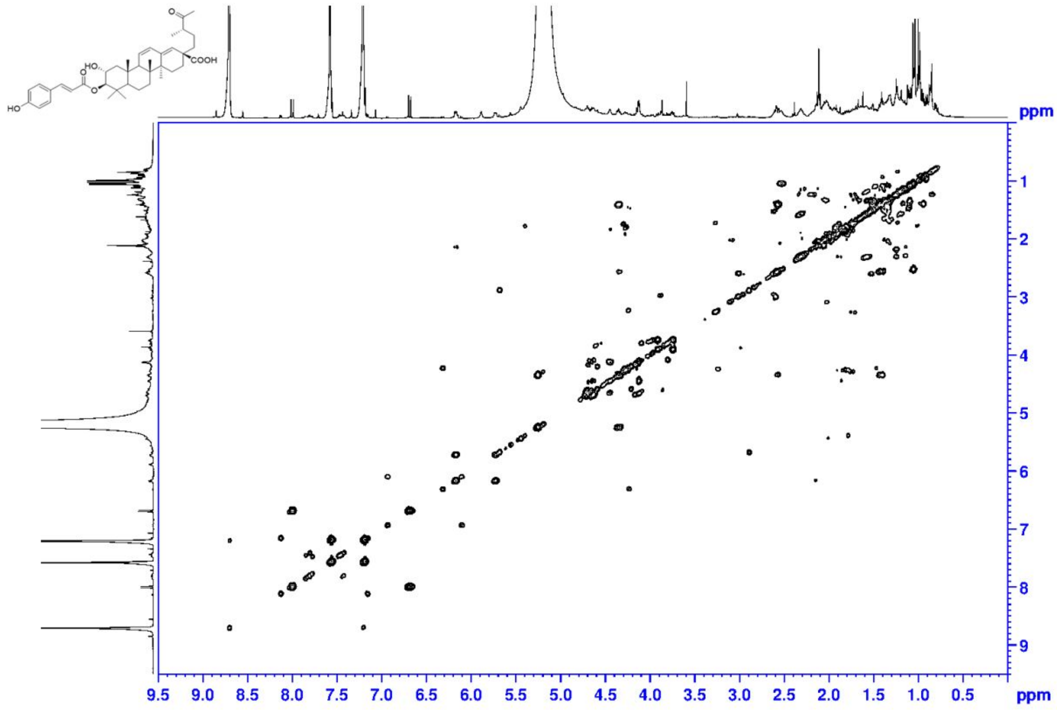

Figure S65. The ^{1}H-^{1}H COSY spectrum of compound 7

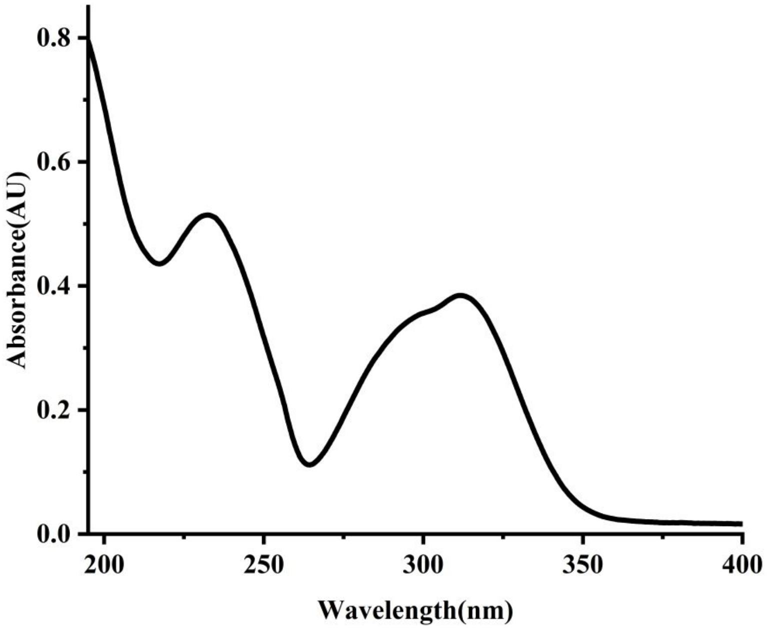

Figure S66. The UV spectrum of compound 7

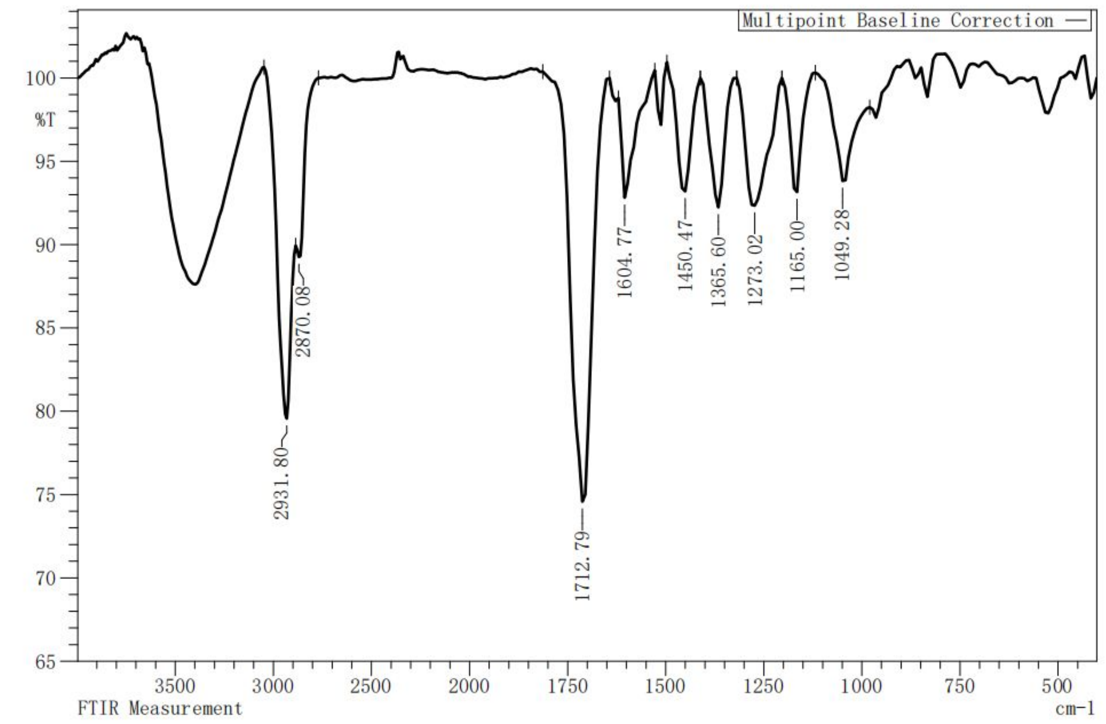

Figure S67. The IR spectrum of compound 7

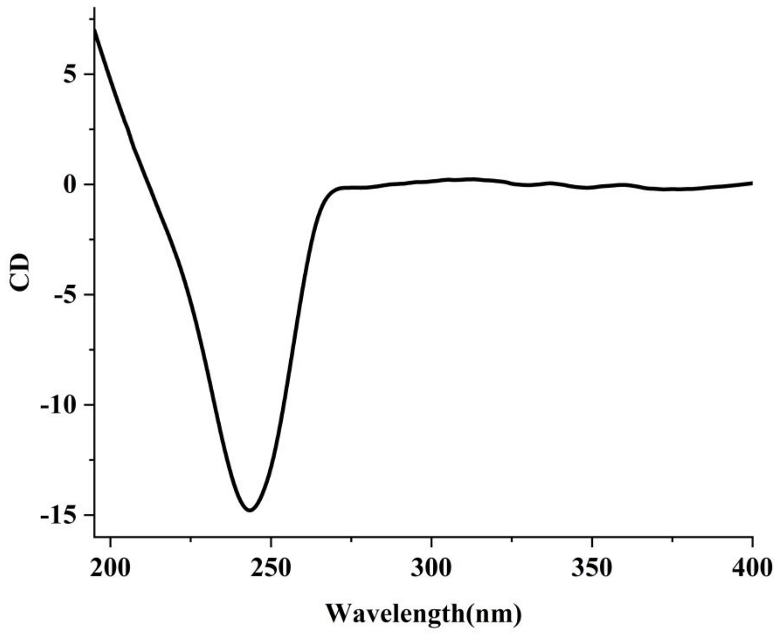

Figure S68. The CD spectrum of compound 7

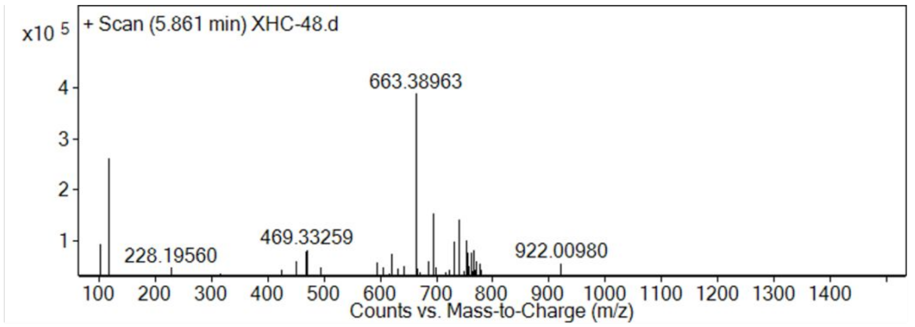

Formula	Score	Mass	Mass (MFG)	*m/z* (Calc)	Diff (ppm)	Ion Formula	*m/z*
$C_{40}H_{54}O_8$	99.7	662.3823	662.3819	663.3891	-0.69	$C_{40}H_{55}O_8$	663.3896

Figure S69. The HRESIMS spectrum of compound 8

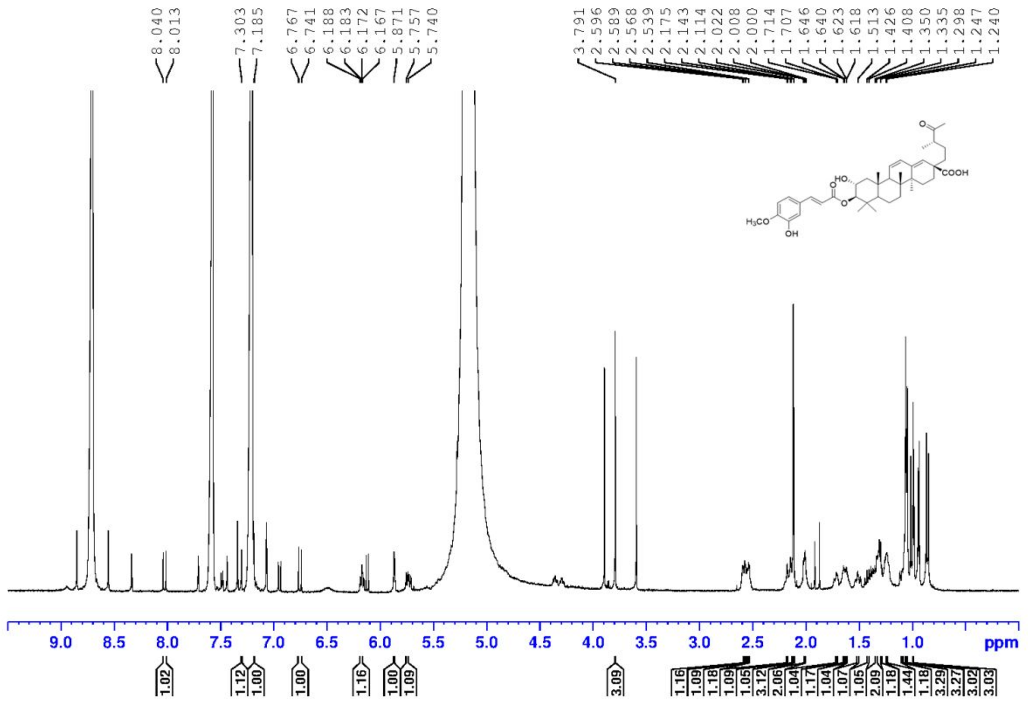

Figure S70. The ^{1}H NMR spectrum of compound 8

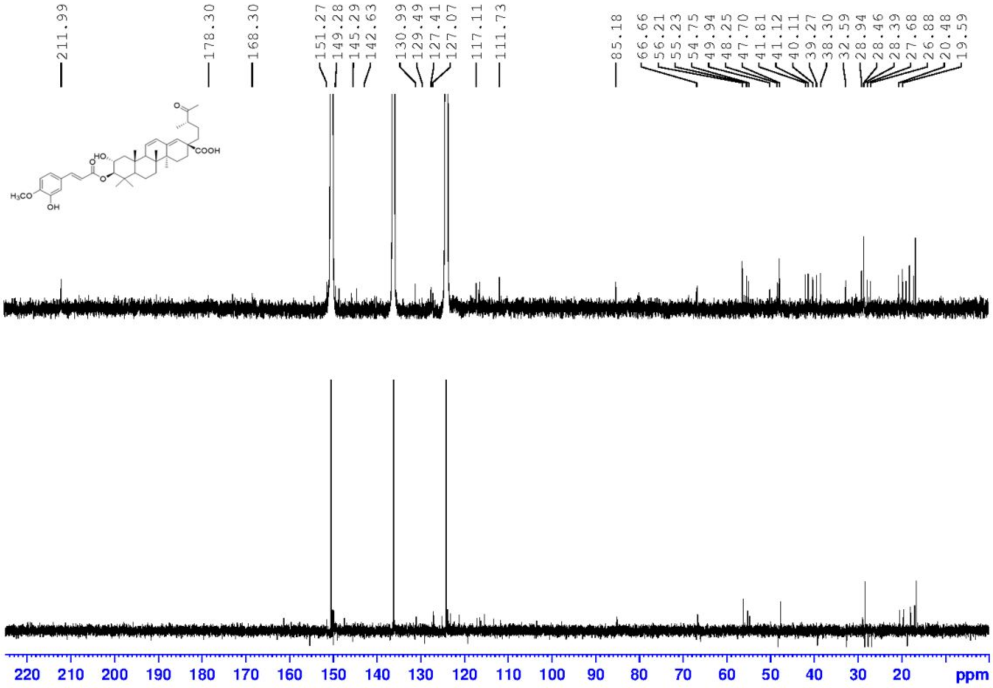

Figure S71. The ^{13}C and DEPT 135 NMR spectra of compound 8

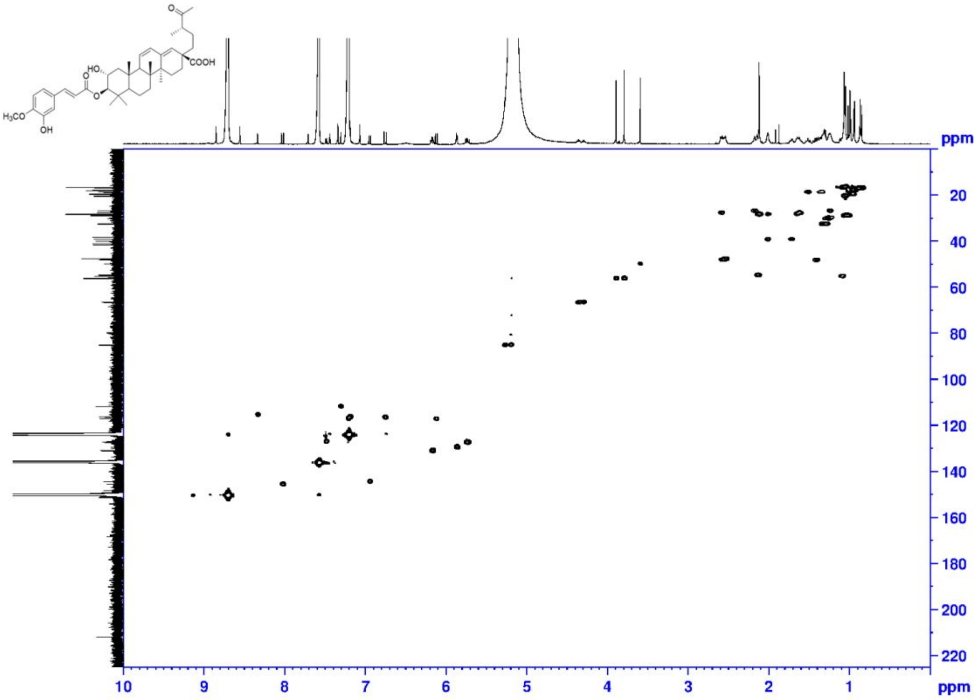

Figure S72. The HSQC spectrum of compound 8

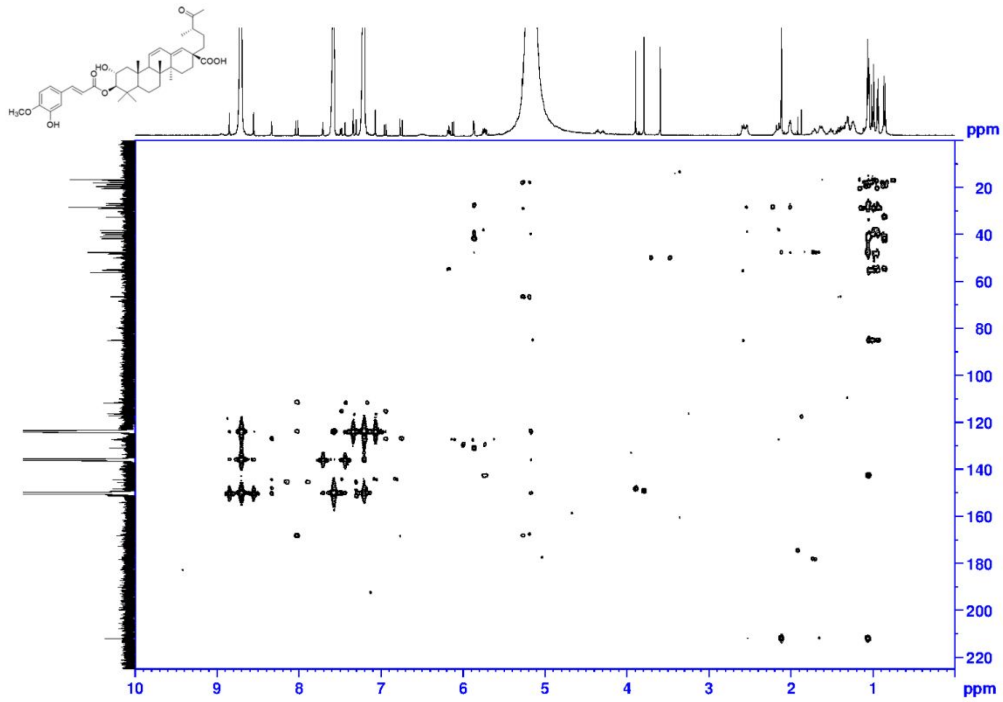

Figure S73. The HMBC spectrum of compound 8

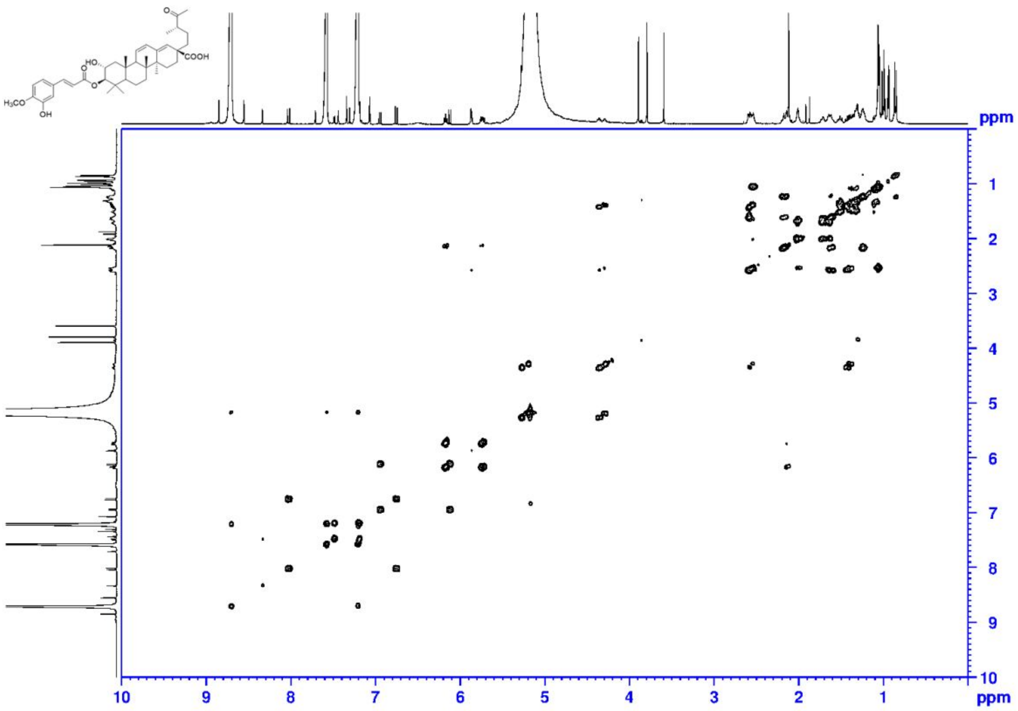

Figure S74. The ^{1}H-^{1}H COSY spectrum of compound 8

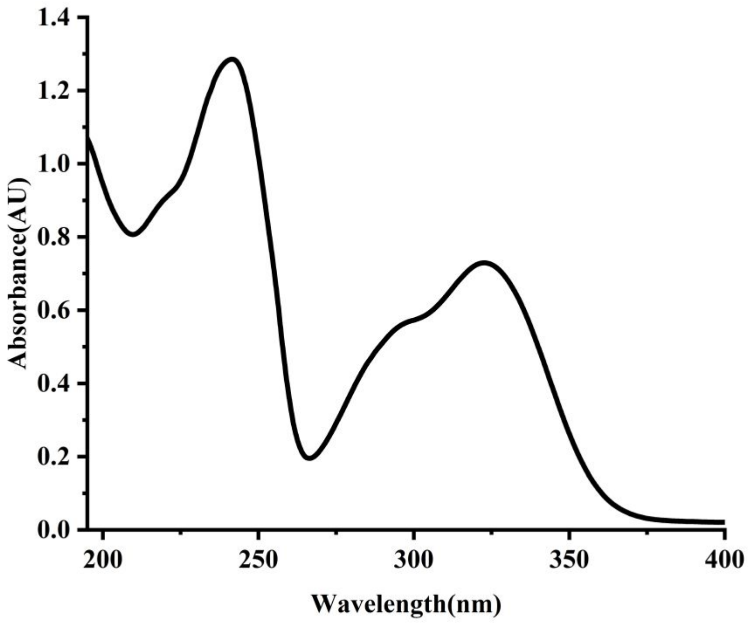

Figure S75. The UV spectrum of compound 8

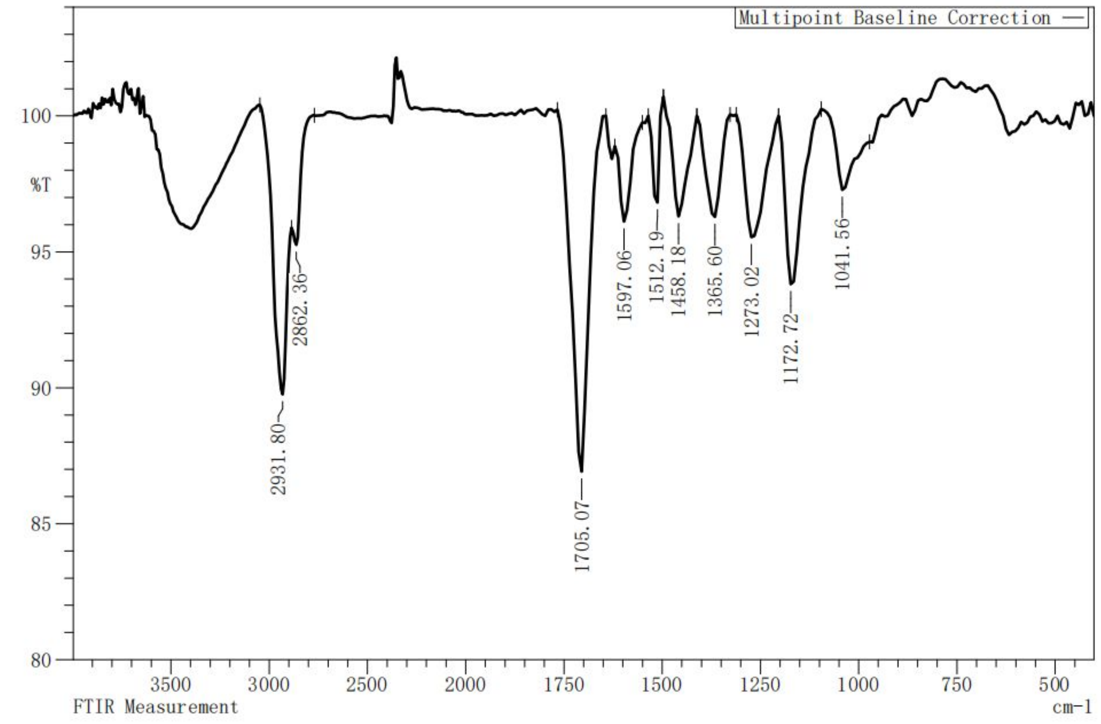

Figure S76. The IR spectrum of compound 8

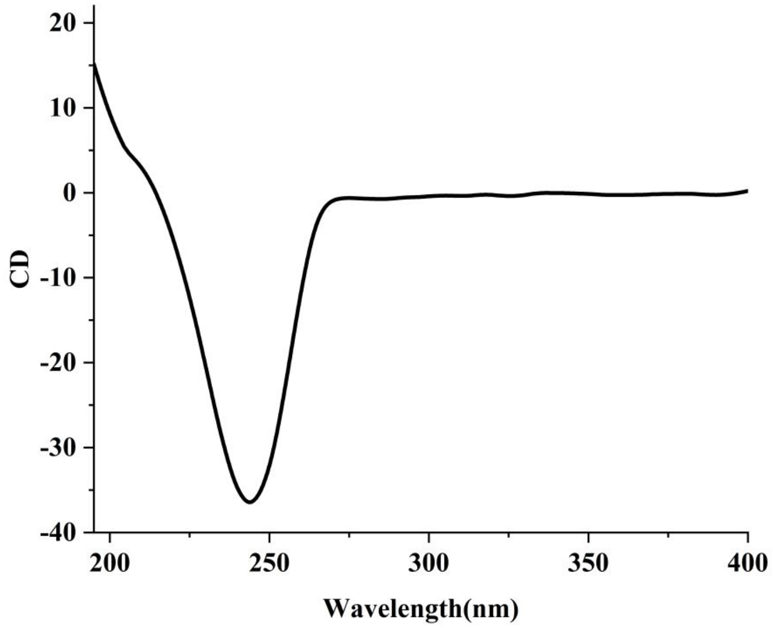

Figure S77. The CD spectrum of compound 8

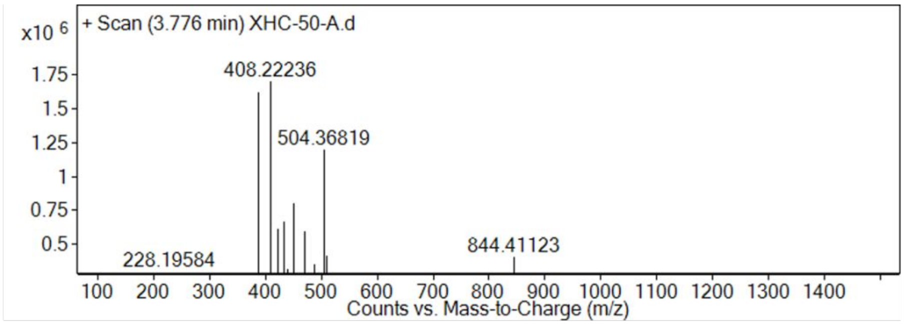

Formula	Score	Mass	Mass (MFG)	*m/z* (Calc)	Diff (ppm)	Ion Formula	*m/z*
$C_{30}H_{46}O_5$	99.95	486.3344	486.3345	504.3684	0.31	$C_{30}H_{50}NO_5$	504.3682

Figure S78. The HRESIMS spectrum of compound 9

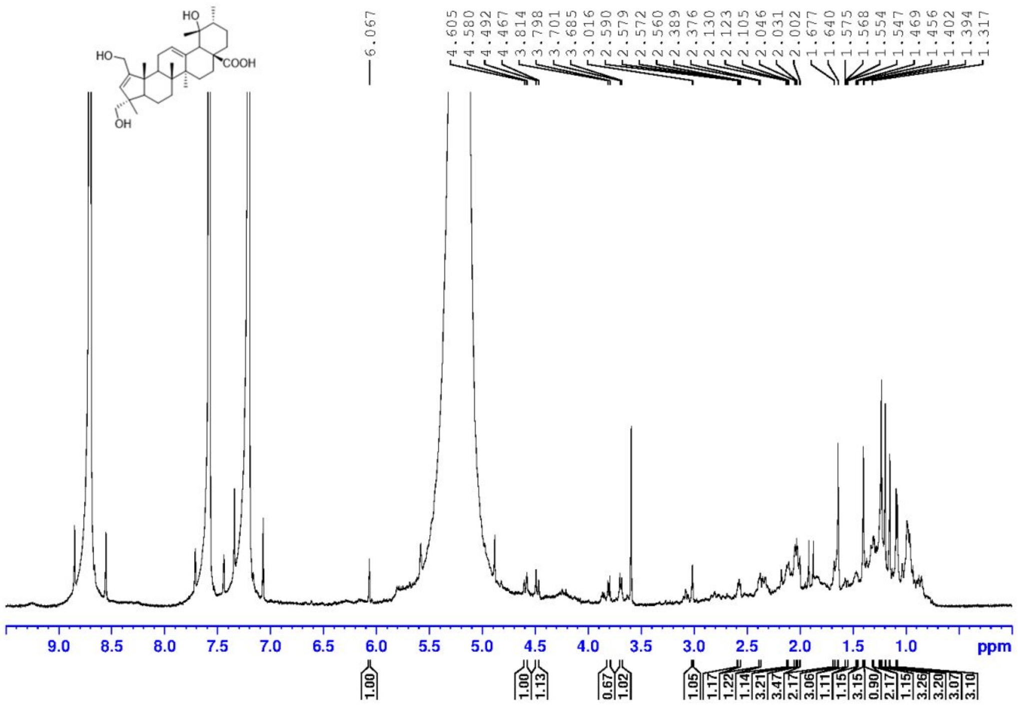

Figure S79. The ^{1}H NMR spectrum of compound 9

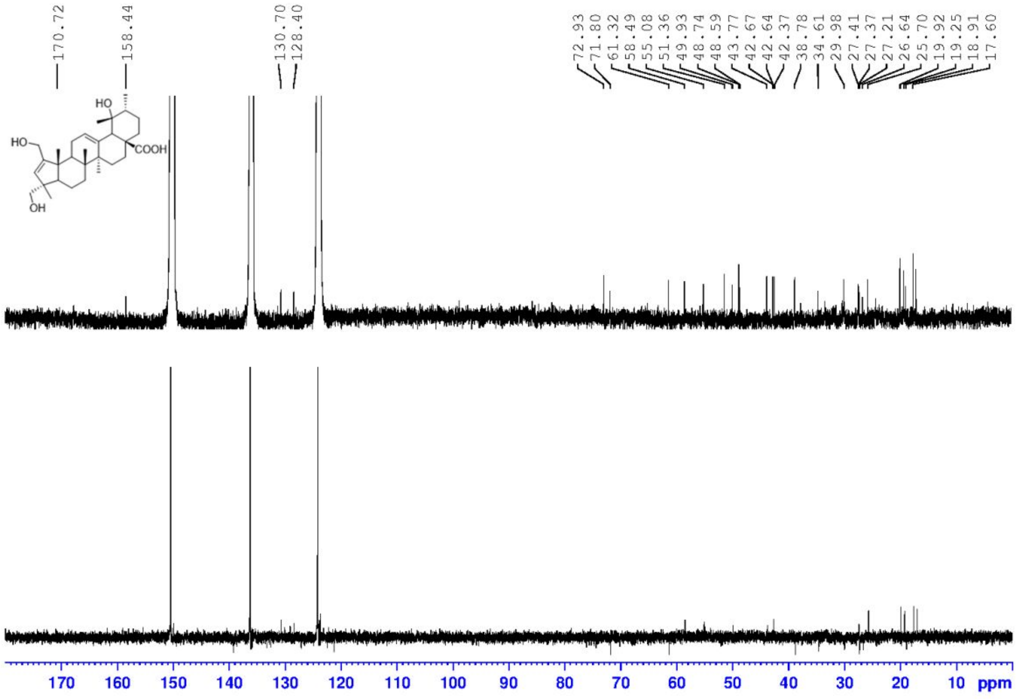

Figure S80. The ^{13}C and DEPT 135 NMR spectra of compound 9

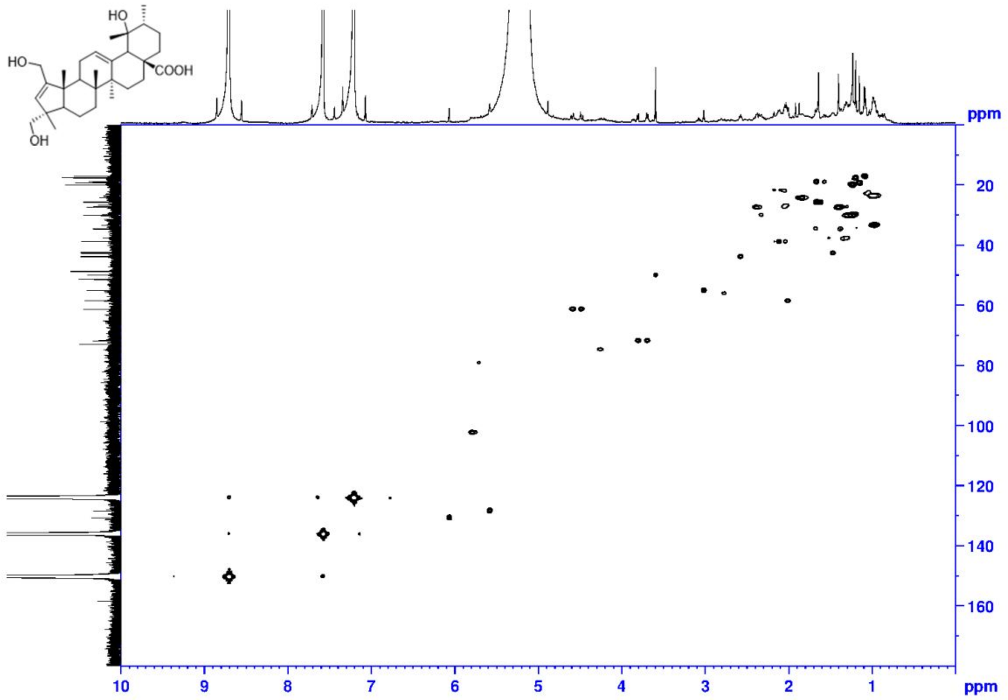

Figure S81. The HSQC spectrum of compound 9

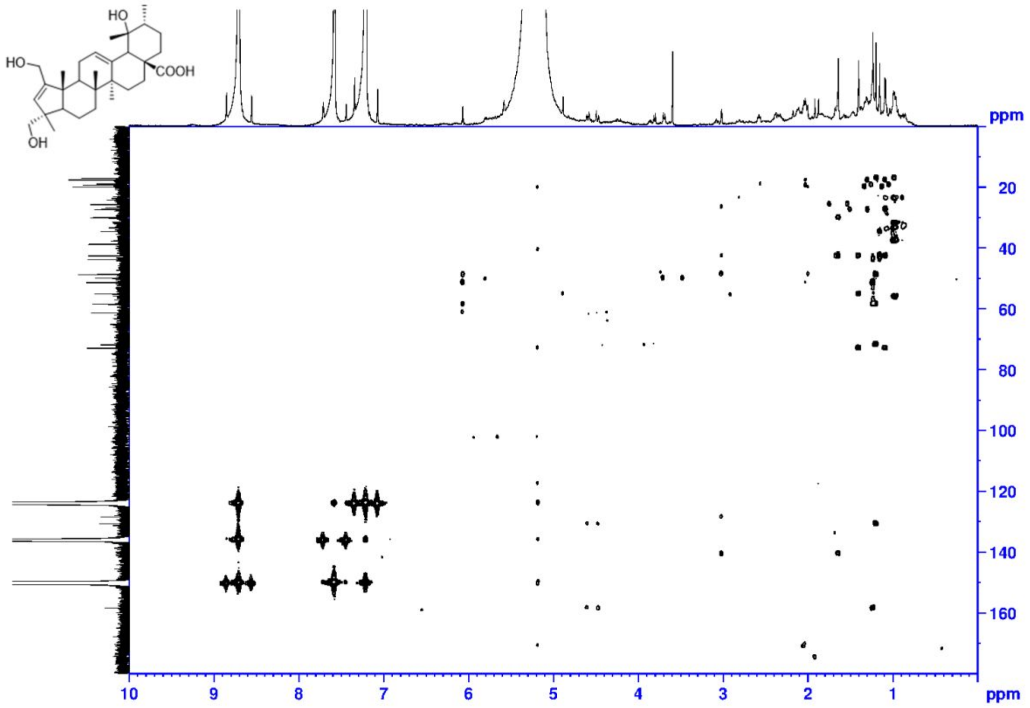

Figure S82. The HMBC spectrum of compound 9

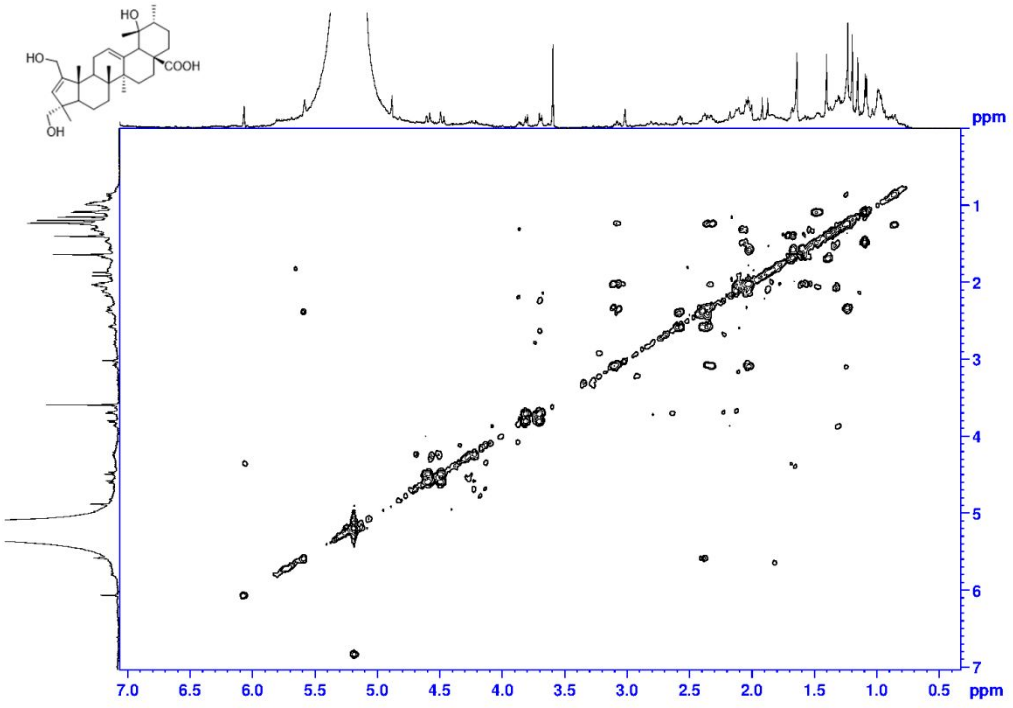

Figure S83. The ^{1}H-^{1}H COSY spectrum of compound 9

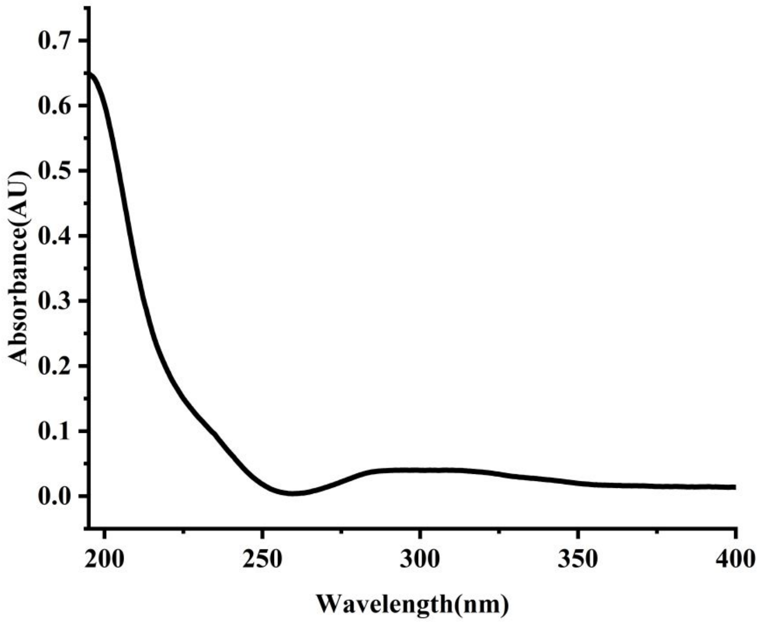

Figure S84. The UV spectrum of compound 9

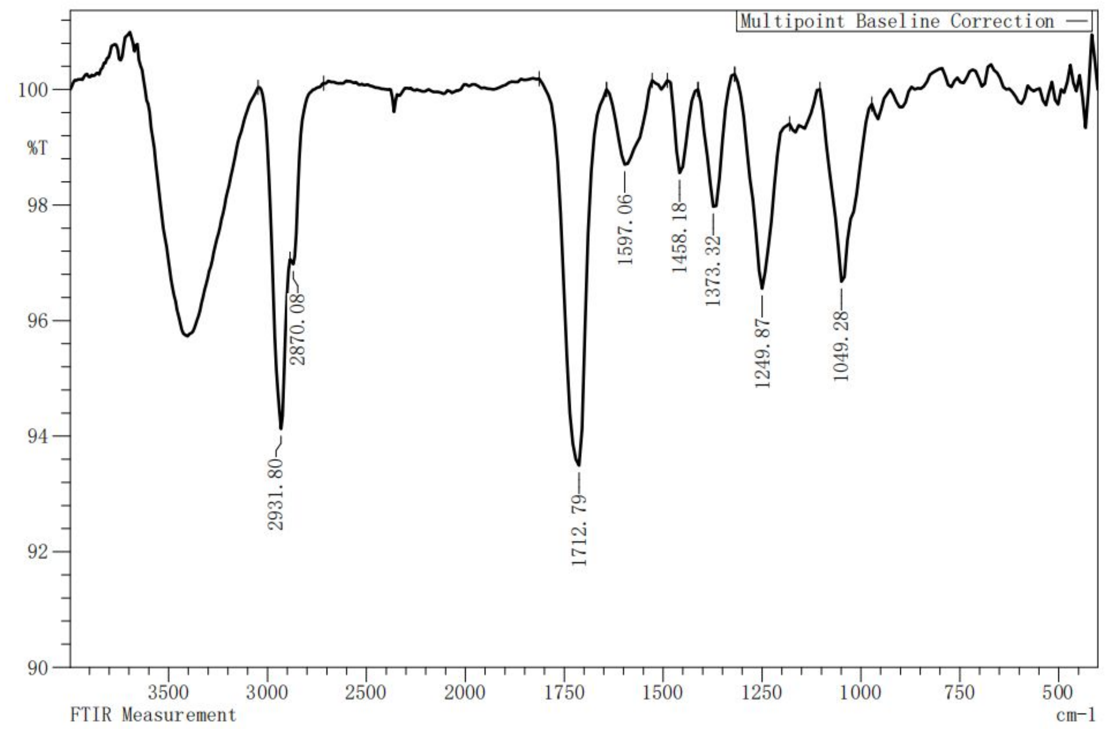

Figure S85. The IR spectrum of compound 9

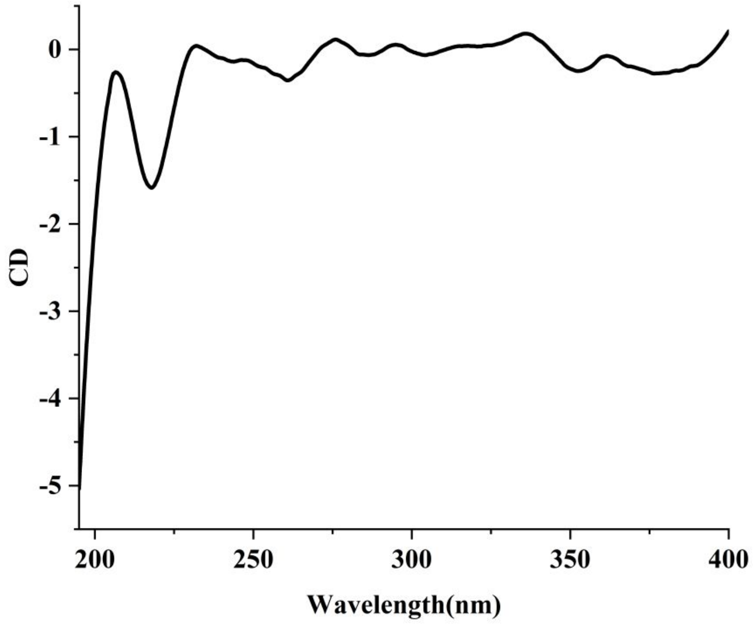

Figure S86. The CD spectrum of compound 9

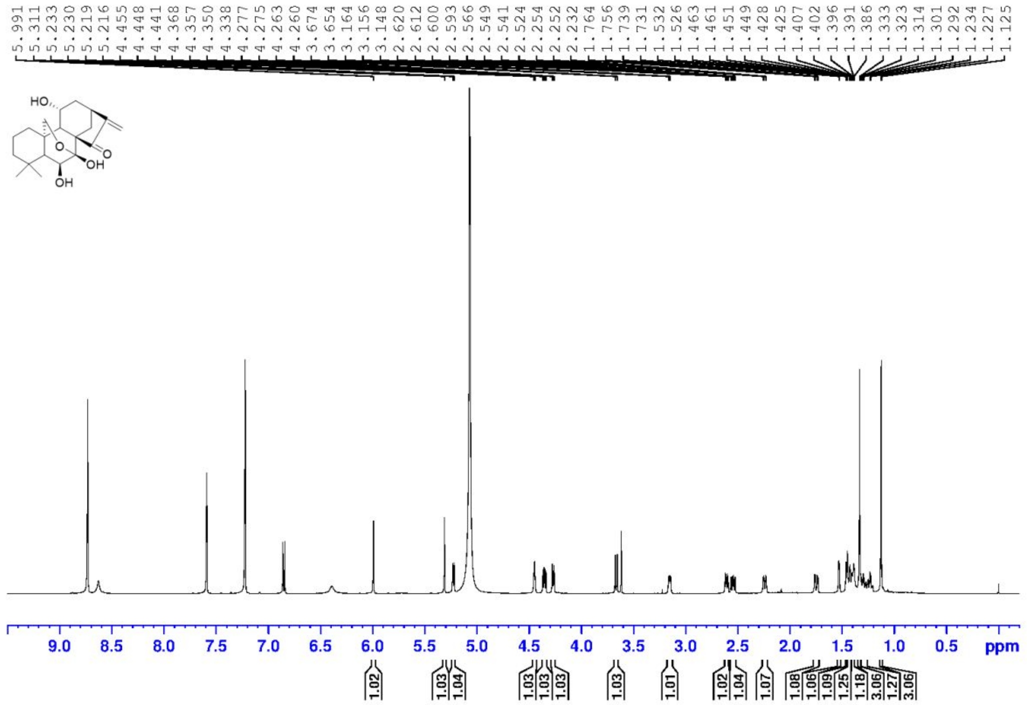

Figure S87. The ^{1}H NMR spectrum of compound 10

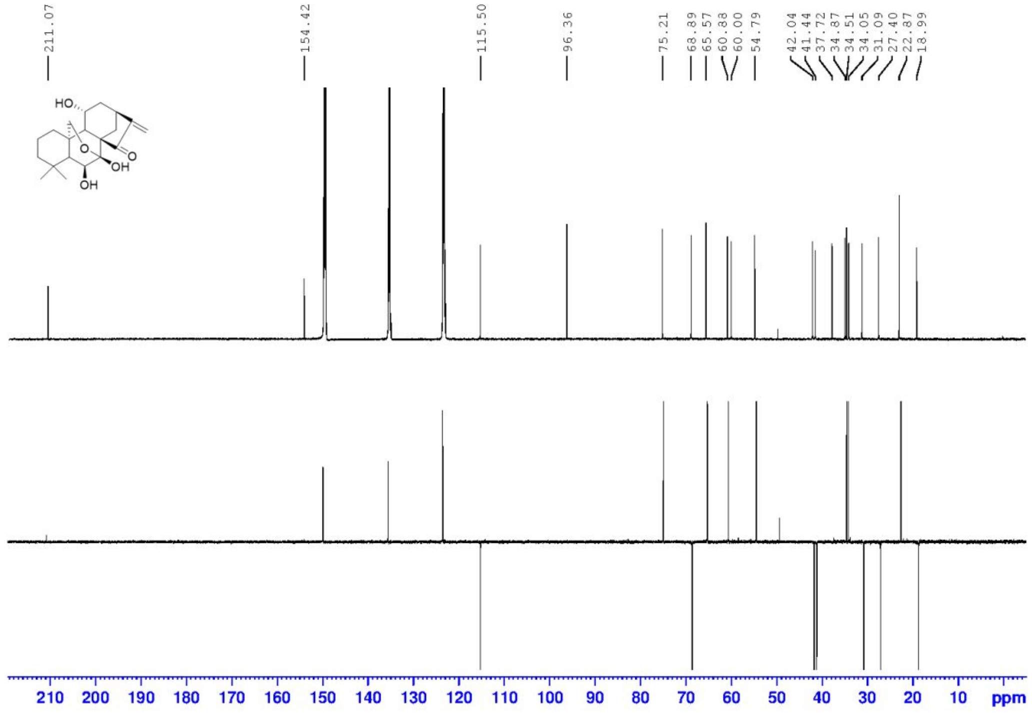

Figure S88. The ^{13}C and DEPT 135 NMR spectra of compound 10

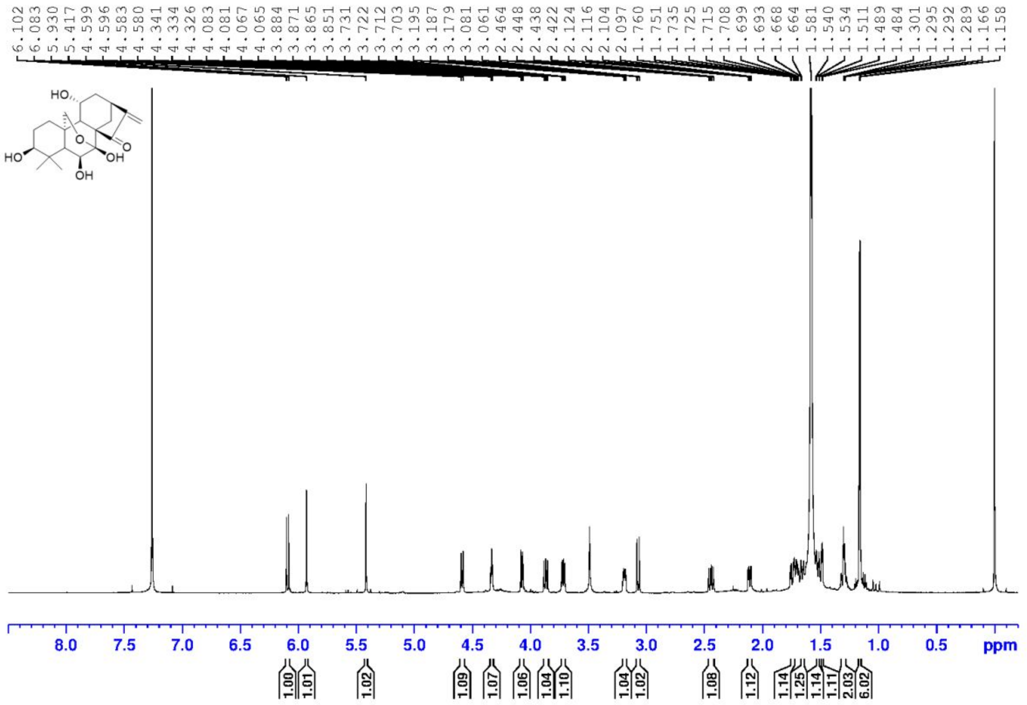

Figure S89. The ^{1}H NMR spectrum of compound 11

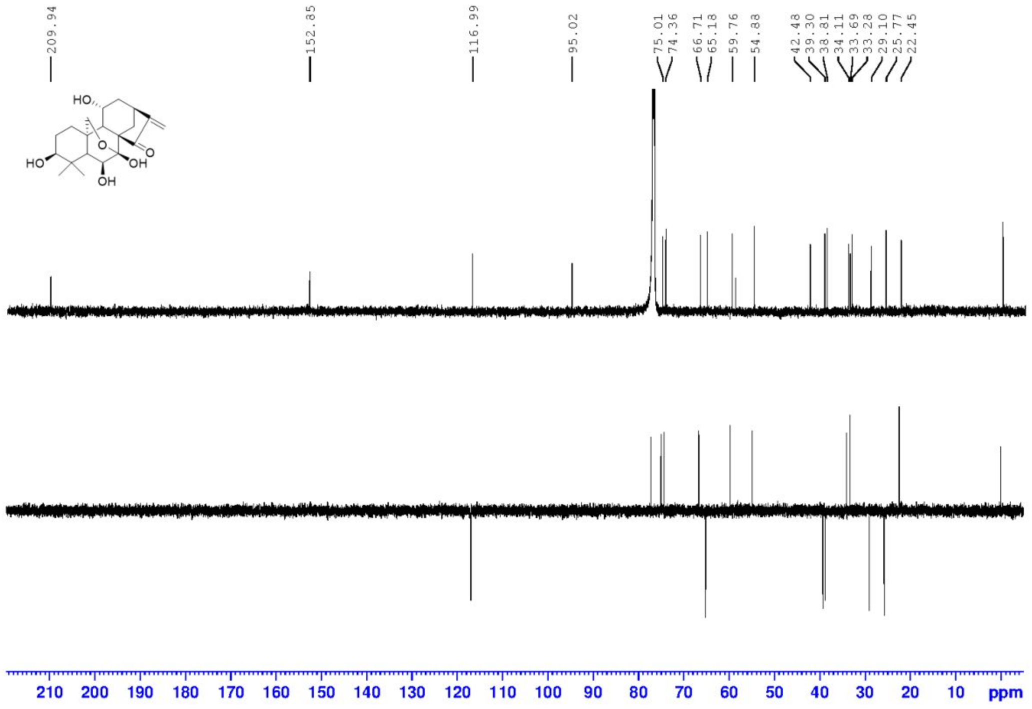

Figure S90. The ^{13}C and DEPT 135 NMR spectra of compound 11

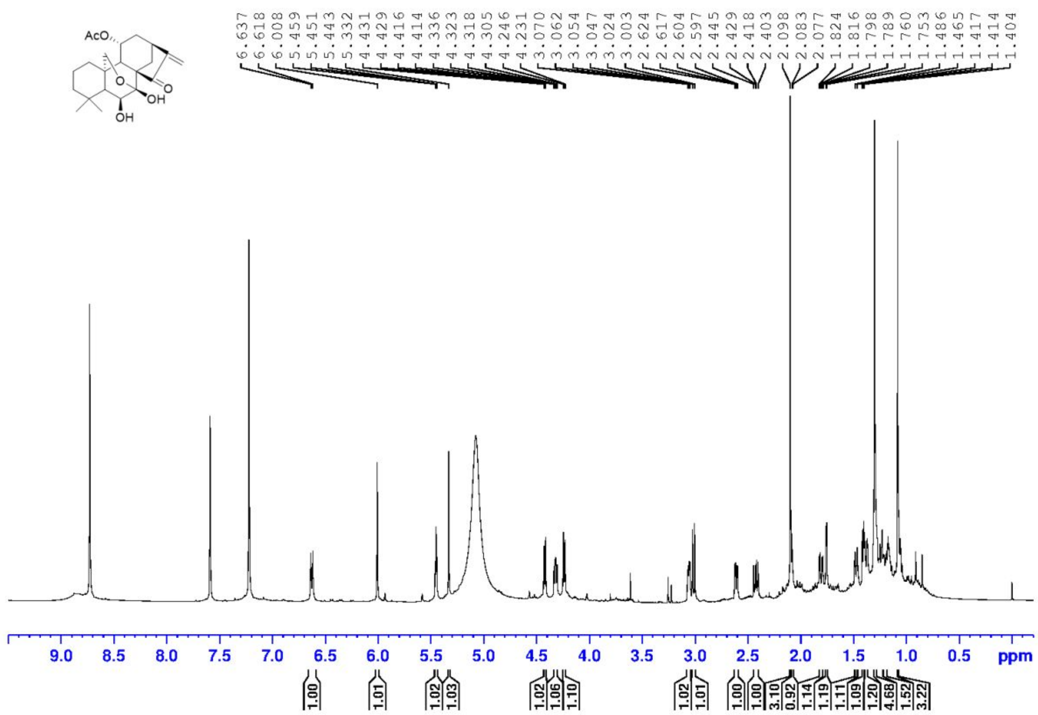

Figure S91. The ^{1}H NMR spectrum of compound 12

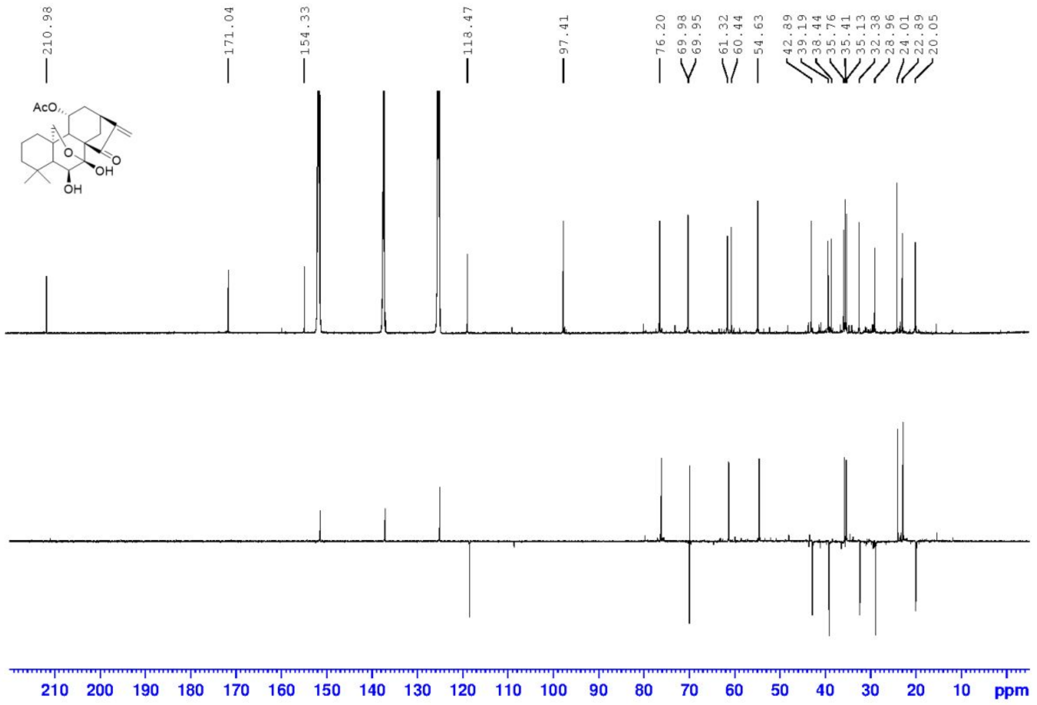

Figure S92. The ^{13}C and DEPT 135 NMR spectra of compound 12

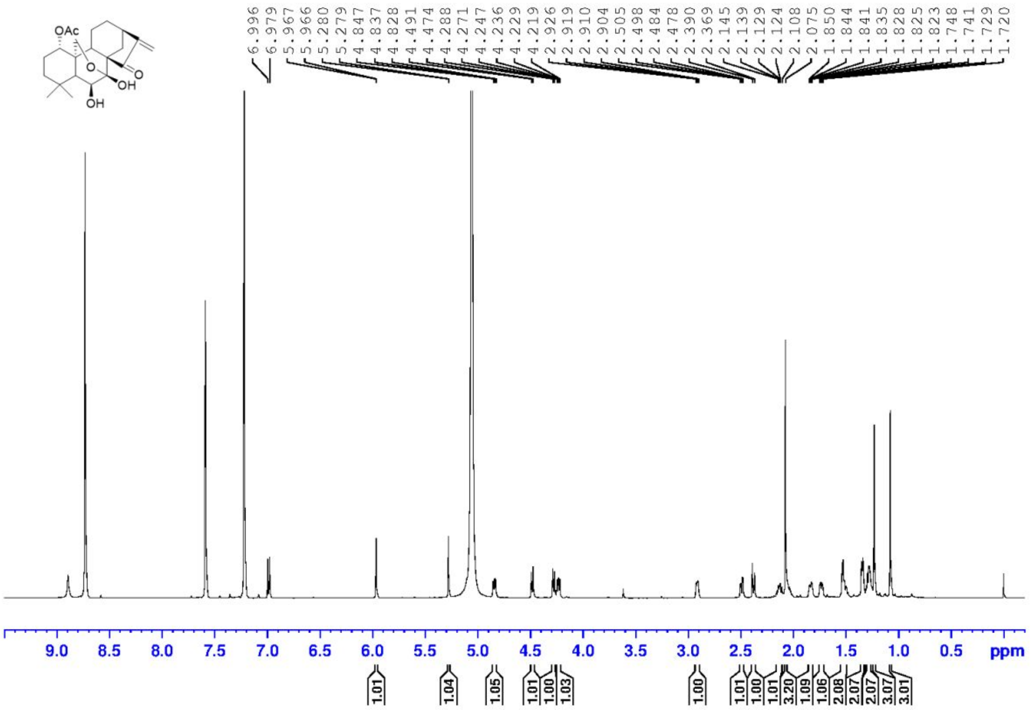

Figure S93. The ^{1}H NMR spectrum of compound 13

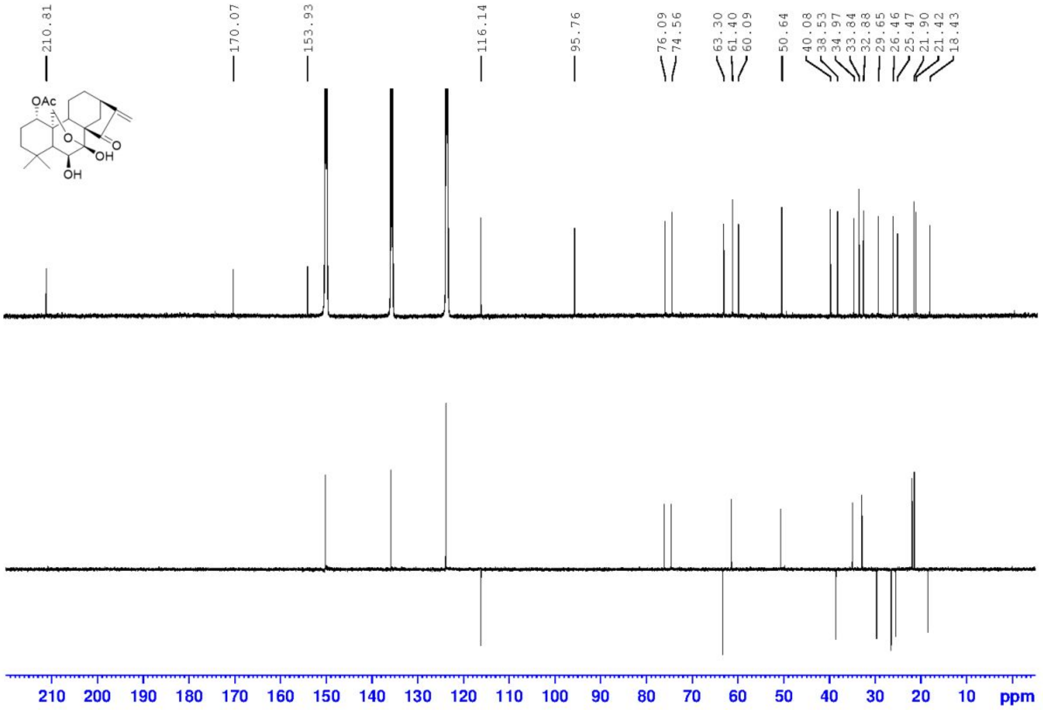

Figure S94. The ^{13}C and DEPT 135 NMR spectra of compound 13

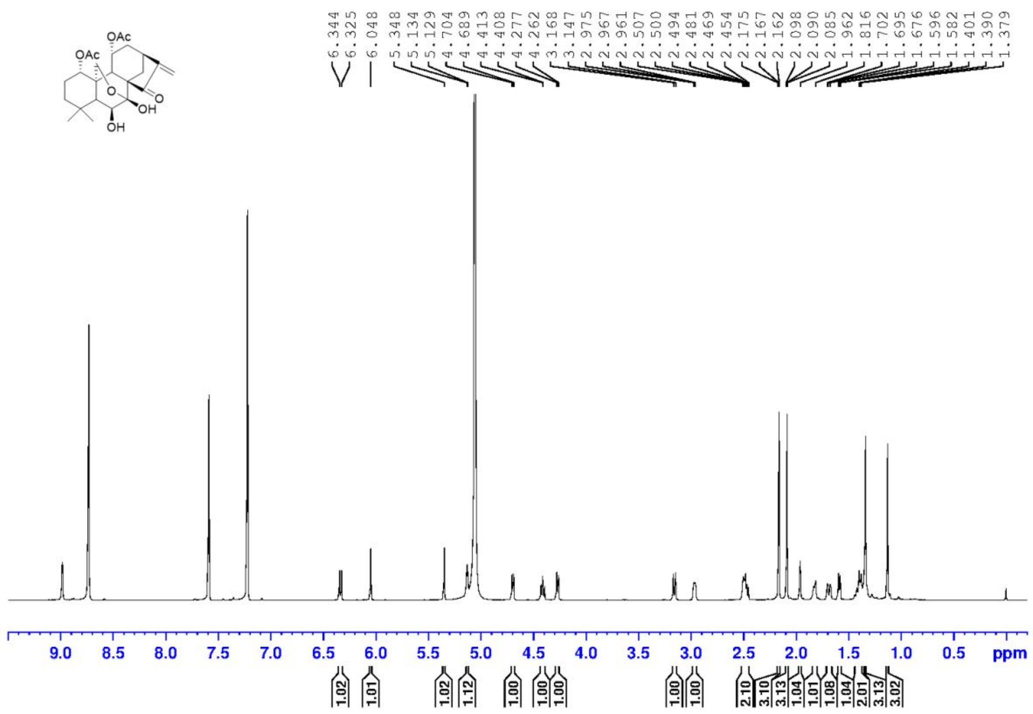

Figure S95. The ^{1}H NMR spectrum of compound 14

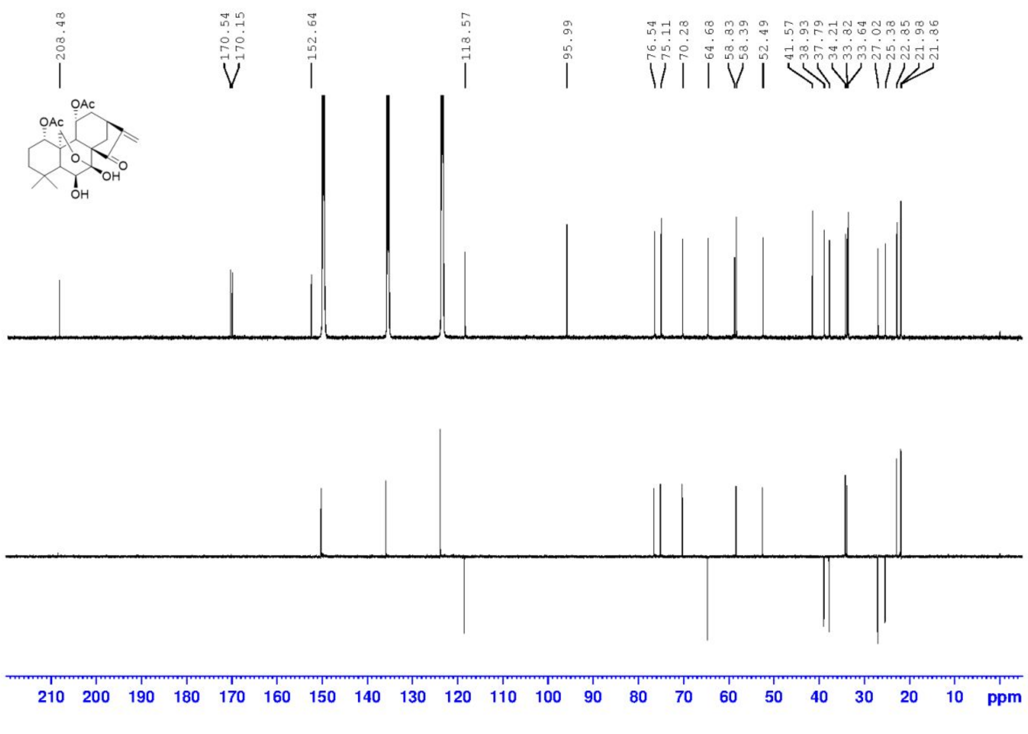

Figure S96. The ^{13}C and DEPT 135 NMR spectra of compound 14

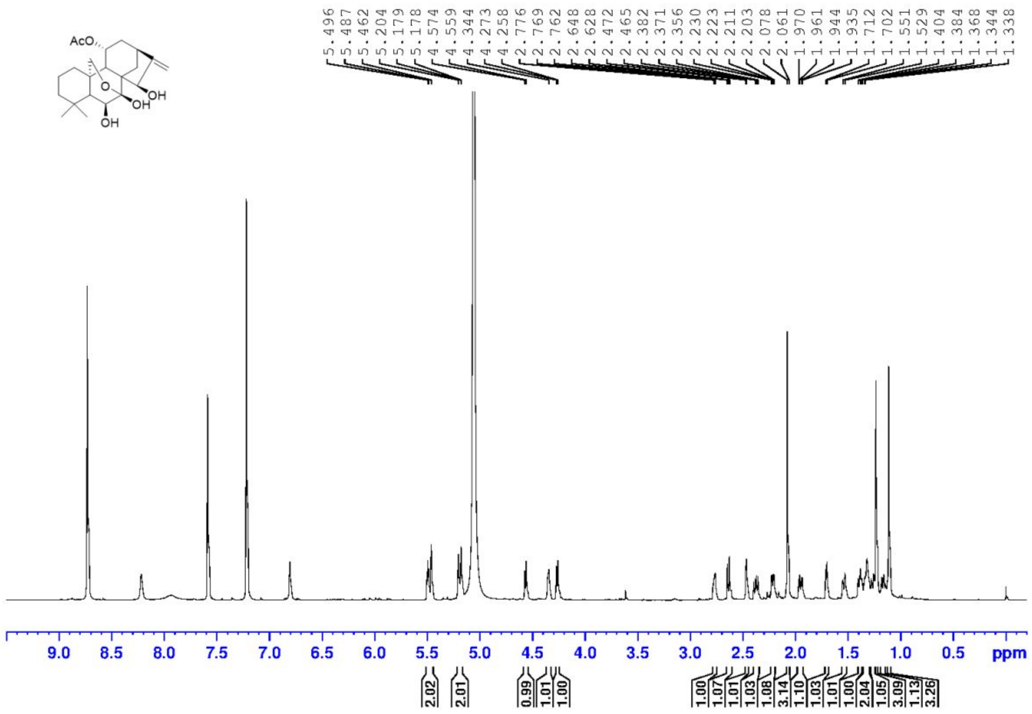

Figure S97. The ^{1}H NMR spectrum of compound 15

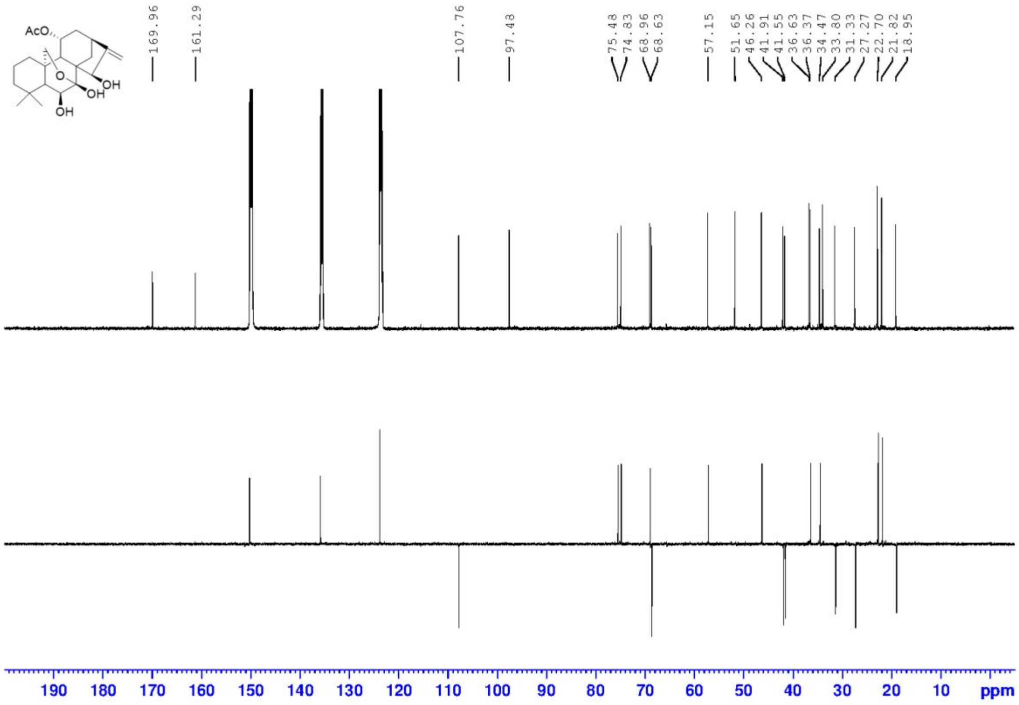

Figure S98. The ^{13}C and DEPT 135 NMR spectra of compound 15

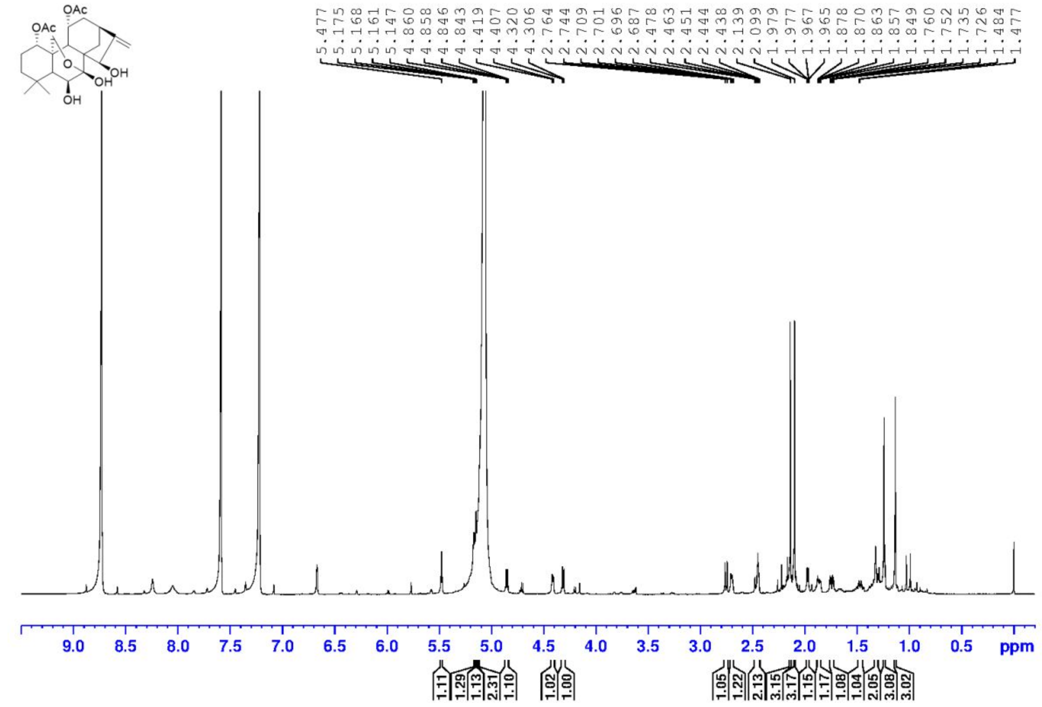

Figure S99. The ^{1}H NMR spectrum of compound 16

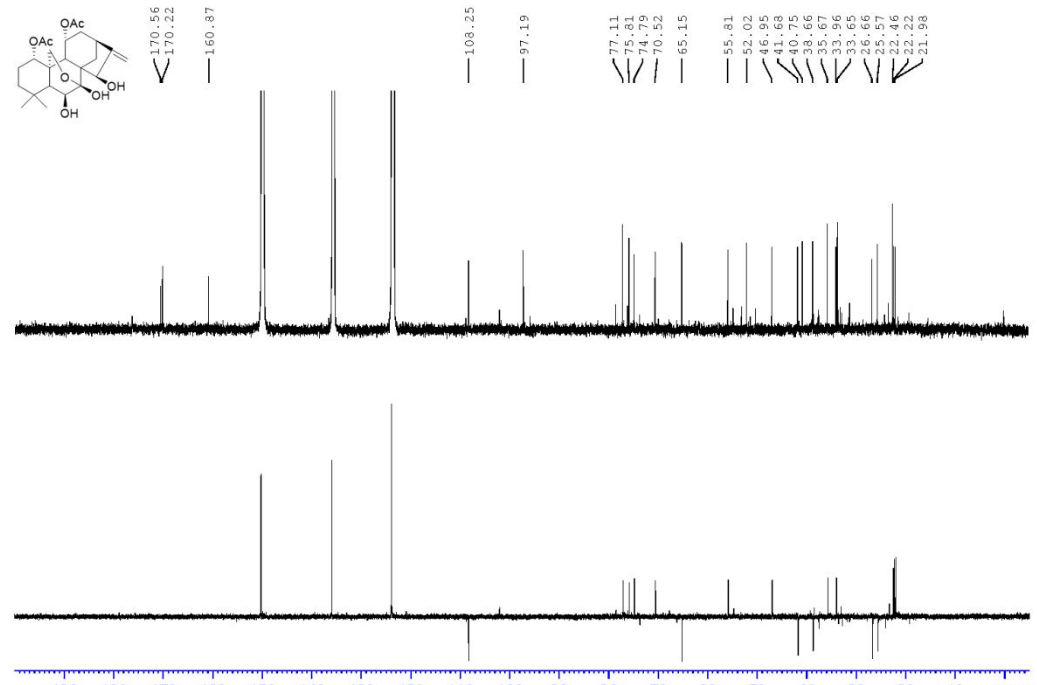

Figure S100. The ^{13}C and DEPT 135 NMR spectra of compound 16

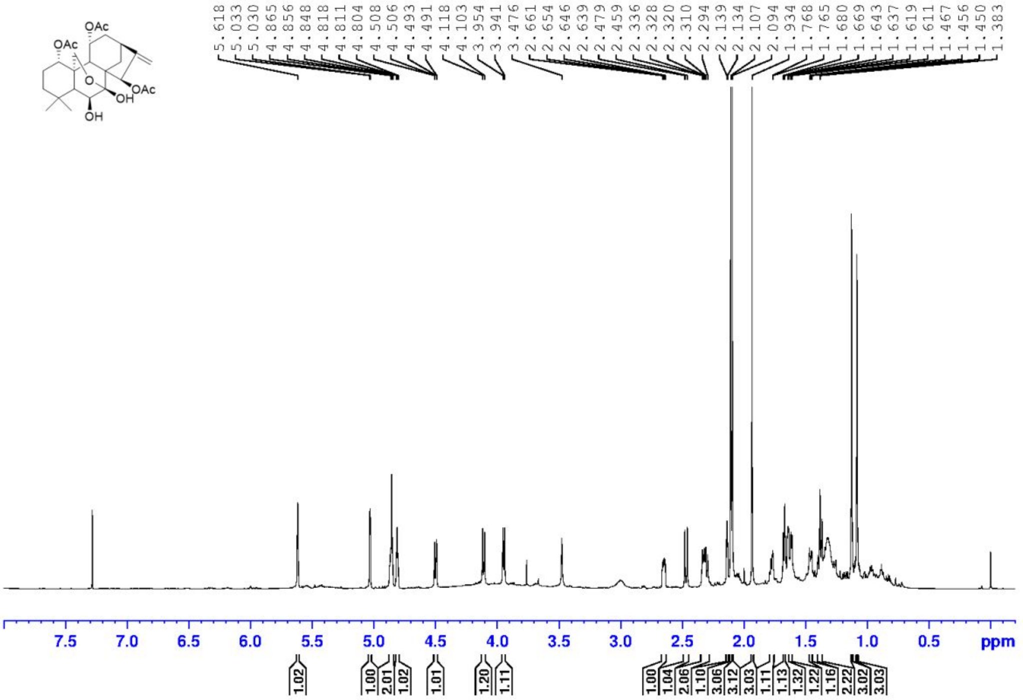

Figure S101. The ^{1}H NMR spectrum of compound 17

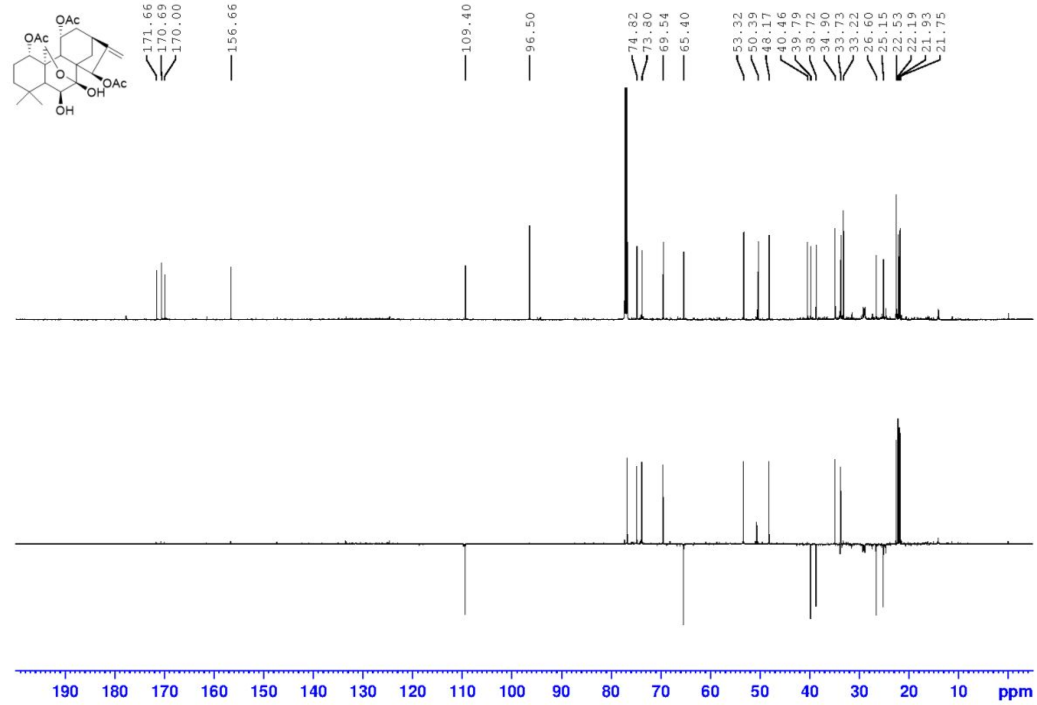

Figure S102. The ^{13}C and DEPT 135 NMR spectra of compound 17

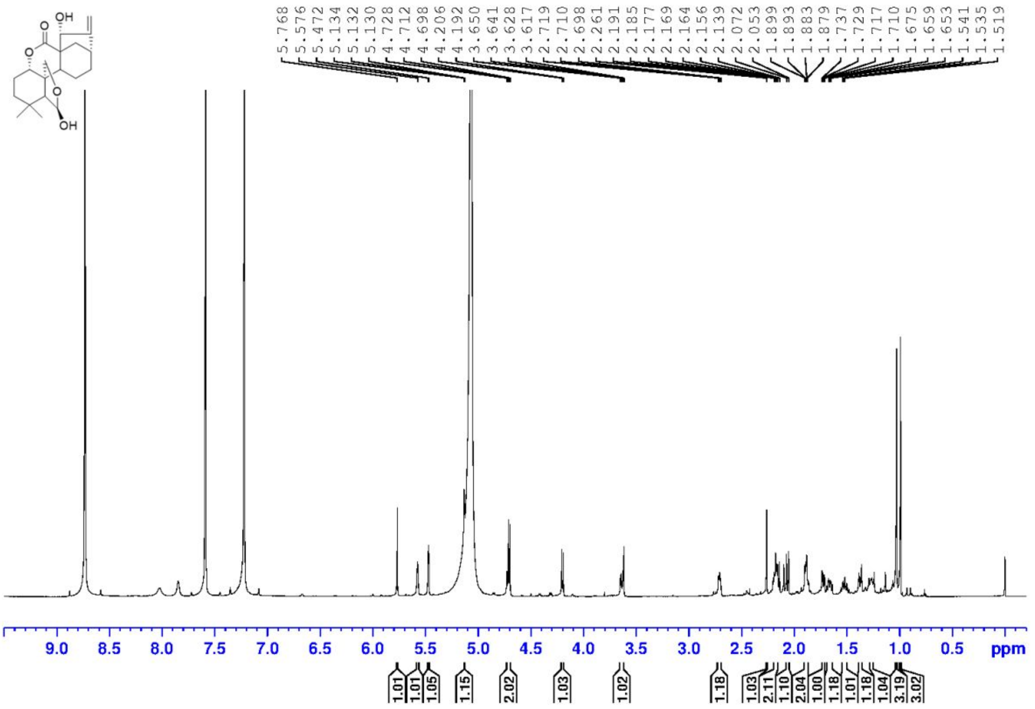

Figure S103. The ^{1}H NMR spectrum of compound 18

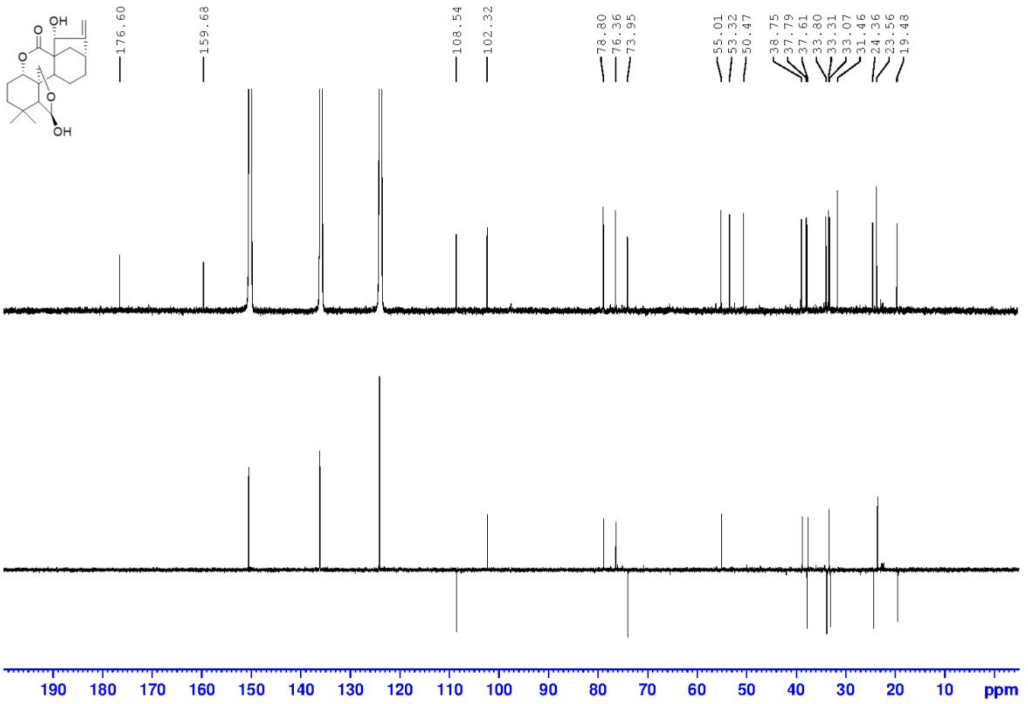

Figure S104. The ^{13}C and DEPT 135 NMR spectra of compound 18

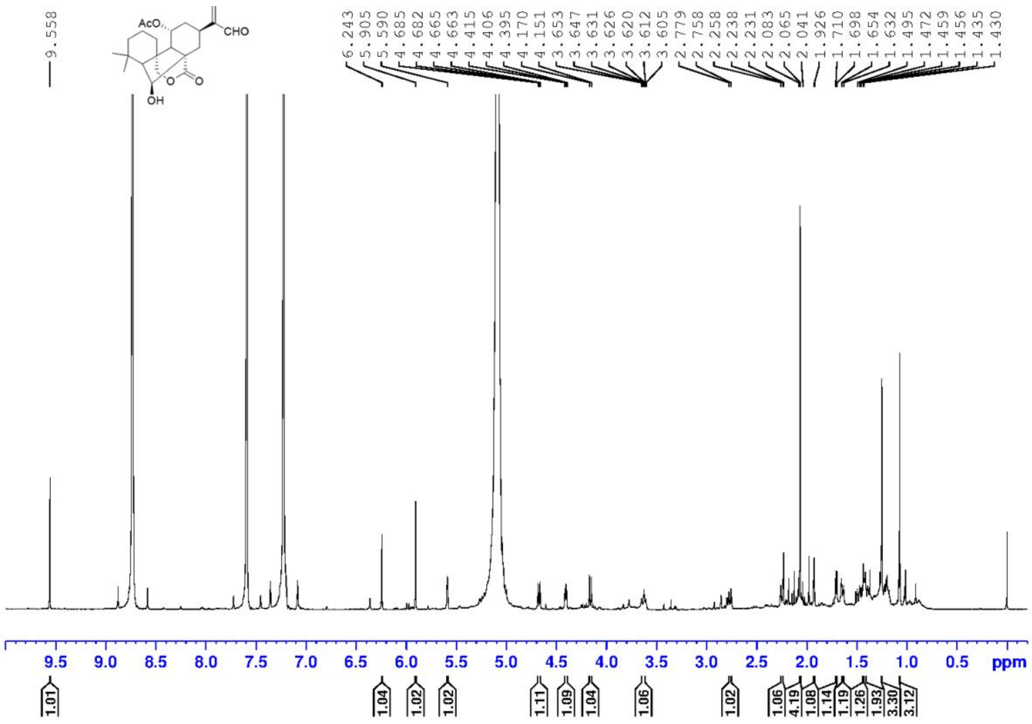

Figure S105. The ^{1}H NMR spectrum of compound 19

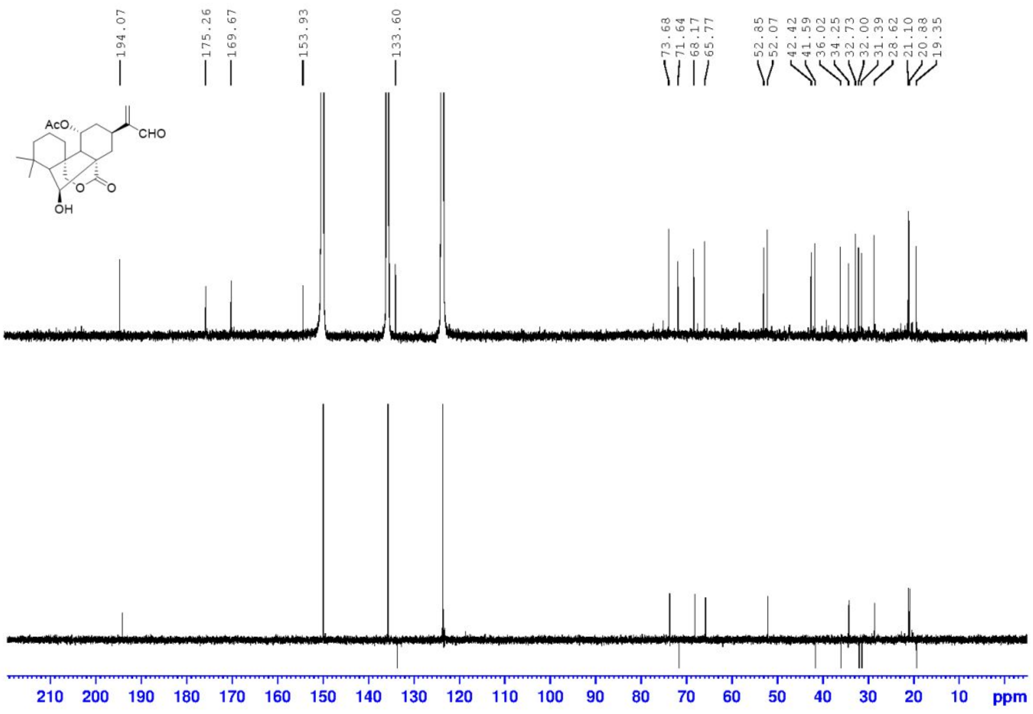

Figure S106. The ^{13}C and DEPT 135 NMR spectra of compound 19

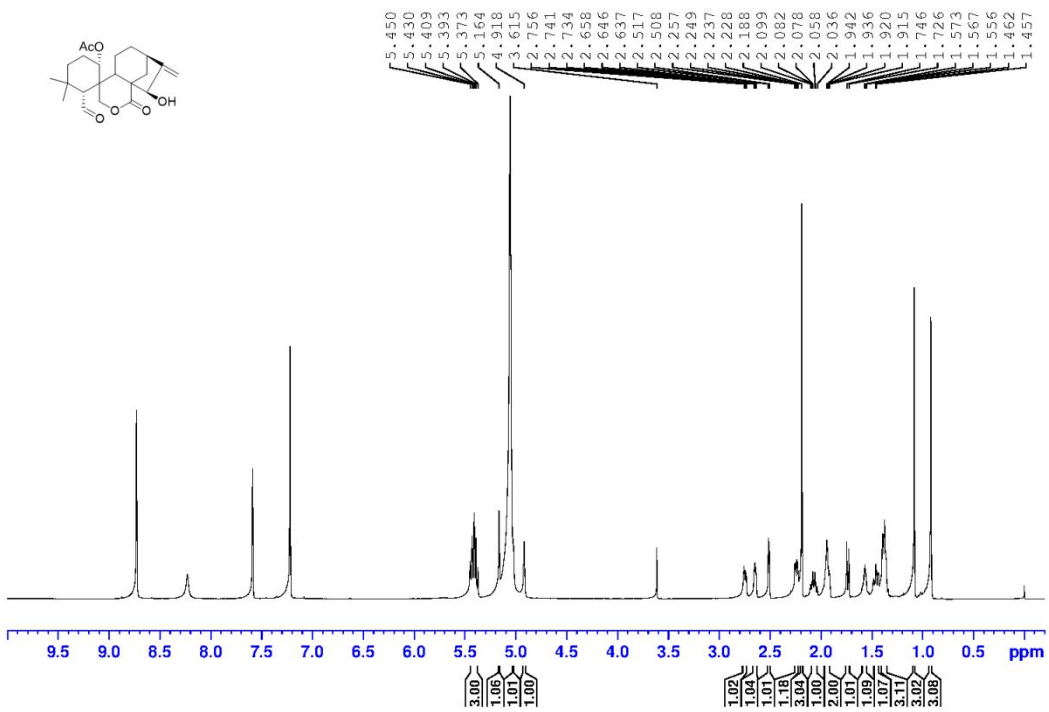

Figure S107. The ^{1}H NMR spectrum of compound 20

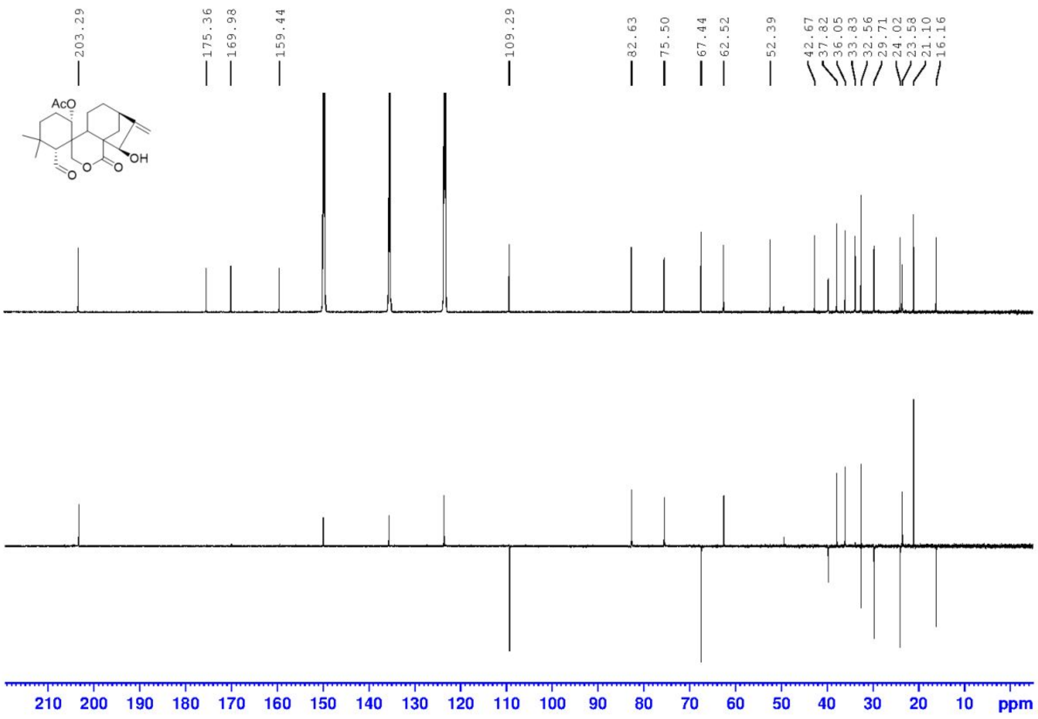

Figure S108. The ^{13}C and DEPT 135 NMR spectra of compound 20

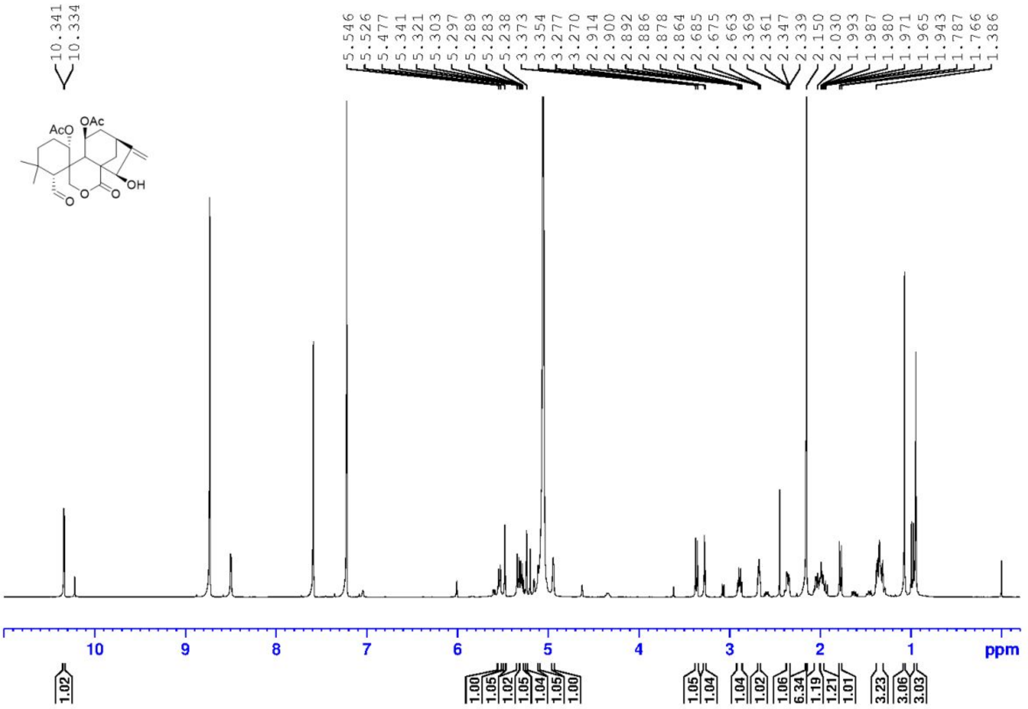

Figure S109. The ^{1}H NMR spectrum of compound 21

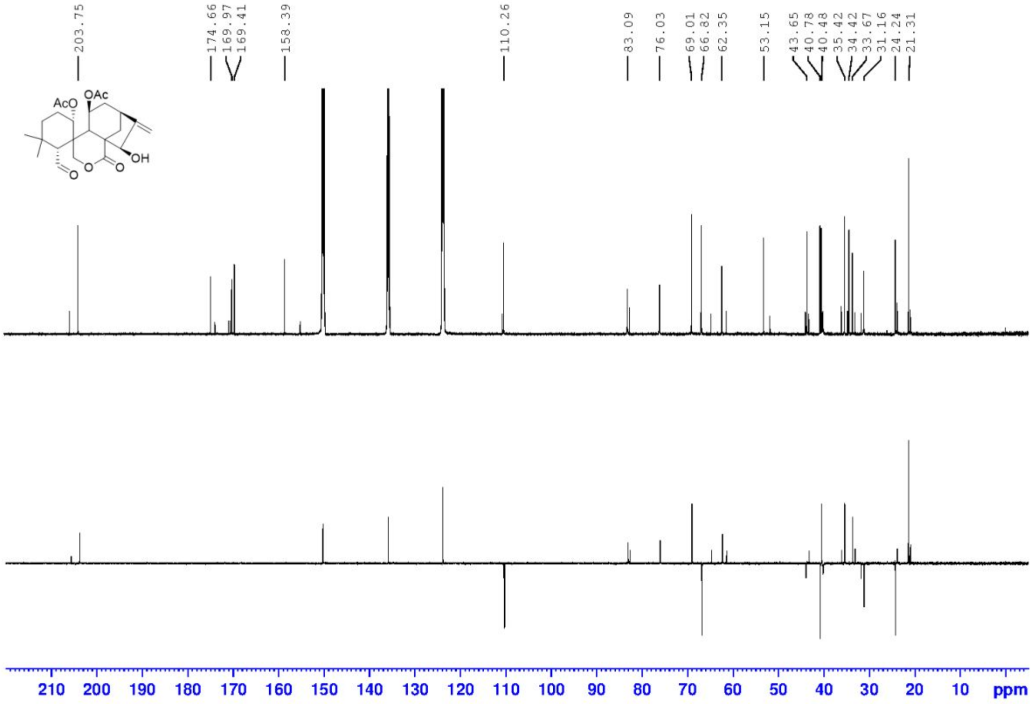

Figure S110. The ^{13}C and DEPT 135 NMR spectra of compound 21

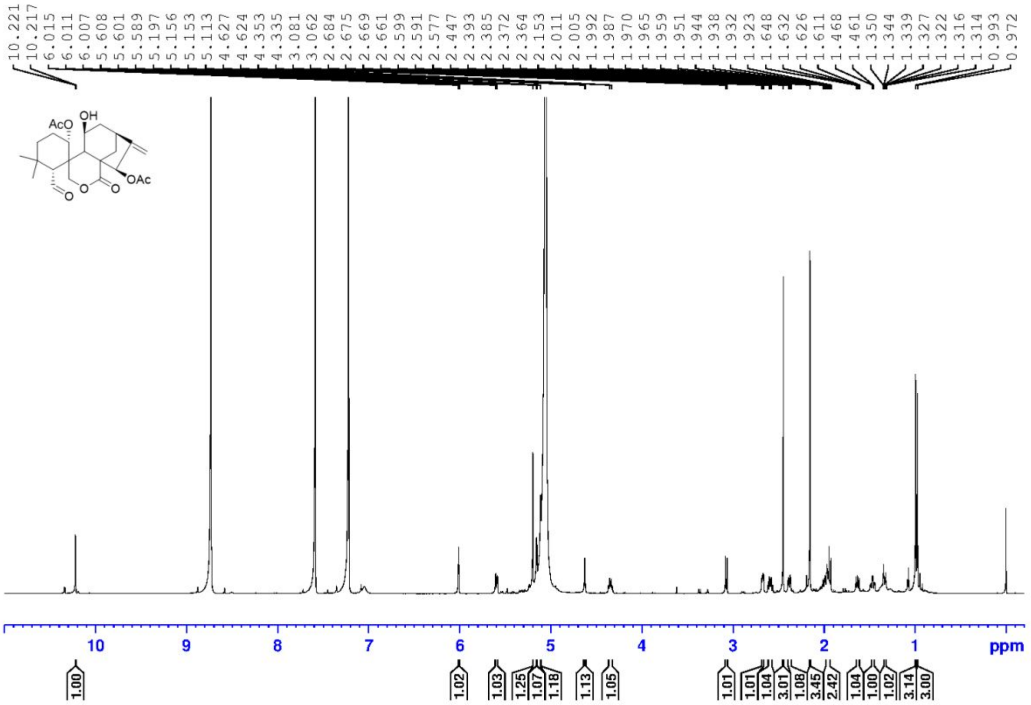

Figure S111. The ^{1}H NMR spectrum of compound 22

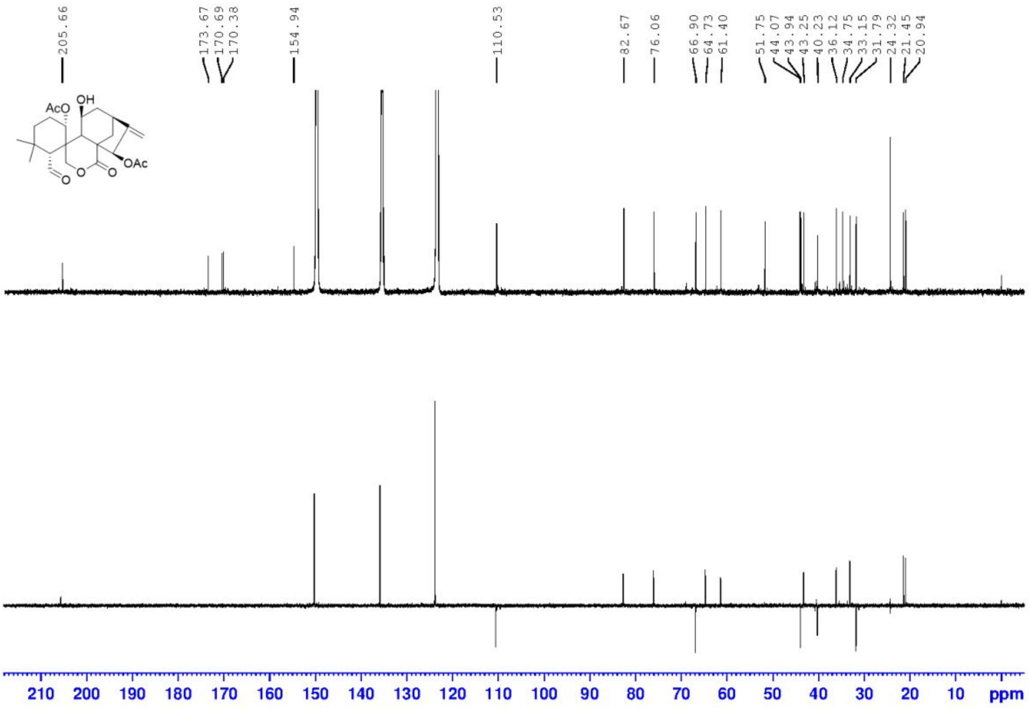

Figure S112. The ^{13}C and DEPT 135 NMR spectra of compound 22

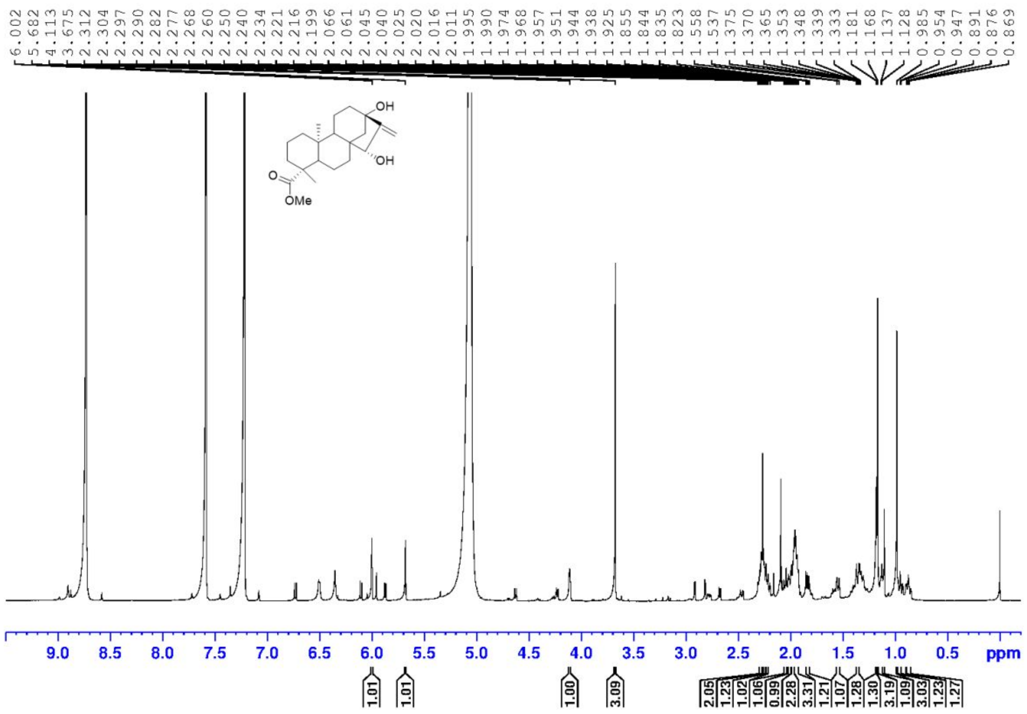

Figure S113. The ^{1}H NMR spectrum of compound 23

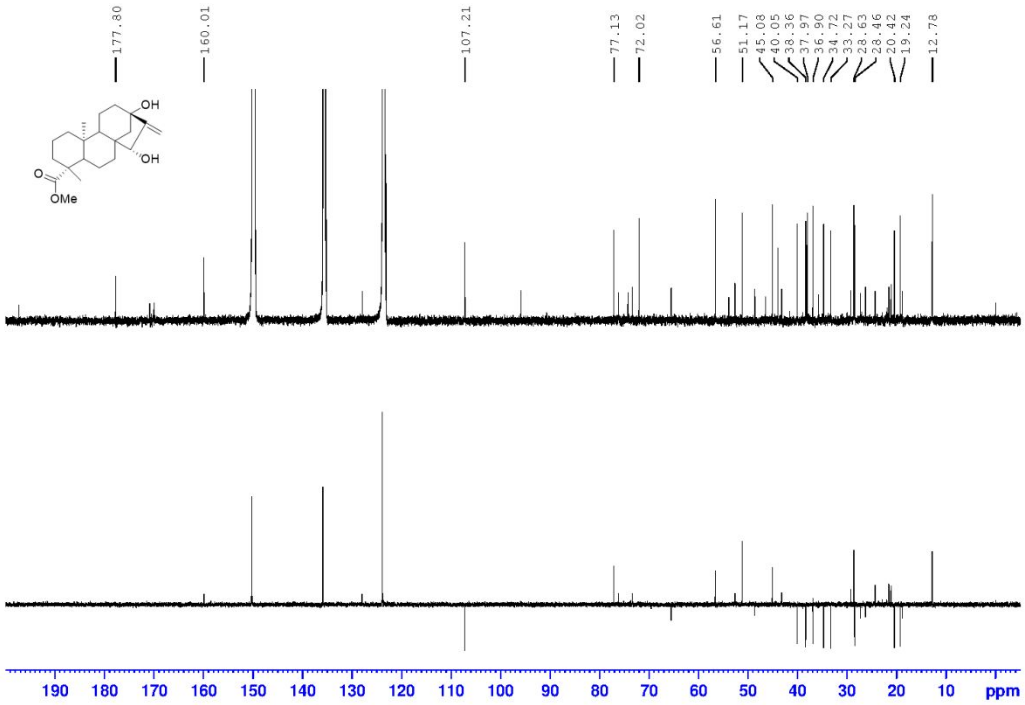

Figure S114. The ^{13}C and DEPT 135 NMR spectra of compound 23

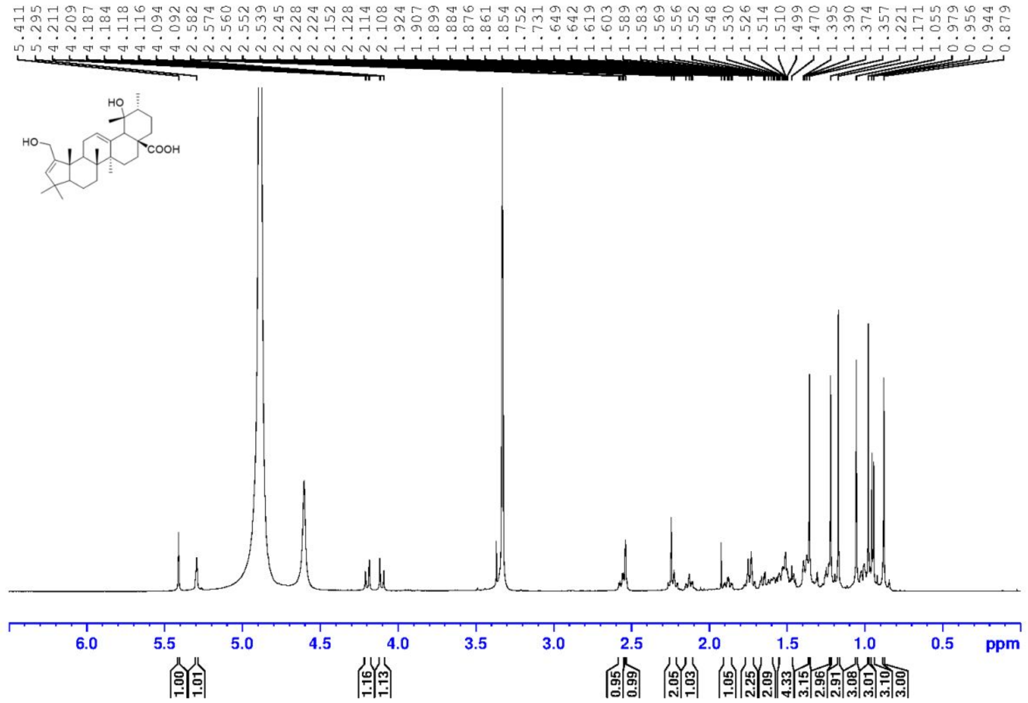

Figure S115. The ^{1}H NMR spectrum of compound 24

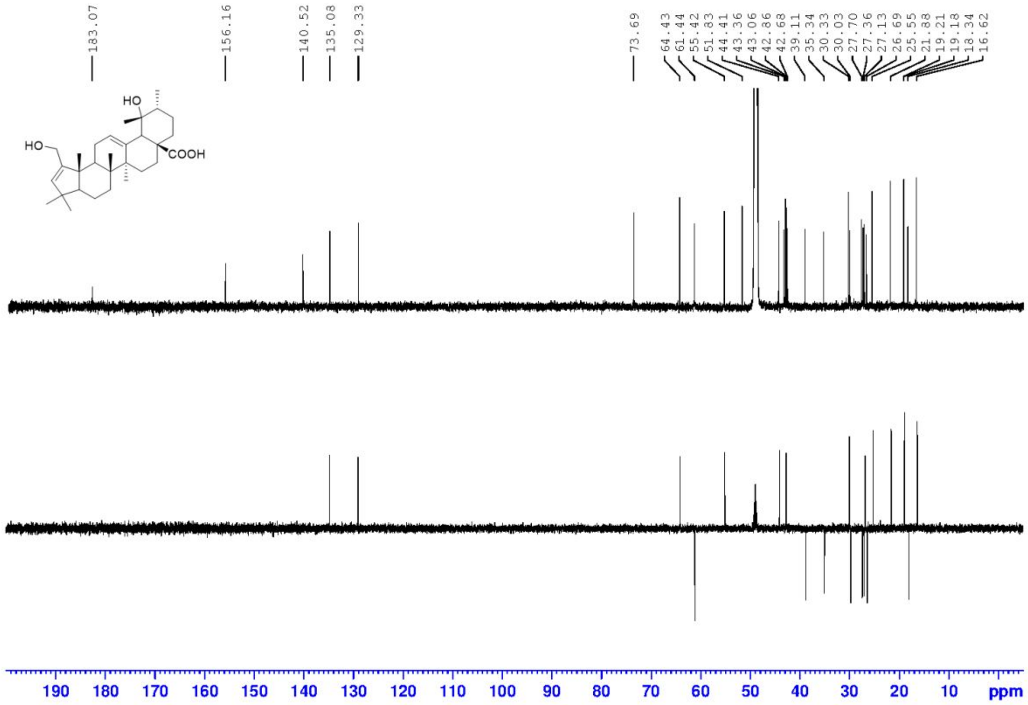

Figure S116. The ^{13}C and DEPT 135 NMR spectra of compound 24

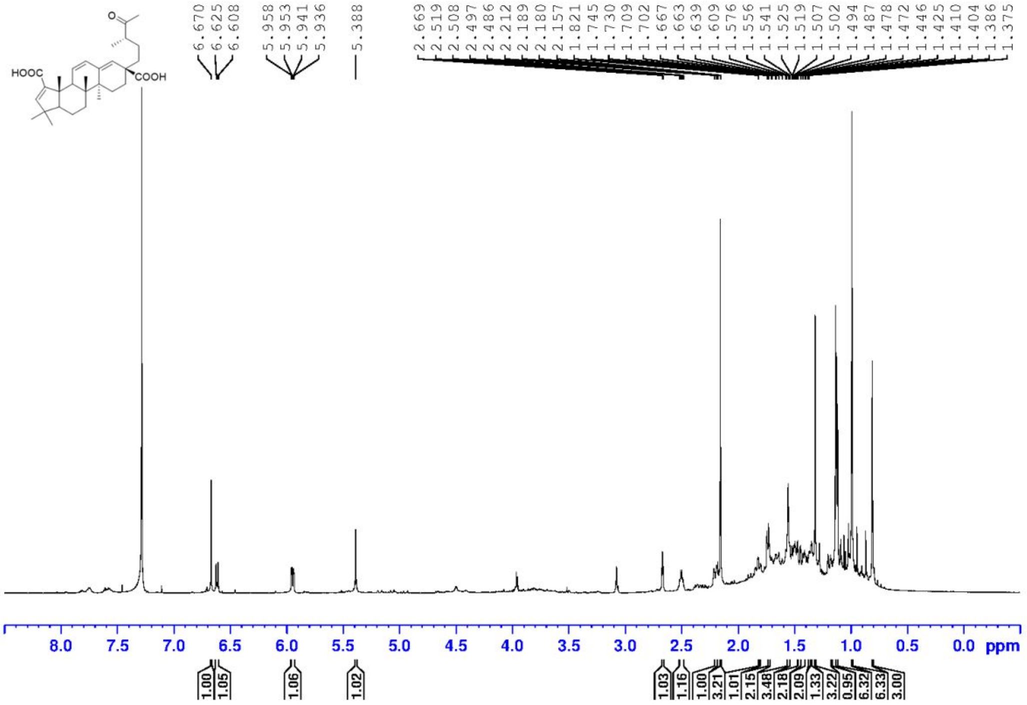

Figure S117. The ^{1}H NMR spectrum of compound 25

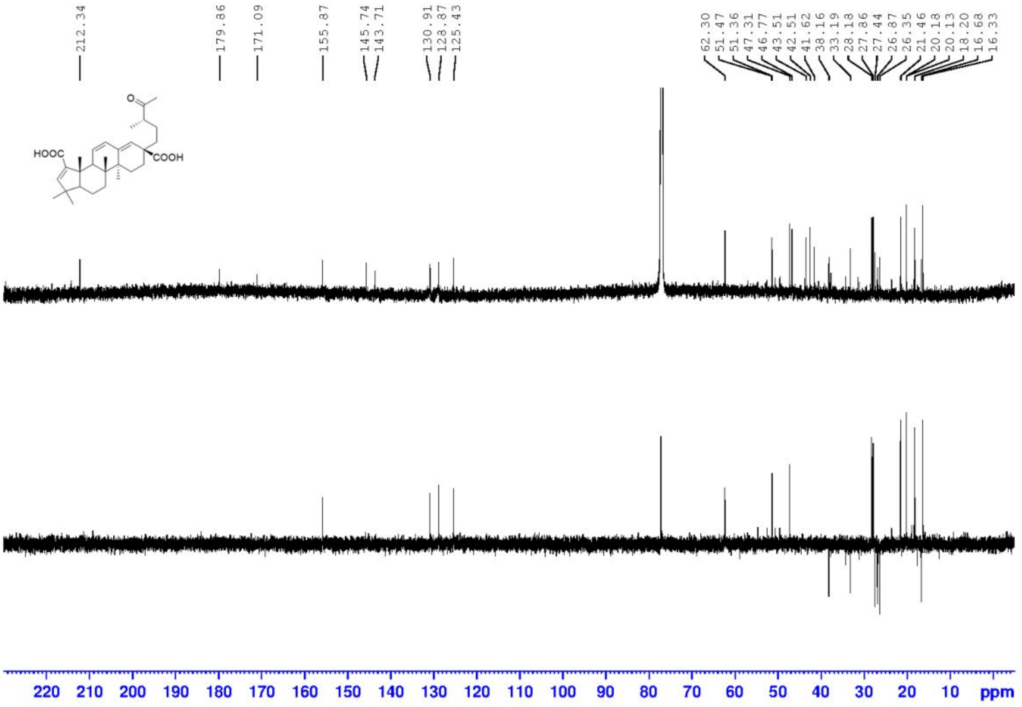

Figure S118. The ^{13}C and DEPT 135 NMR spectra of compound 25

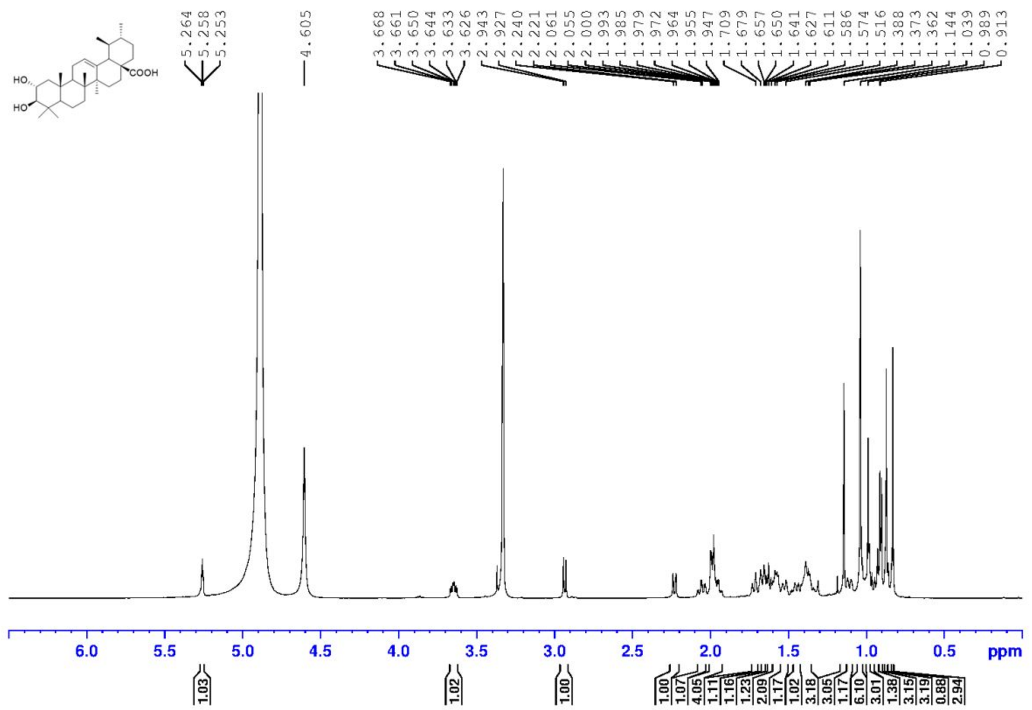

Figure S119. The ^{1}H NMR spectrum of compound 26

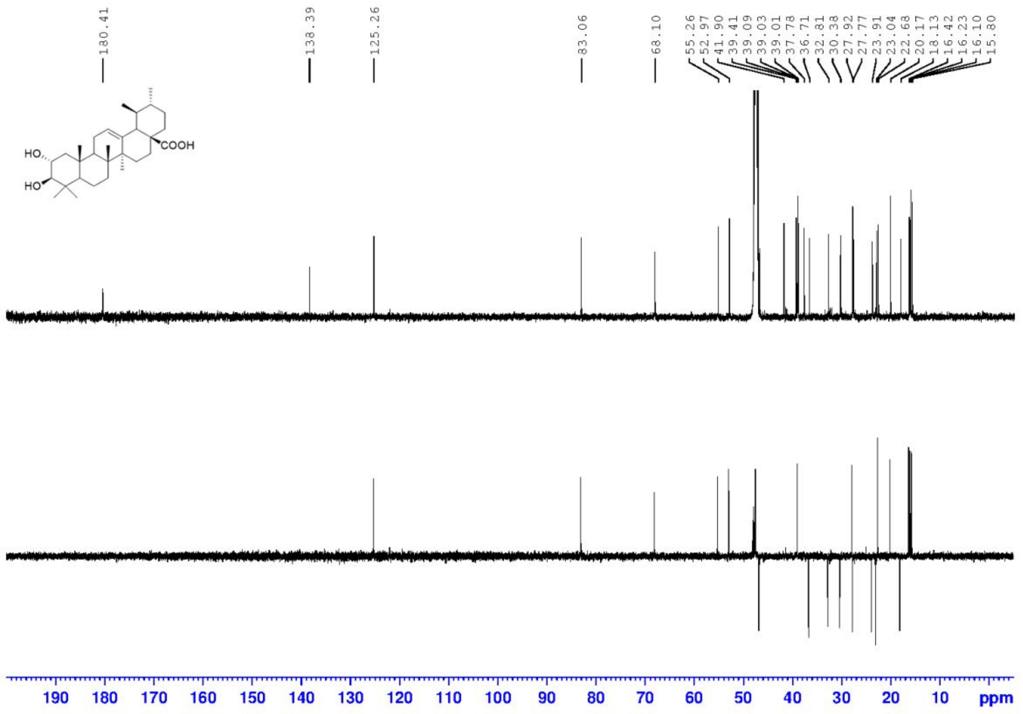

Figure S120. The ^{13}C and DEPT 135 NMR spectra of compound 26

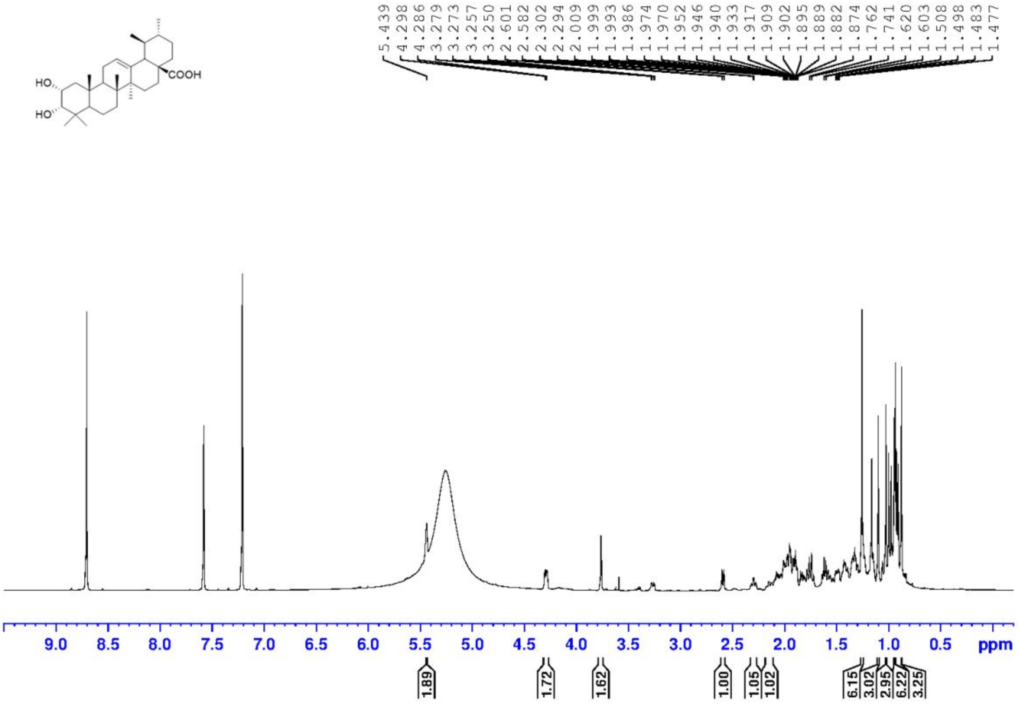

Figure S121. The ^{1}H NMR spectrum of compound 27

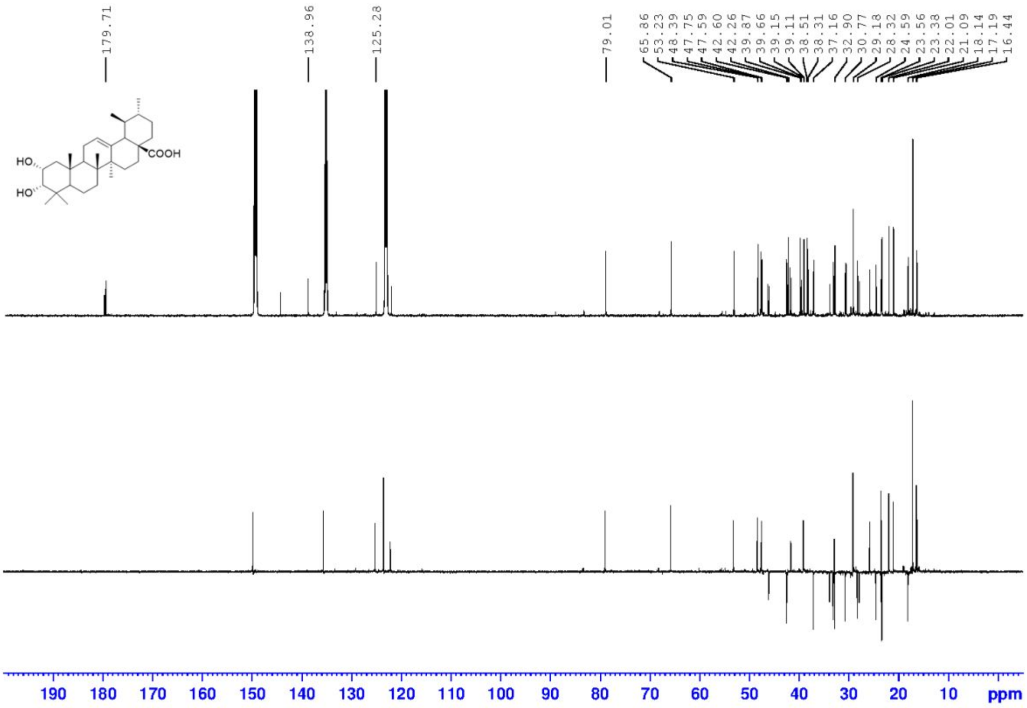

Figure S122. The ^{13}C and DEPT 135 NMR spectra of compound 27

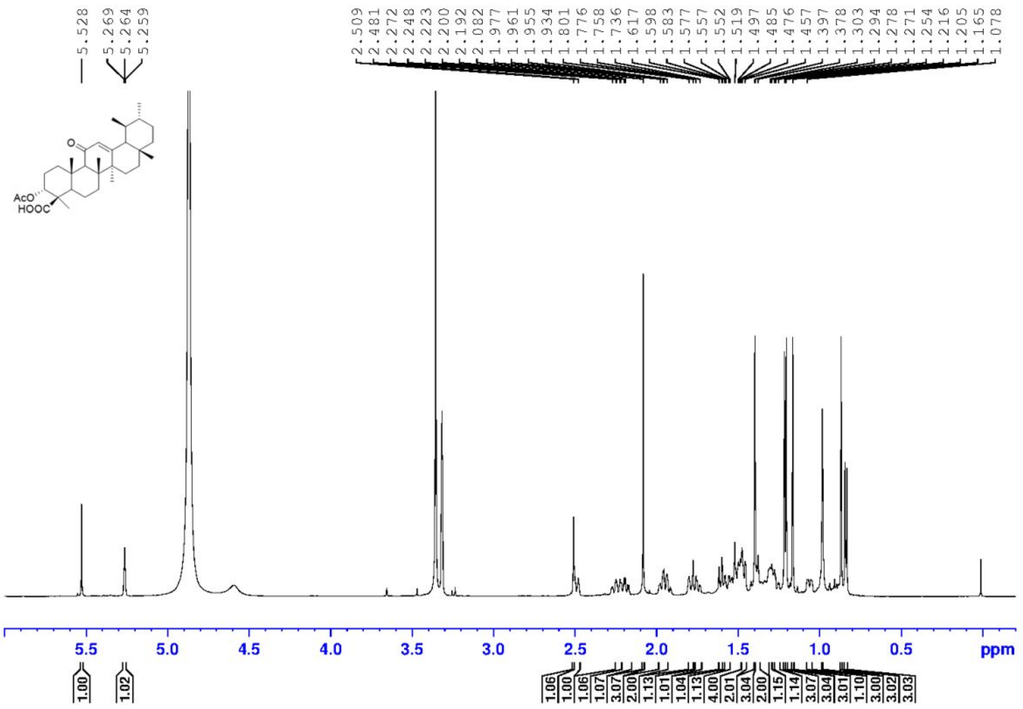

Figure S123. The ^{1}H NMR spectrum of compound 28

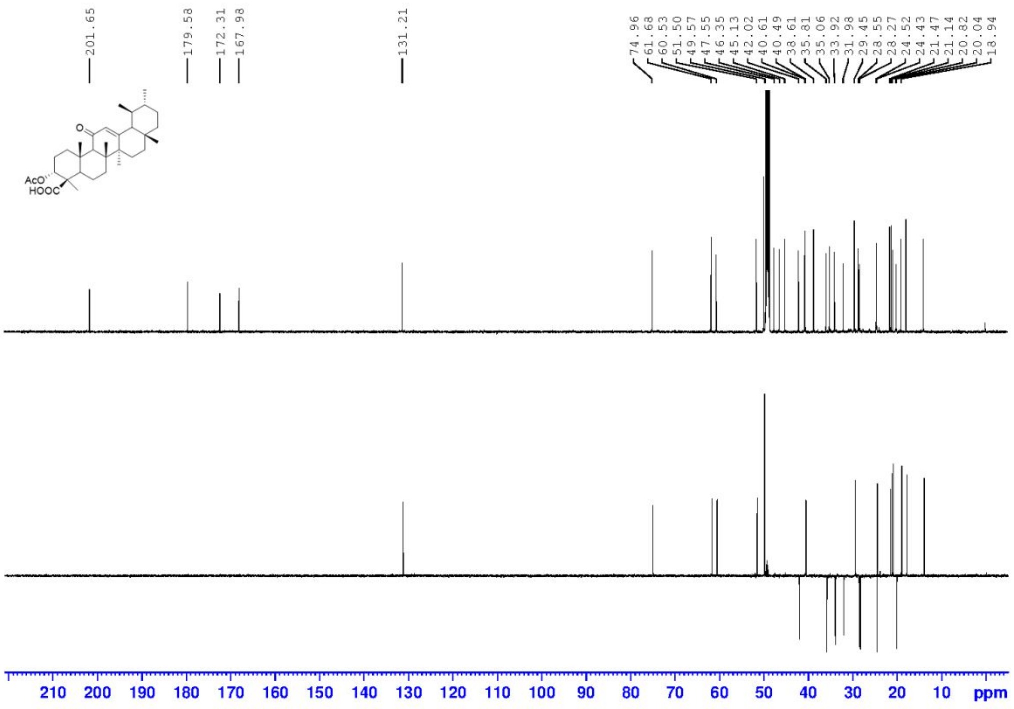

Figure S124. The ^{13}C and DEPT 135 NMR spectra of compound 28

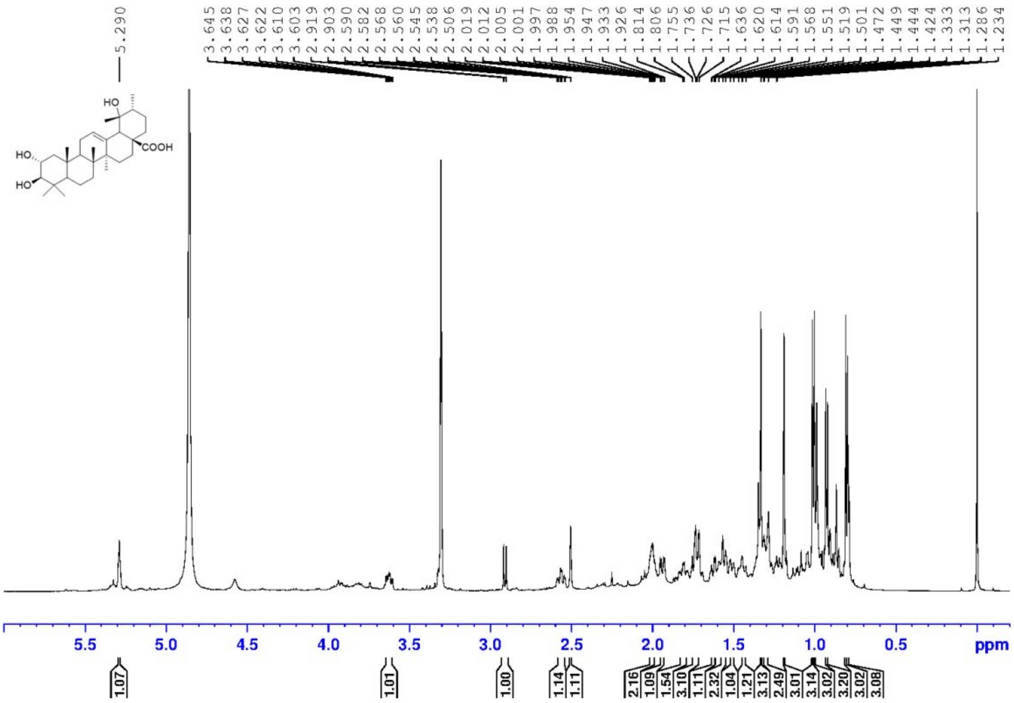

Figure S125. The ^{1}H NMR spectrum of compound 29

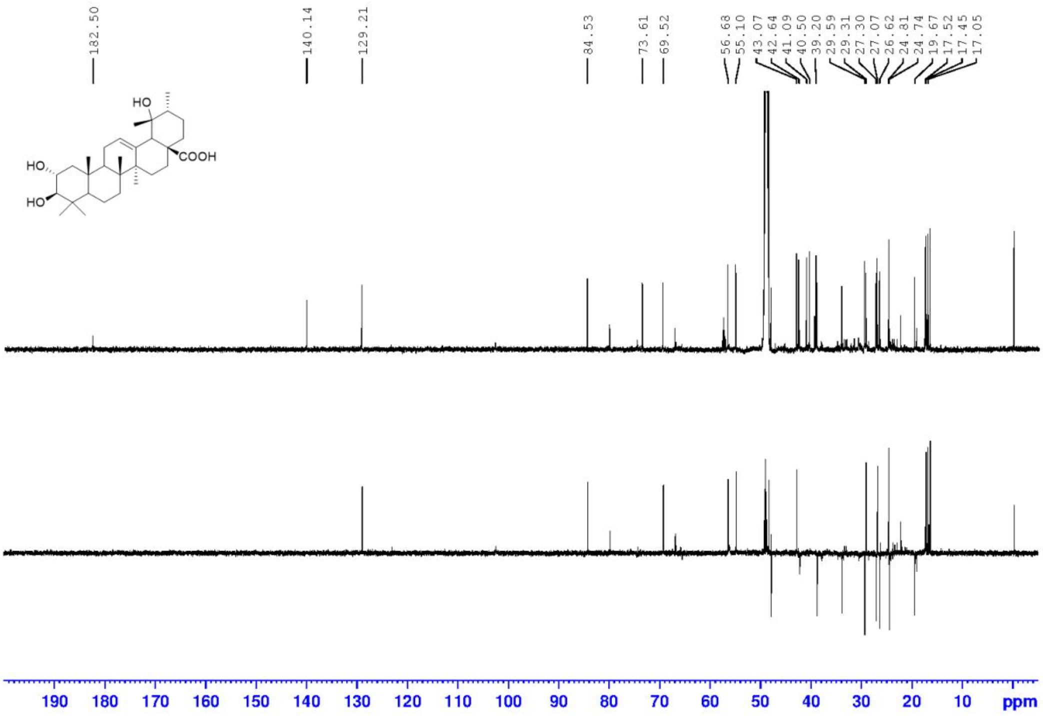

Figure S126. The ^{13}C and DEPT 135 NMR spectra of compound 29

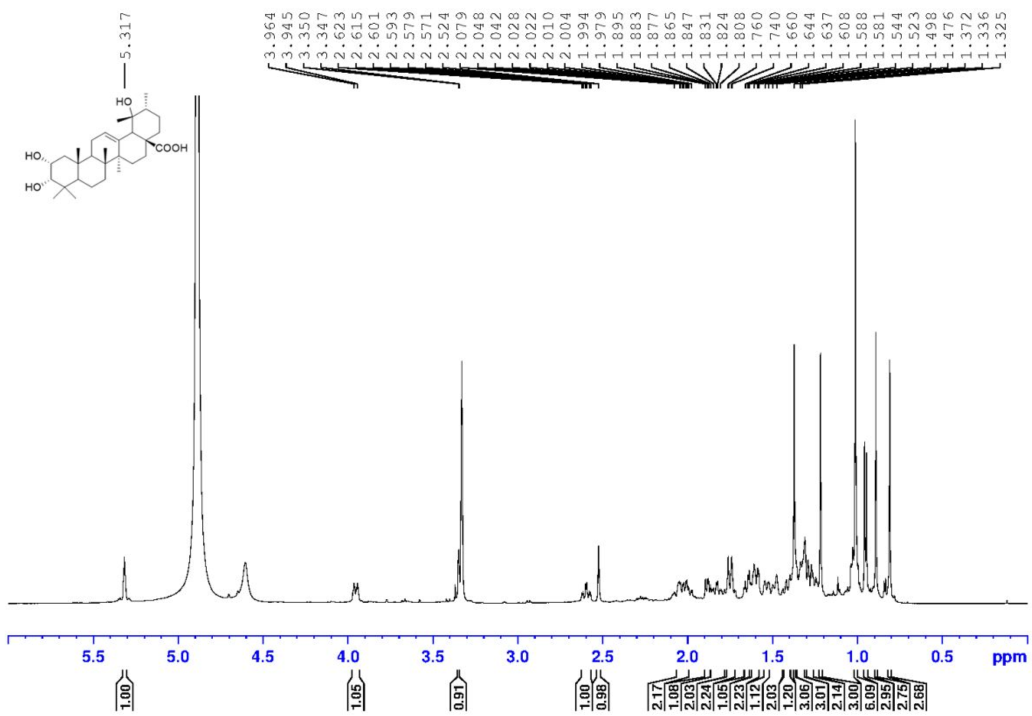

Figure S127. The ^{1}H NMR spectrum of compound 30

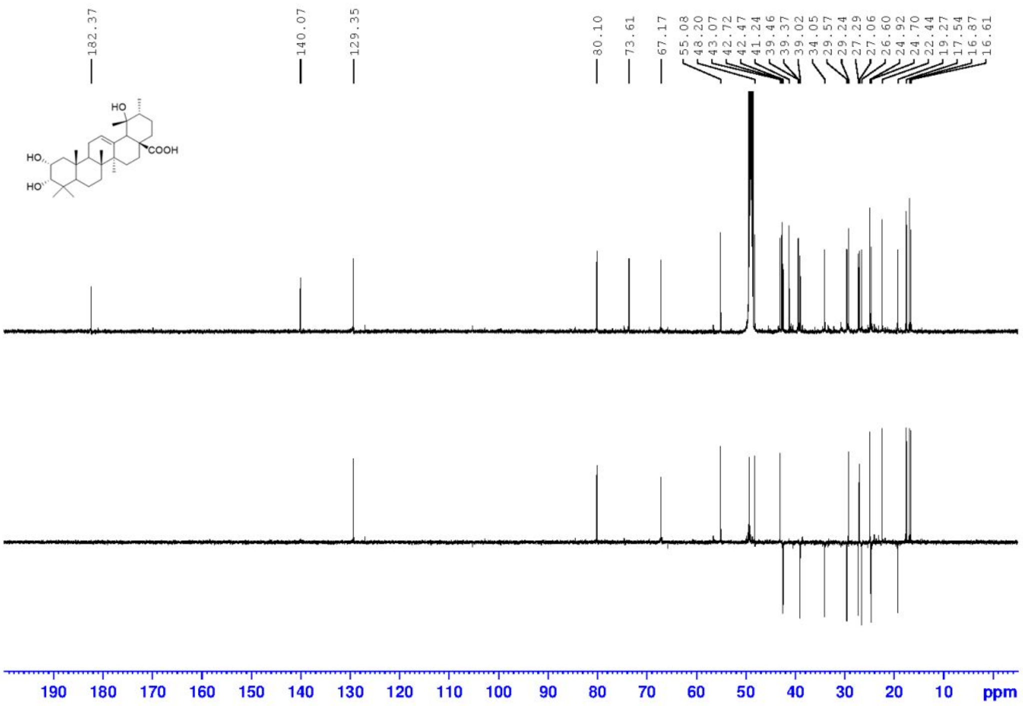

Figure S128. The ^{13}C and DEPT 135 NMR spectra of compound 30

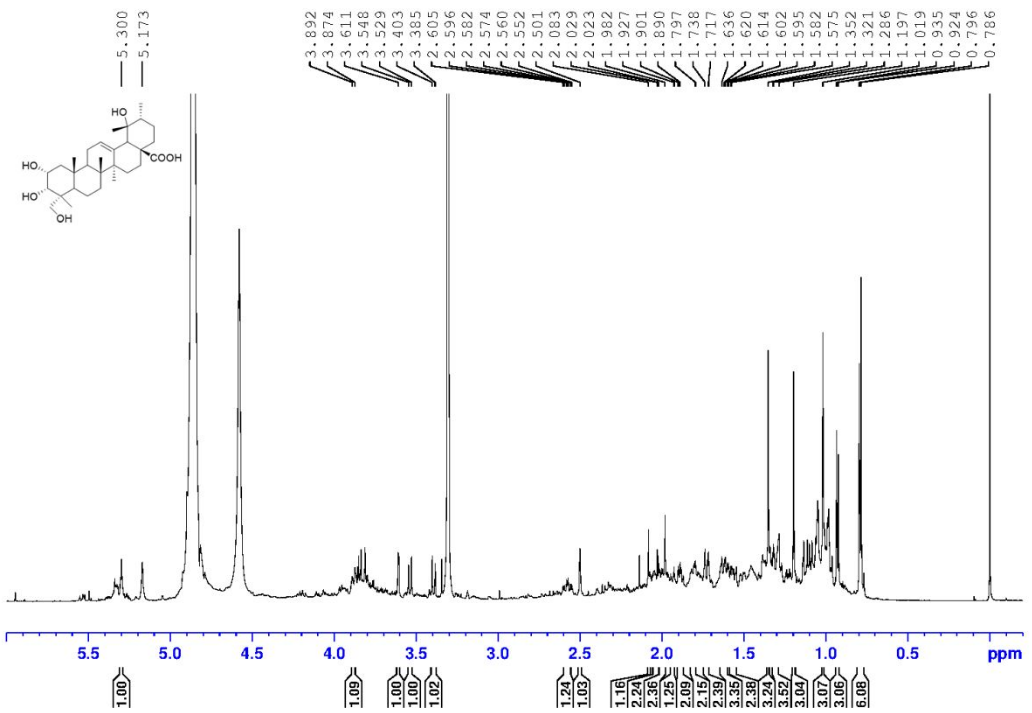

Figure S129. The ^{1}H NMR spectrum of compound 31

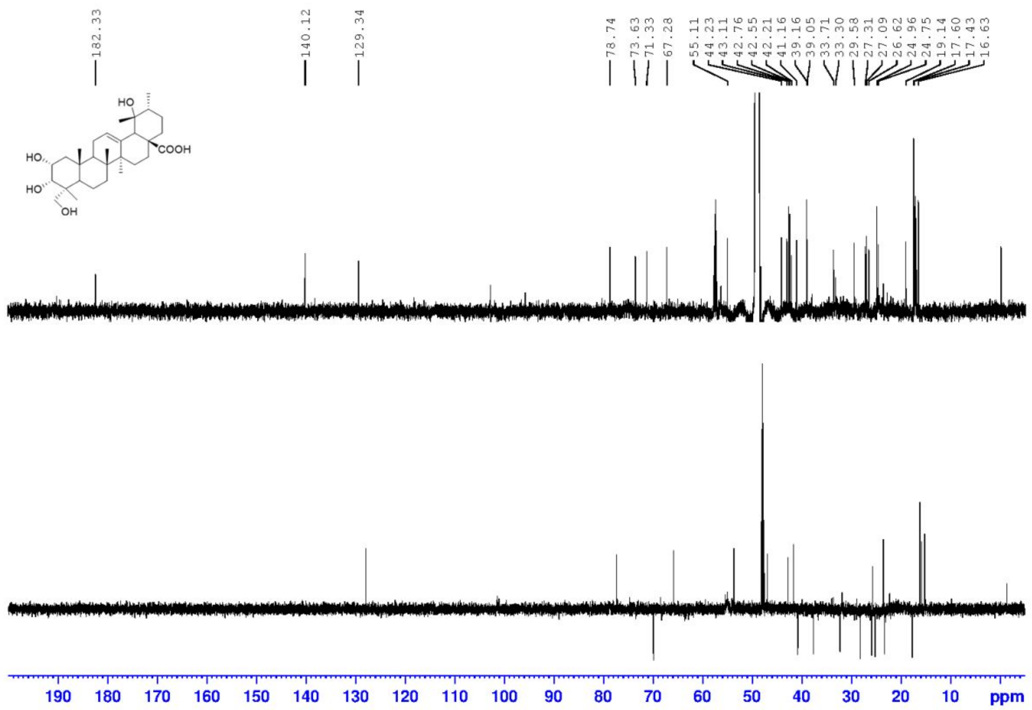

Figure S130. The ^{13}C and DEPT 135 NMR spectra of compound 31

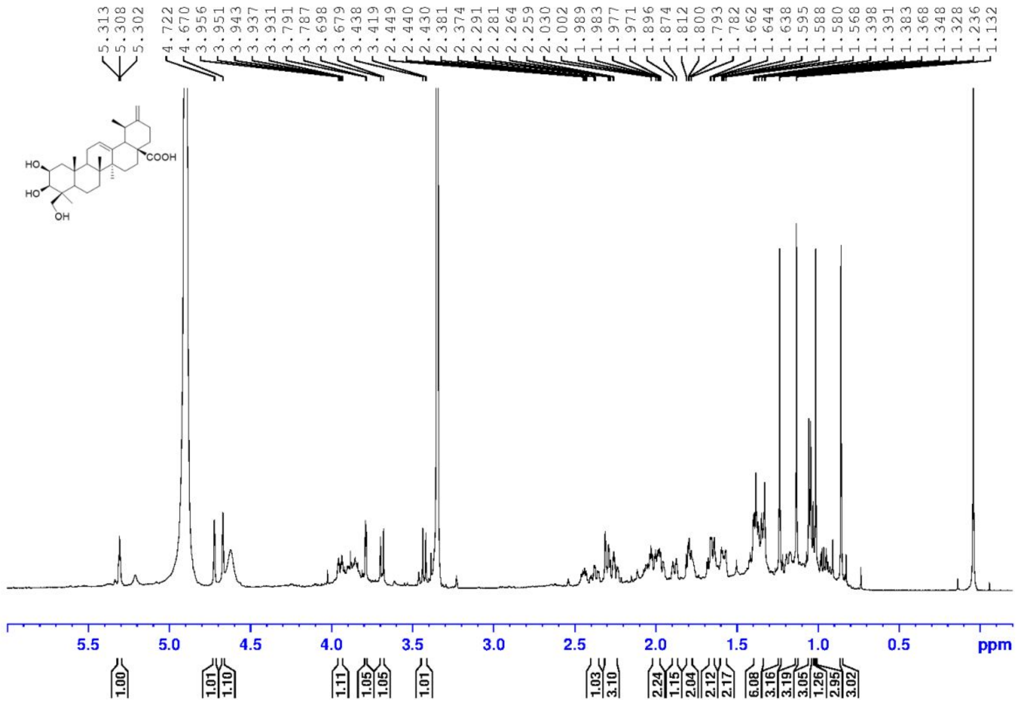

Figure S131. The ^{1}H NMR spectrum of compound 32

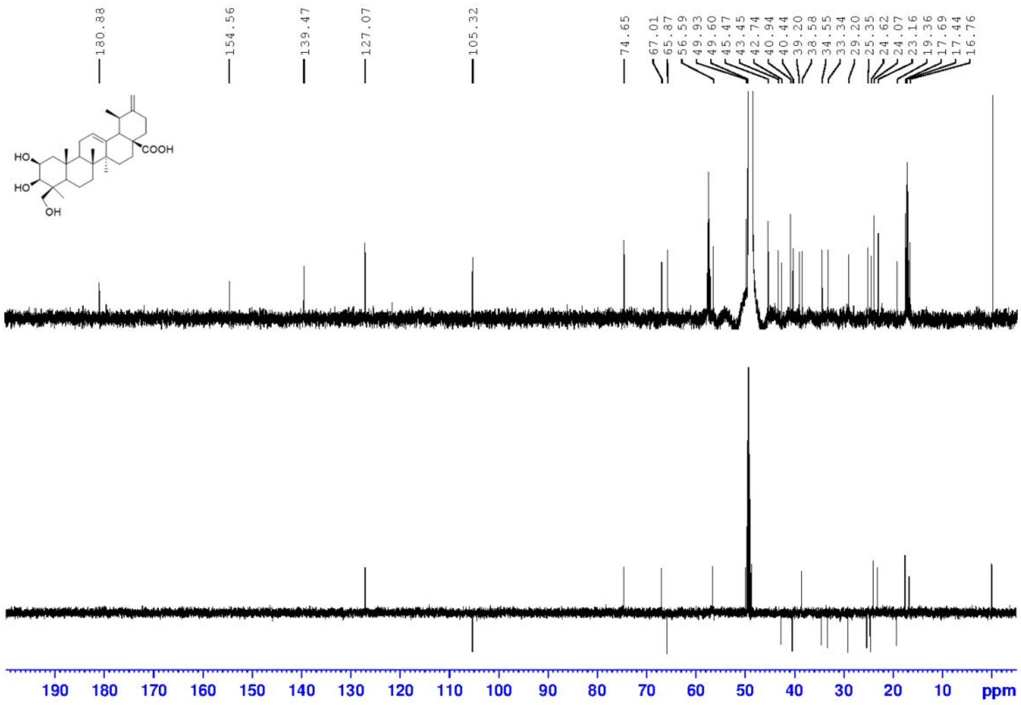

Figure S132. The ^{13}C and DEPT 135 NMR spectra of compound 32

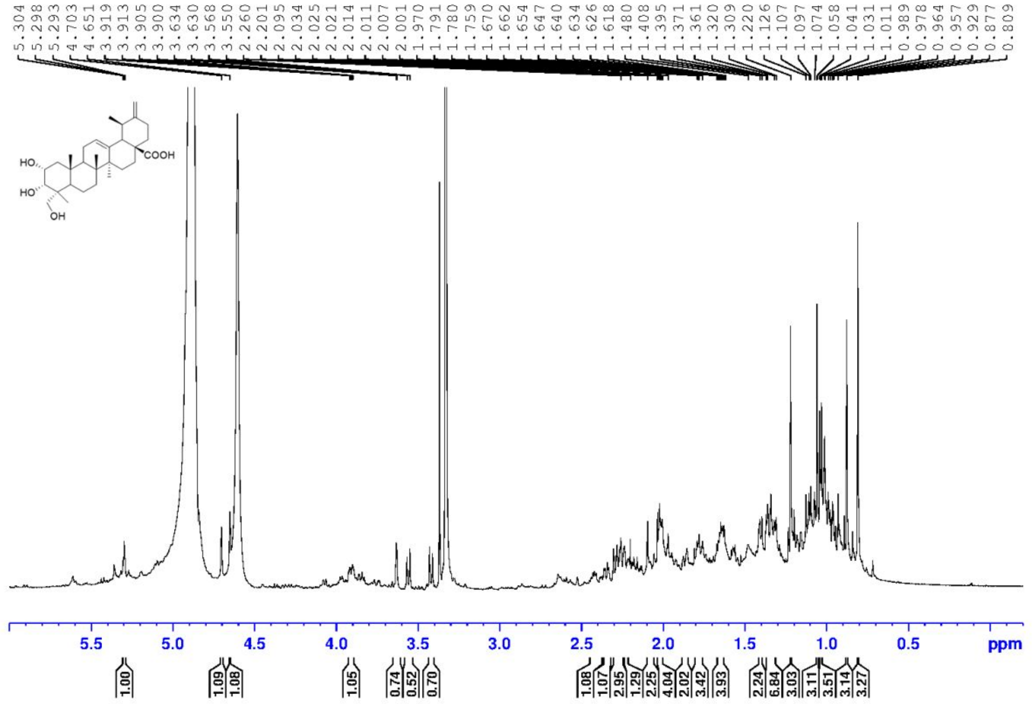

Figure S133. The ^{1}H NMR spectrum of compound 33

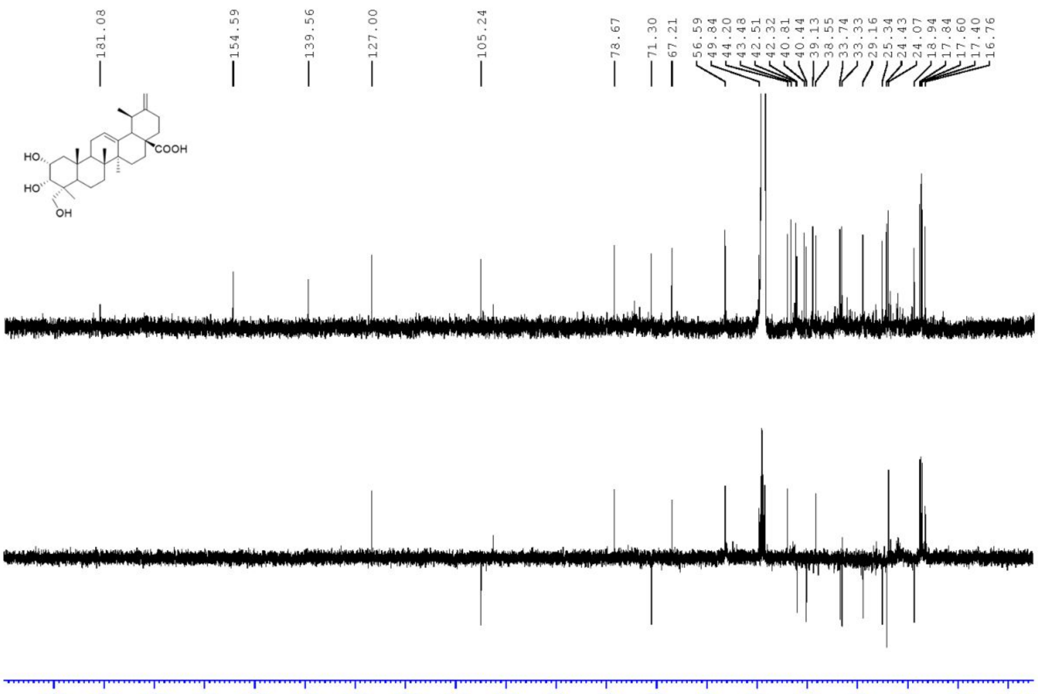

Figure S134. The ^{13}C and DEPT 135 NMR spectra of compound 33

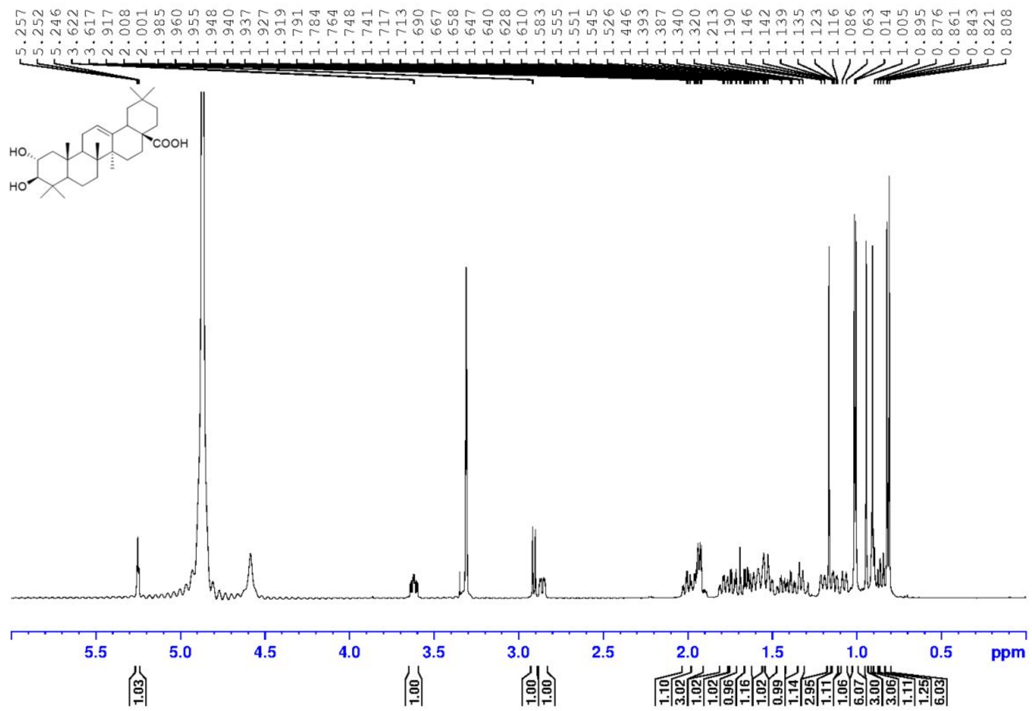

Figure S135. The ^{1}H NMR spectrum of compound 34

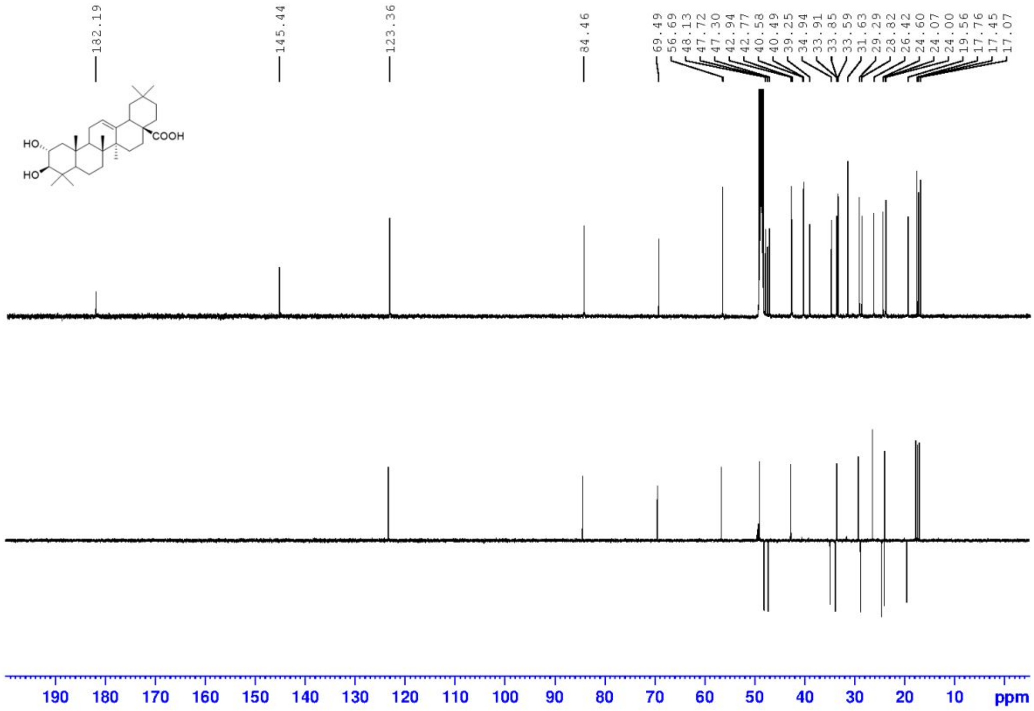

Figure S136. The ^{13}C and DEPT 135 NMR spectra of compound 34

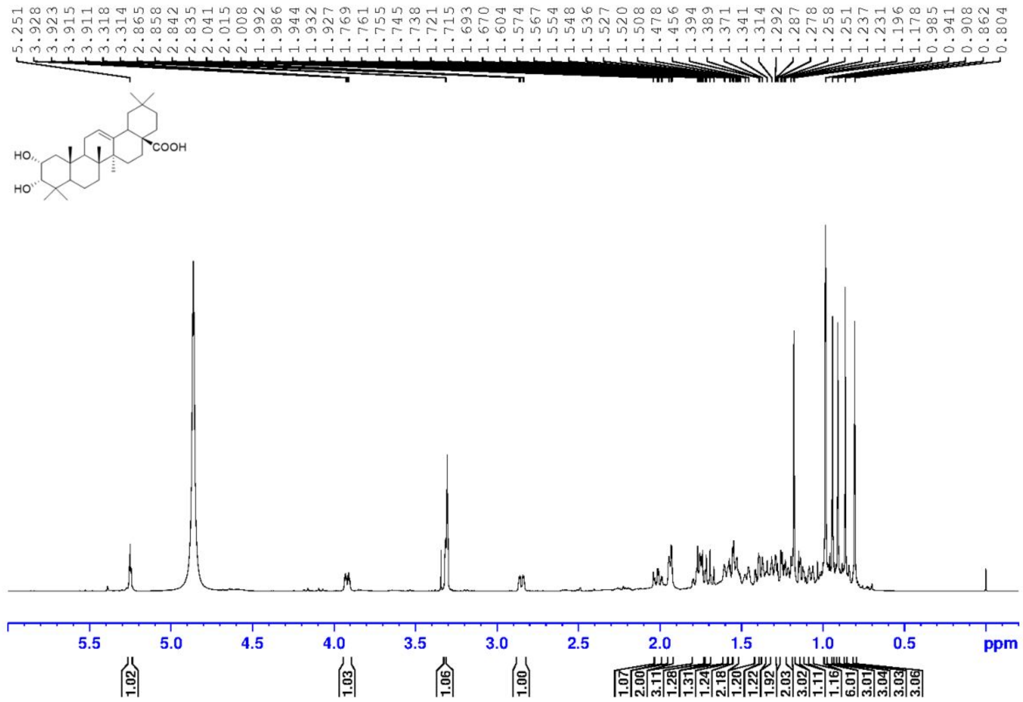

Figure S137. The ^{1}H NMR spectrum of compound 35

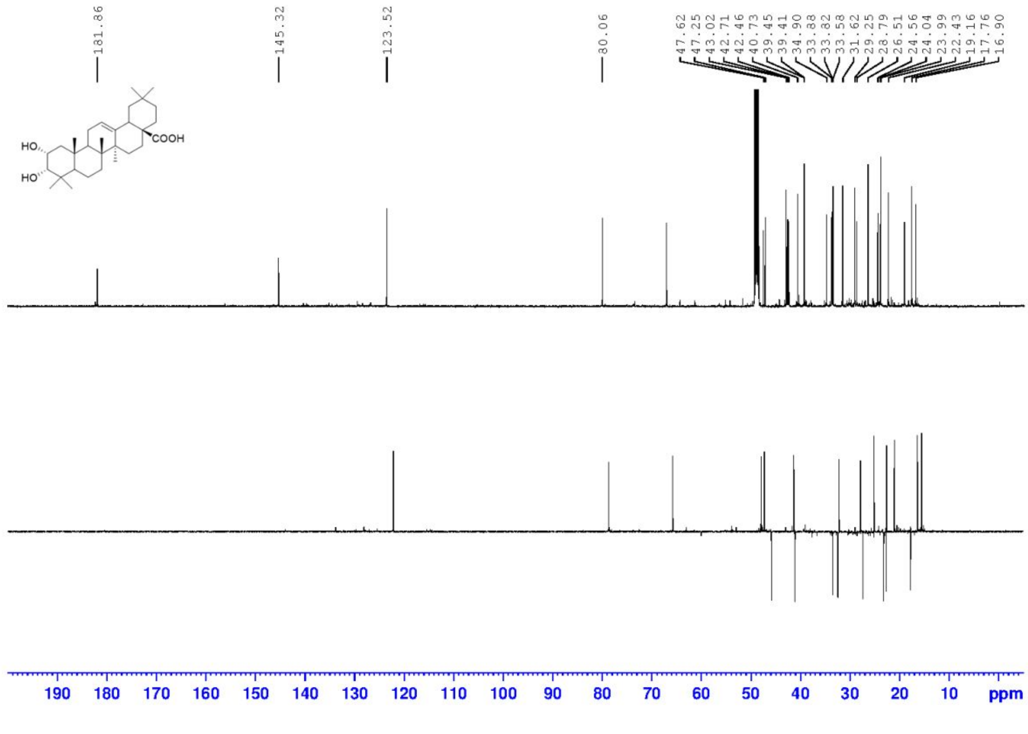

Figure S138. The ^{13}C and DEPT 135 NMR spectra of compound 35

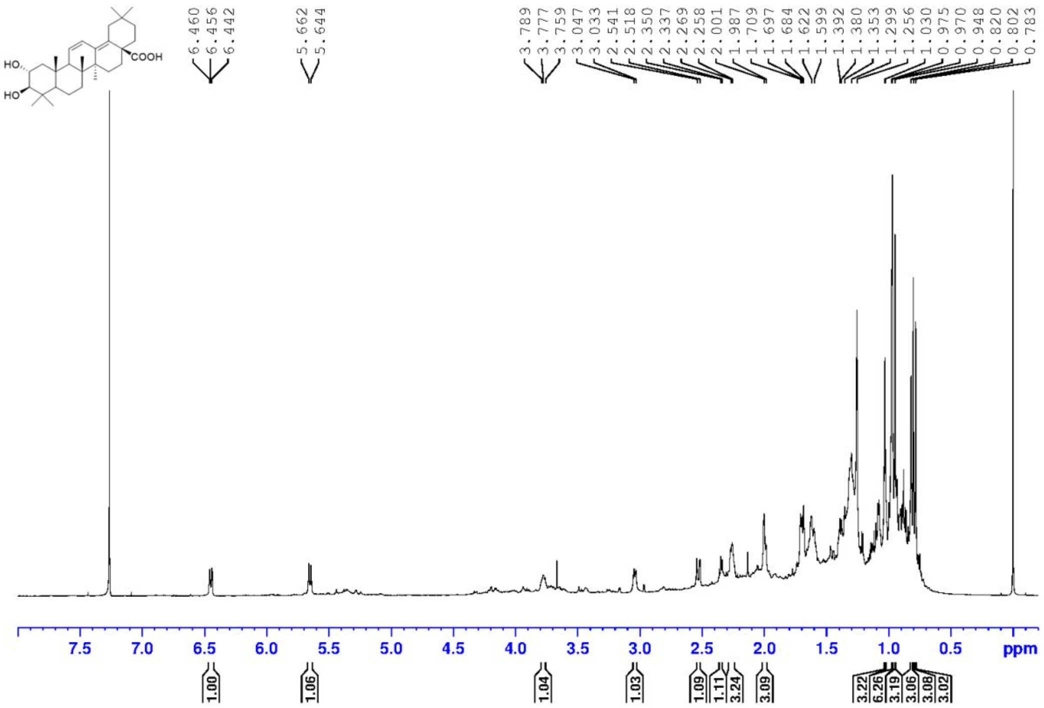

Figure S139. The ^{1}H NMR spectrum of compound 36

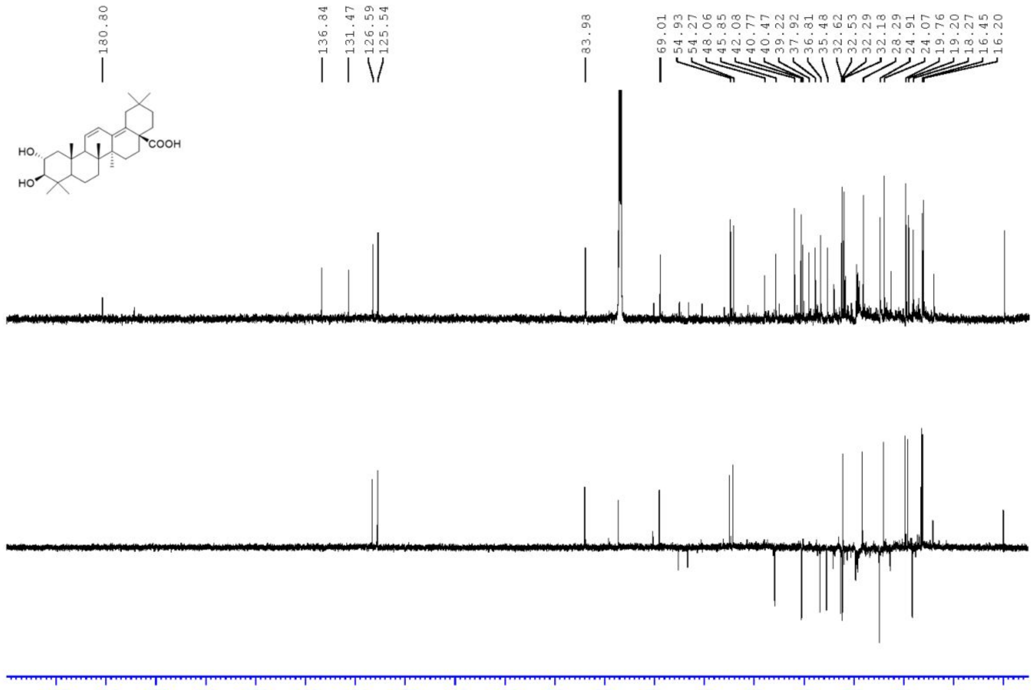

Figure S140. The ^{13}C and DEPT 135 NMR spectra of compound 36

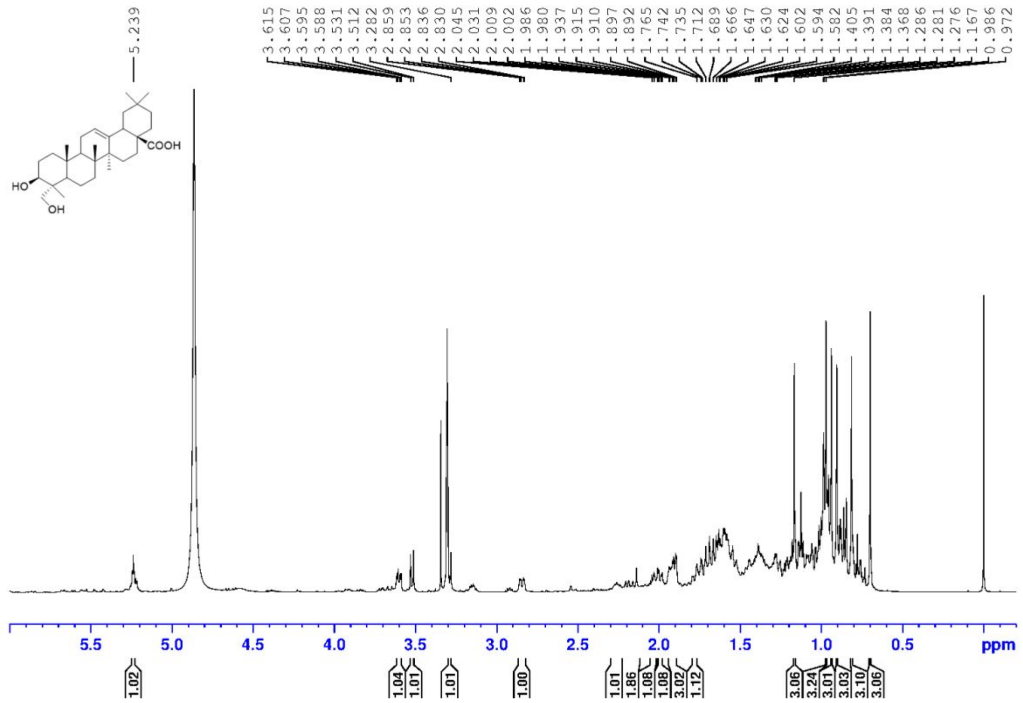

Figure S141. The ^{1}H NMR spectrum of compound 37

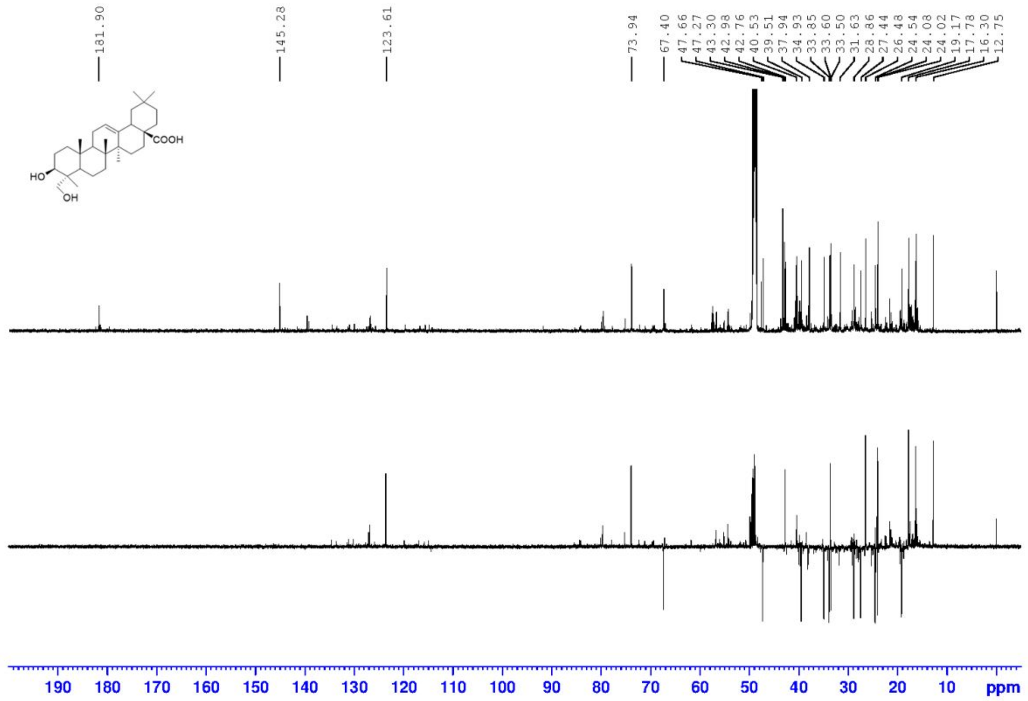

Figure S142. The ^{13}C and DEPT 135 NMR spectra of compound 37

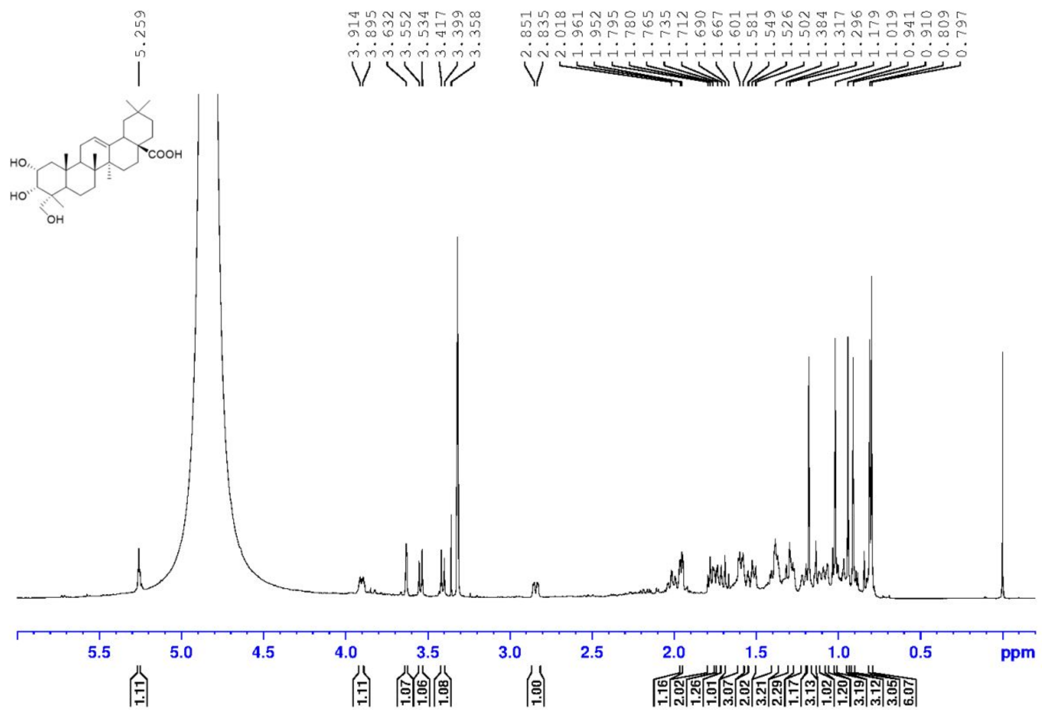

Figure S143. The ^{1}H NMR spectrum of compound 38

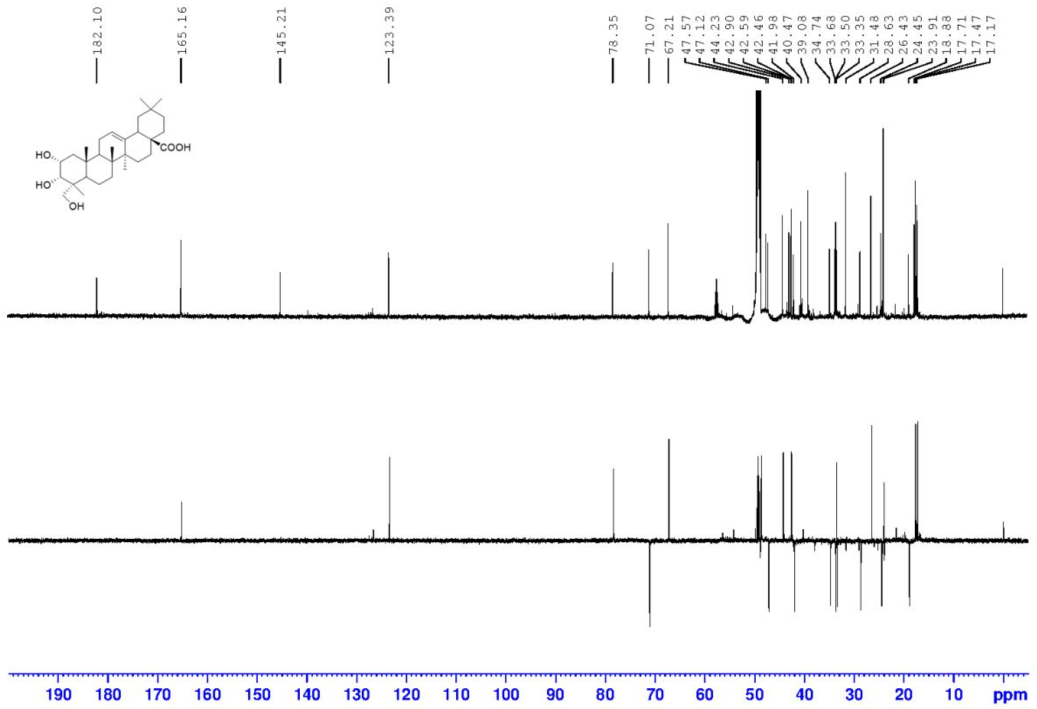

Figure S144. The ^{13}C and DEPT 135 NMR spectra of compound 38

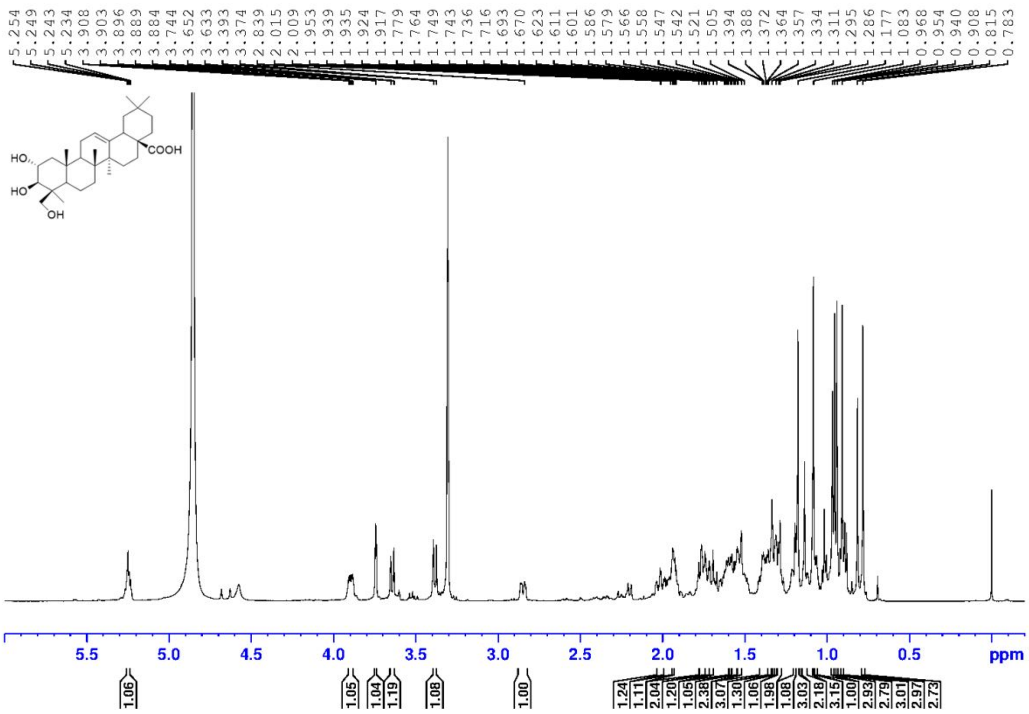

Figure S145. The ^{1}H NMR spectrum of compound 39

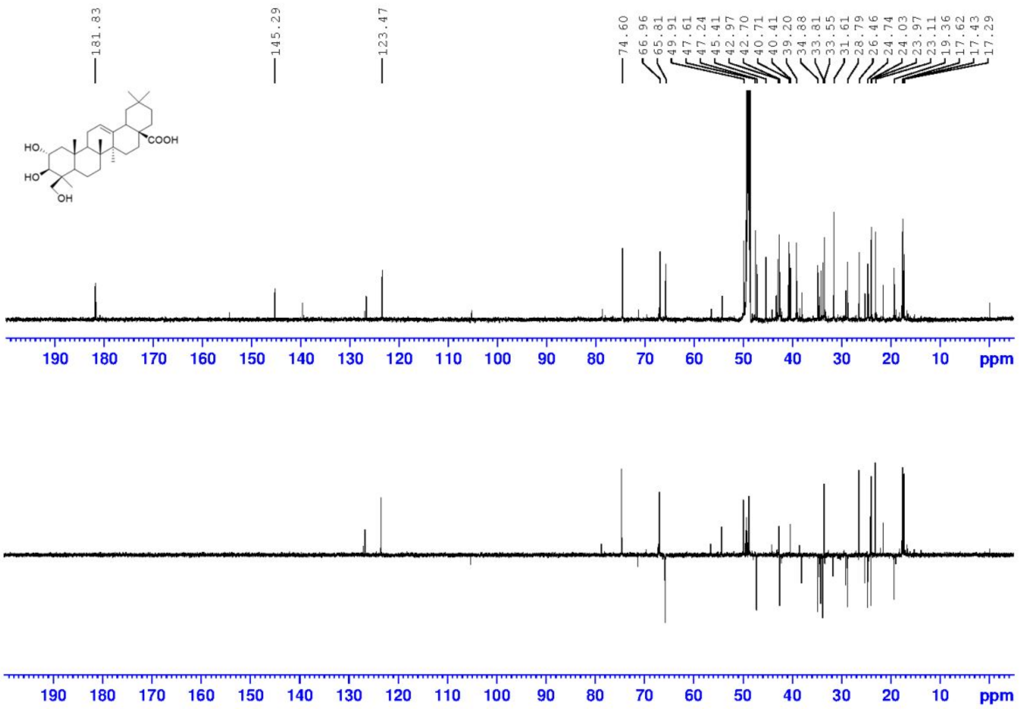

Figure S146. The ^{13}C and DEPT 135 NMR spectra of compound 39

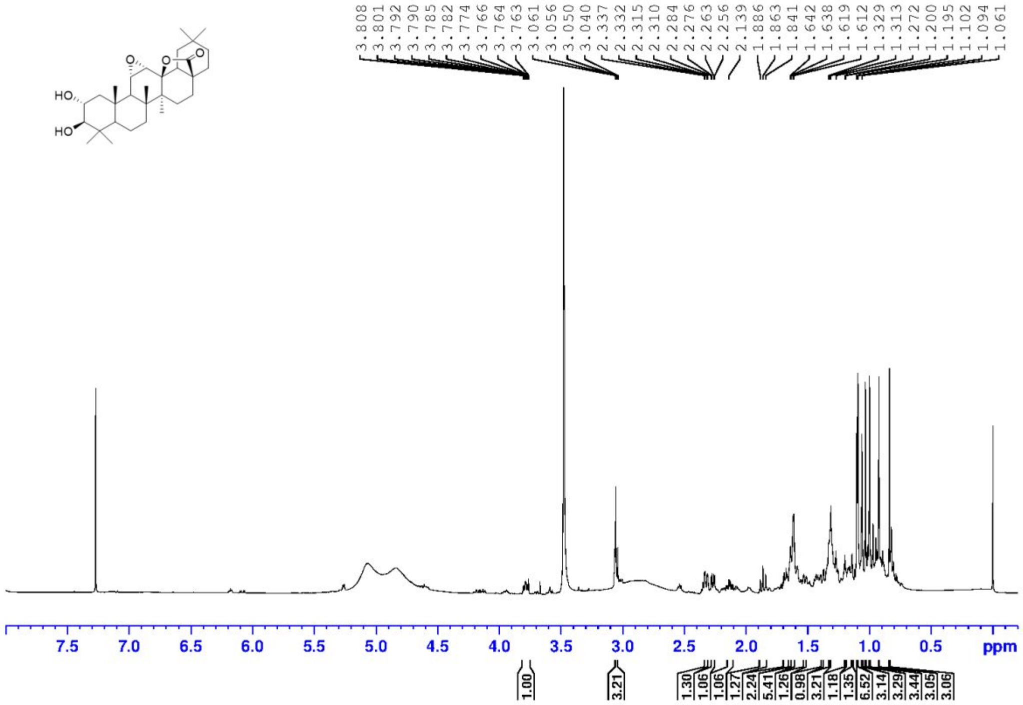

Figure S147. The ^{1}H NMR spectrum of compound 40

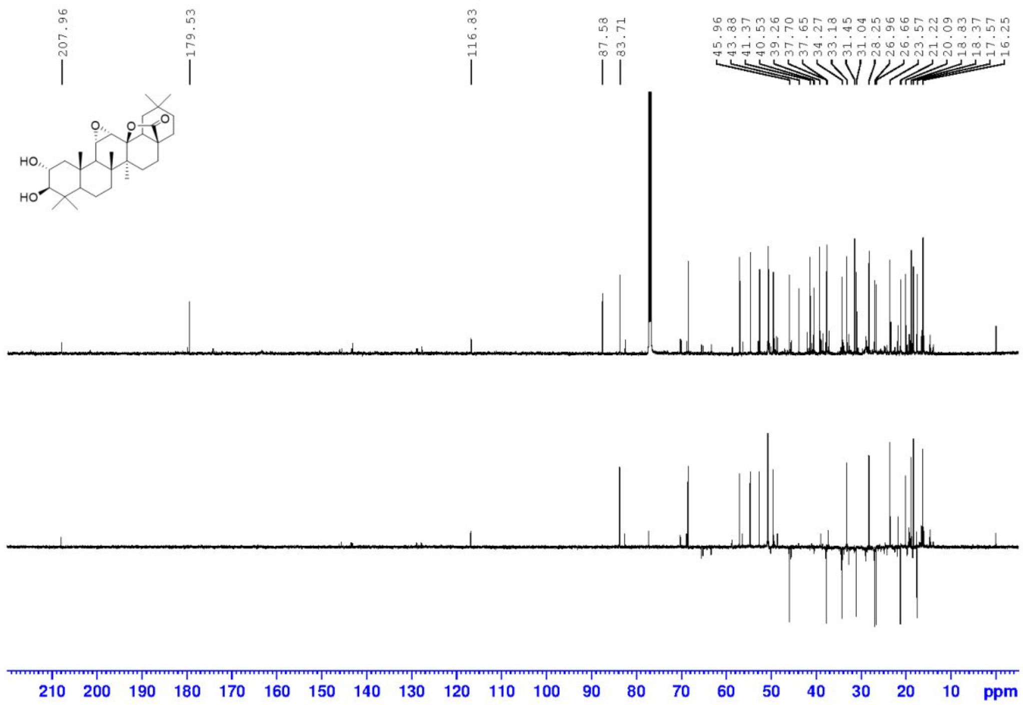

Figure S148. The ^{13}C and DEPT 135 NMR spectra of compound 40

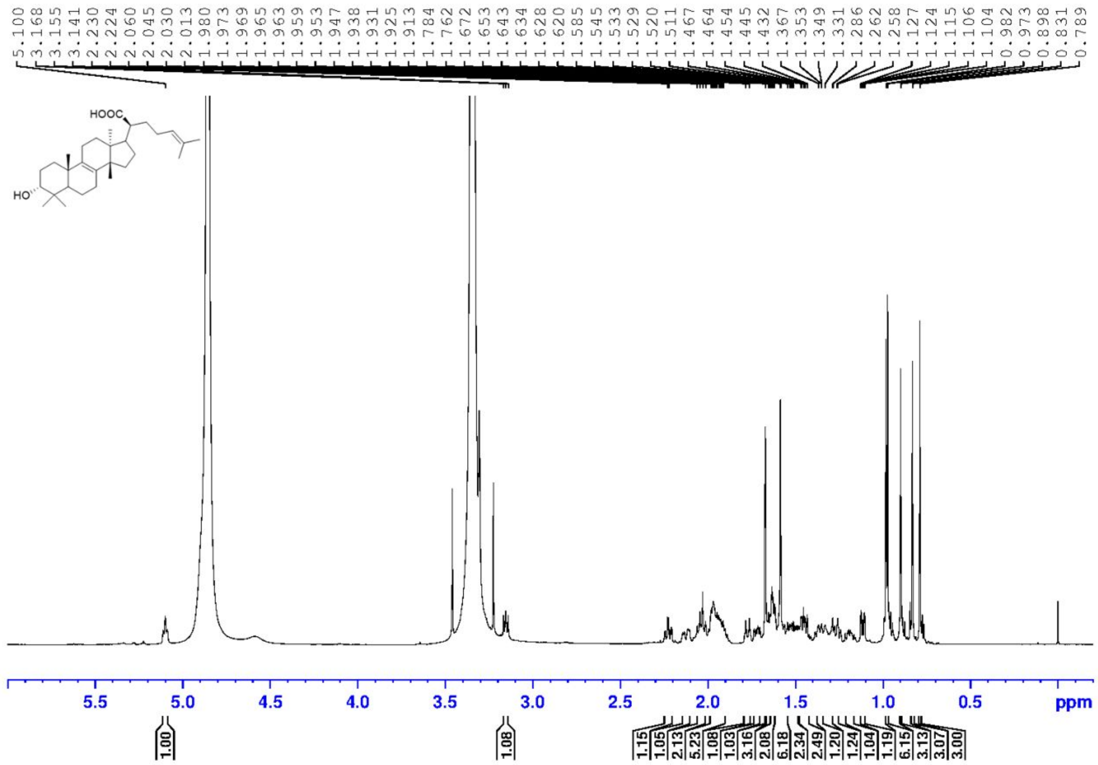

Figure S149. The ^{1}H NMR spectrum of compound 41

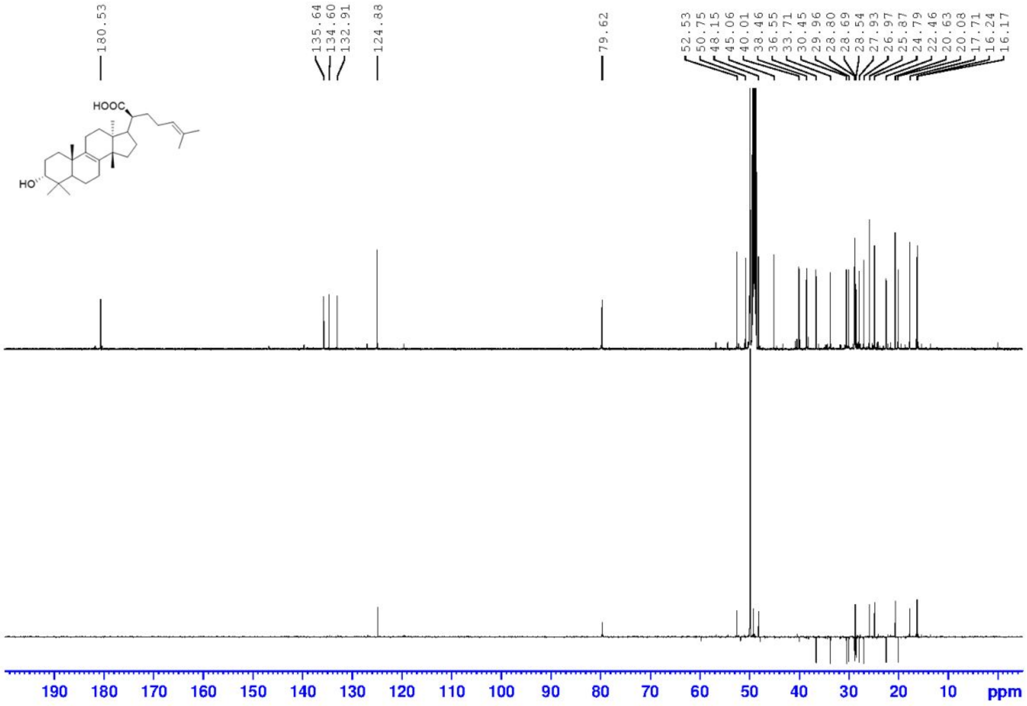

Figure S150. The ^{13}C and DEPT 135 NMR spectra of compound 41

Table S1. ^{1}H (600 MHz) and ^{13}C (150 MHz) NMR data of compound **23** in pyridine-d_5.

No.	δ_C	δ_H (δ in ppm, J in Hz)
1	40.1, CH_2	1.55, d (12.9)
		0.87, m
2	19.2, CH_2	1.99, m
		1.95, m
3	38.4, CH_2	2.25, m
		0.96, m
4	43.9, C	
5	56.6, CH	1.18, m
6	20.4, CH_2	2.01, m
		1.95, m
7	28.5, CH_2	2.21, m
		1.12, m
8	38.0, C	
9	45.1, CH	2.27, m
10	38.2, C	
11	33.3, CH_2	2.29, m
		1.36, m
12	34.7, CH_2	1.97, m
		1.93, m
13	72.0, C	
14	36.9, CH_2	2.05, m
		1.84, dd (12.5, 6.7)
15	77.1, CH	4.11, brs
16	160.0, C	
17	107.2, CH_2	6.00, t (2.2)
		5.68, t (2.1)
18	28.6, CH_3	1.17, s
19	177.8, C	
20	12.8, CH_3	0.99, s
OCH_3	51.2, CH_3	3.68, s

Table S2. ^{1}H (600 MHz) and ^{13}C (150 MHz) NMR data of compound **28** in MeOD.

No.	δ_C	δ_H (δ in ppm, J in Hz)	No.	δ_C	δ_H (δ in ppm, J in Hz)
1	35.8, CH_2	2.49, m	16	28.6, CH_2	2.19, dd (13.5, 4.0)
		1.27, dd (14.0, 4.3)			1.06, m
2	24.5, CH_2	2.24, m	17	35.1, C	
		1.58, m	18	60.5, CH	1.61, d (11.1)
3	75.0, CH	5.26, t (3.0)	19	40.6, CH	1.50, m
4	47.6, C		20	40.5, CH	1.50, m
5	51.5, CH	1.47, m	21	32.0, CH_2	1.48, m
6	20.0, CH_2	1.95, m			1.38, m
		1.80, m	22	42.0, CH_2	1.53, m
7	33.9, CH_2	1.74, dd (13.1, 4.0)			1.39, m
		1.51, m	23	24.4, CH_3	1.20, s
8	46.4, C		24	179.5, C	
9	61.7, CH	2.51, s	25	13.9, CH	1.16, s
10	38.6, C		26	18.9, CH_3	1.21, s
11	201.6, C		27	20.8, CH_3	1.40, s
12	131.2, CH	5.53, s	28	29.5, CH_3	0.87, s
13	168.0, C		29	17.8, CH_3	0.84, d (6.4)
14	45.1, C		30	21.5, CH_3	0.98, s
15	28.3, CH_2	1.96, dd (13.5, 4.0)			
		1.30, m			

Table S3. ^1H (600 MHz) and ^{13}C (150 MHz) NMR data of compound 40 in $CDCl_3$.

No.	δ_C	δ_H (δ in ppm, J in Hz)	No.	δ_C	δ_H (δ in ppm, J in Hz)
1	45.9, CH_2	2.27, dd (12.5, 4.5)	16	21.2, CH_2	2.13, m
		1.14, m			1.32, m
2	68.5, CH	3.78, m	17	43.9, C	
3	83.7, CH	3.04, m	18	49.6, CH	2.32, dd (13.6, 3.6)
4	39.3, C		19	37.7, CH_2	1.86, m
5	54.7, CH	0.82, m			1.63, m
6	17.6, CH_2	1.59, m	20	31.5, C	
		1.53, m	21	34.3, CH_2	1.37, m
7	31.1, C	1.30, m			1.33, m
		1.19, m	22	26.9, CH_2	1.62, m
8	40.5, C				1.62, m
9	50.6, CH	1.62, m	23	16.3, CH_3	0.84, s
10	37.7, C		24	28.3, CH_3	1.03, s
11	52.6, CH	3.06, m	25	18.4, CH_3	1.09, s
12	57.0, CH	3.05, m	26	20.1, CH_3	1.06, s
13	87.6, C		27	18.7, CH_3	1.10, s
14	41.4, C		28	179.5, C	
15	26.7, CH_2	1.68, m	29	23.6, CH_3	0.92, s
		1.68, m	30	33.2, CH_3	0.99, s
